# Synthesis and Biological Evaluation of (*S*)-2-(Substituted arylmethyl)-1-oxo-1,2,3,4-tetrahydropyrazino[1,2-*a*]indole-3-carboxamide Analogs and Their Synergistic Effect against PTEN-Deficient MDA-MB-468 Cells

**DOI:** 10.3390/ph14100974

**Published:** 2021-09-25

**Authors:** Ye-Mi Kwon, Sou Hyun Kim, Young-Suk Jung, Jae-Hwan Kwak

**Affiliations:** 1 College of Pharmacy, Kyungsung University, Busan 48434, Korea; kym00109@naver.com; 2 Department of Pharmacy, Research Institute for Drug Development, College of Pharmacy, Pusan National University, Busan 46241, Korea; souhyun@pusan.ac.kr

**Keywords:** anti-TNBC, PTEN-deficient cancer cells, pyrazinoindolone scaffold, synergistic effects, EGFR-TKI resistance

## Abstract

A series of twenty-six compounds of furfuryl or benzyl tetrahydropyrazino[1,2-*a*]indole analogs were synthesized and evaluated for cytotoxic activity against the estrogen receptor (ER)-positive breast cancer cell line (MCF-7) and the epidermal growth factor receptor (EGFR) over-expressed triple-negative breast cancer cell line (MDA-MB-468). Among them, compounds **2b**, **2f** and **2i** showed more potent activity and selectivity against MDA-MB-468 cells than gefitinib, as an EGFR- tyrosine kinase inhibitor. In addition, it was confirmed by means of isobologram analysis of combinational treatment with gefitinib that they have a synergistic effect, especially compounds **2b** and **2f**, which inhibit Akt T308 phosphorylation. Moreover, it was confirmed that 2-benzyl-1-oxo-1,2,3,4-tetrahydropyrazino[1,2-*a*]indole-3-carboxamide analogs (**2b**, **2f**, and Ref 2) tend to selectively inhibit PI3Kβ, which is involved in the phosphorylation of Akt.

## 1. Introduction

Cancer is a leading cause of death worldwide and is caused by the uncontrolled growth of cells in an organ of the body. Over 200 types of cancers tend to develop invasion and metastasis into other tissues [1]. In 2020, there were 19.3 million new cancer cases and almost 10.0 million cancer deaths worldwide. The incidence of cancer in men is highest in the order of lung, prostate, colorectum, stomach, and liver, and the highest percentage of cancer types in women occurs in the breast, colorectum, lung, cervix uteri, and thyroid. Breast and lung cancers are the most common types of cancer of both sexes [2]. For the treatment of cancer, small-molecule drugs have been approved by the Food and Drug Administration (FDA) and have been developed to control various therapeutic targets, including hormone receptors and oncogenic kinases, or as traditional chemotherapy [3,4,5,6].

In targeted therapy against lung cancer, many studies have focused on the more common non-small cell lung cancer (NSCLC) and involve inhibitors related to the epidermal growth factor receptor (EGFR) pathway, including the PI3K/AKT/mTOR and RAS–MAPK pathways, anaplastic lymphoma kinase (ALK), proto-oncogene ROS1, and programmed death 1 (PD-1) [7,8,9,10,11]. Among them, the EGFR pathway is considered an attractive target; thus, anti-EGFR therapies such as epidermal growth factor receptor tyrosine kinase (EGFR-TK) inhibitors and monoclonal antibodies have been developed [12,13]. Targeted therapy against breast cancer involves the overexpression of specific receptors and their downstream signaling pathways. Therefore, there are various breast cancer subtypes produced by combinations of overexpressed specific receptors such as ER+, PR+ and HER2+ breast cancer that are related to the overexpression of estrogen, progesterone receptor and human epidermal growth factor receptor2 (HER2), respectively [9,14]. Triple-negative breast cancer (TNBC) is a subtype of breast cancer that lacks the expression of estrogen, progesterone receptors and HER2 [15,16]. TNBC is more aggressive and has a poorer prognosis than other types of breast cancer because of the absence of therapeutic targets such as ER, PR, and HER2. In recent studies, EGFR has been shown to be a promising oncological target for the treatment of TNBC because it is frequently observed in TNBC compared to other breast cancer subtypes [17,18,19]. In other human solid tumors such as colorectal cancer, head and neck squamous cell carcinoma, nasopharyngeal cancer, glioblastoma, and pancreatic cancer, the overexpression of EGFR was observed and EGFR-targeting agents were approved by the FDA for the treatment of these tumors [20].

**Figure 1:**
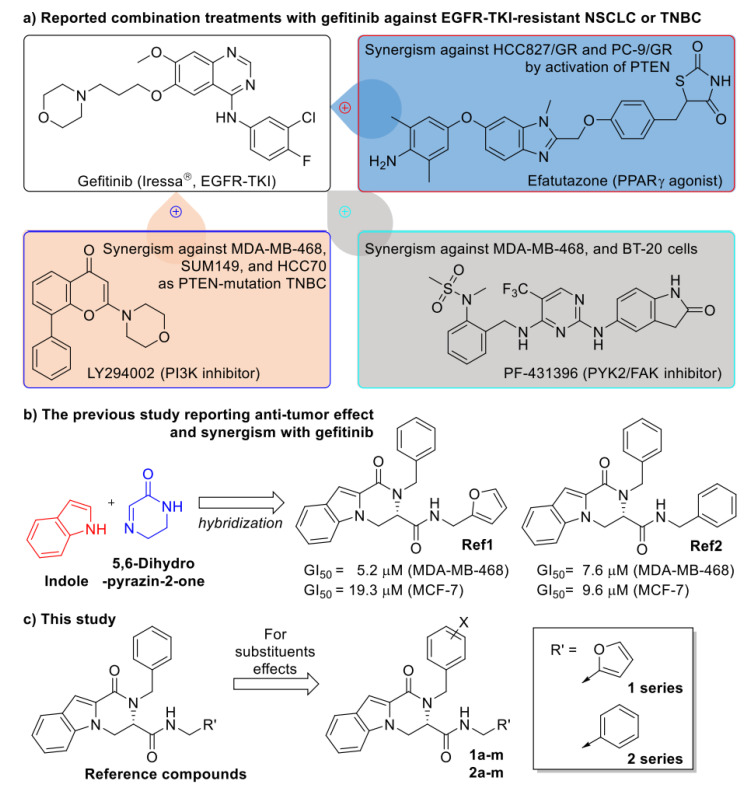
Reported compounds against EGFR-TKI-resistant cancer cell lines and design of pyrazino[1,2-a]indole-3-carboxamide. (**a**) compounds having synergistic effects with gefitinib; (**b**) the previous study of compounds Ref 1 and Ref 2; (**c**) proposal of novel pyrazino[1,2-*a*]indole-3-carboxamide analogs based on the previous study.

However, treatment with an EGFR-TK inhibitor induced resistance after a period of treatment. It was reported that the resistance was due to EGFR mutations such as T790M mutation or bypass signaling activations, including abnormal activation of downstream signaling and activation of an alternative pathway [21,22,23]. Many research groups have developed novel agents to overcome resistance to EGFR-TK inhibitors. In NSCLC, second and third-generation EGFR-TK inhibitors were prepared for tumor cells with the T790M gatekeeper mutation and PI3K/AKT/mTOR inhibitors were under clinical trials [24,25,26]. In particular, a combination of the EGFR-TK inhibitor and different molecular targeted agents, such as the PI3K/AKT/mTOR or MEK inhibitors, were presented as one of strategy to overcome the resistance [27]. In contrast, EGFR mutations in TNBC are rare, but their resistance is conferred by PIK3CA mutation and activation of the PI3K/AKT/mTOR signaling pathway [28,29,30]. In a previous study, combinational treatment with inhibitors of the PI3K/AKT/mTOR pathway and the EGFR-TK inhibitor was used to increase the therapeutic effects of synergism against TNBC with resistance [30,31]. Interestingly, MDA-MB-468 cells are basal cell carcinomas with p53 mutation and phosphatase and tensin homolog (PTEN) deletion as well as EGFR overexpression without EGFR mutation [32,33]. Because the PI3K/AKT/mTOR signaling pathway is hyper-activated by the deletion of PTEN, which acts as a tumor suppressor to regulate cell proliferation and cell death, resistance to EGFR-TK inhibitor is increased in MBA-MD-468 cells. On the other hand, inhibition of the PI3K/AKT/mTOR pathway increases susceptibility to anti-cancer effects. Therefore, agents for targeting PTEN-deficient cancers or inducing synergistic effects with EGFR-TK inhibitors have been proposed (Figure 1) [34,35,36,37,38,39].

In our previous study to overcome the resistance of EGFR-TK inhibitors, 1-oxo-1,2,3,4-tetrahydropyrazino[1,2-*a*]indole-3-carboxamide analogs were designed, synthesized, and evaluated against the MCF-7 cell line as a breast cancer subtype with hormone receptors and the MBA-MD-468 cell line as EGFR-overexpressing TNBC. Among them, reference compound 1 (Ref 1) with *N*2-benzyl and 3-furfurylamide groups showed 3.71 times more potent cell growth inhibitory activity against MDA-MB-468 cells (GI_50_: 5.2 μM) than against MCF-7 cells (GI_50_: 19.3 μM). It showed stronger activity than gefitinib against MDA-MB-468 cells. In addition, it was confirmed that there is a synergistic effect with gefitinib, an EGFR-TK inhibitor, and the compound Ref 1 that inhibits the phosphorylation of Akt on the downstream signaling pathway of EGFR [40].

Based on previous studies, we have been interested in developing specific therapeutic agents and exploring their structure–activity relationship (SAR) for patients with cancers resistant to EGFR-TK inhibitors. In this study, novel compounds were designed to confirm the relationship between anti-cancer activity and a substituent at the *N*2-position based on structure of compounds Ref 1 and Ref 2. They are synthesized and their biological evaluations are performed through various methods, including cytotoxicity, flow cytometric analysis and combinational treatment with gefitinib, western blotting and kinase assay to identify active compounds against PTEN-deficient MDA-MB-468 cells.

## 2. Results

### 2.1. Preparation of Pyrazino[1,2-a]indole-3-carboxamide Analogs

**Scheme 1:**
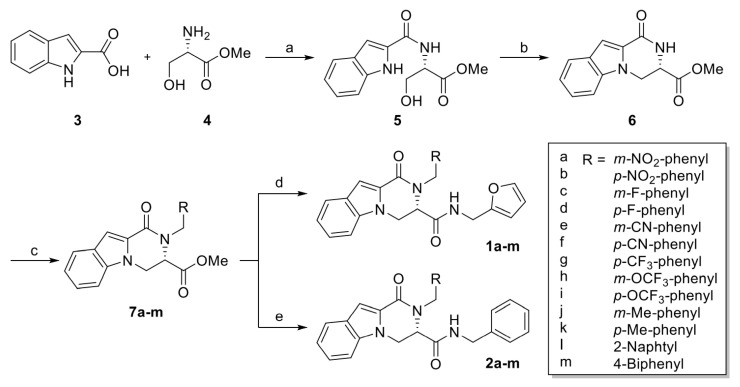
General methods for preparing (*S*)-2-(substituted arylmethyl)-1-oxo-1,2,3,4-tetrahydropyrazino[1,2-*a*]indole-3-carboxamide analogs. Reagents and conditions: (**a**) EDC, HOBt, Et_3_N, CH_2_Cl_2_, 18 h, 98%; (**b**) DEAD, PPh_3_, THF, 24 h, 54%; (**c**) arylmethyl halide, K_2_CO_3_, DMF, 5 h, 26–58%; (**d**) furfurylamine, MeOH, 15 h, 33–77%; (**e**) benzylamine, MeOH, 15 h, 28–88%.

The concise four-step synthetic procedure is described in Scheme 1 based on a previously reported study [40] for the synthesis of furfuryl and benzyl 2-(substituted arylmethyl)-1-oxo-1,2,3,4-tetrahydropyrazino[1,2-*a*]indole-3-carboxamide (series **1** and **2**, respectively). Intermediate **5** with an (*S*)-configuration was prepared in 98% yield under EDC peptide-coupling conditions with HOBt (1-hydroxybenzotriazole), and trimethylamine in CH_2_Cl_2_ from commercially available starting materials, indole-2-carboxylic acid (**3**), and L-serine methyl ester (**4**). Through an intramolecular Mitsunobu reaction using diethyl diazenedicarboxylate (DEAD) and triphenylphosphine in tetrahydrofuran, compound **6**, with a pyrazino[1,2-*a*]indole ring with (*S*)-configuration, was synthesized in 54% yield from compound **5**, based on a previous study [40]. To prepare methyl (*S*)-2-(substituted arylmethyl)-1-oxo-1,2,3,4-tetrahydropyrazino[1,2-*a*]indole-3-carboxylate analogs (**7a**–**m**), compound **6** was alkylated at the *N*2-position of the pyrazino[1,2-*a*]indole ring with various arylmethyl halides and K_2_CO_3_ in 26–58% yield. Finally, twenty-six targeted furfuryl or benzyl 2-(substituted arylmethyl)-1-oxo-1,2,3,4-tetrahydropyrazino[1,2-*a*]indole-3-carboxamide analogs (**1a**–**m** and **2a**–**m**) were prepared by direct transamidation from methyl esters (**7a**–**m**) with furfurylamine and benzylamine, respectively, in 28–88% yield. All target compounds were identified by ^1^H NMR, ^13^C NMR, and HRMS (see Supplementary Materials).

### 2.2. Evaluation of Biological Activity

#### 2.2.1. Growth Inhibitory Activity against MCF-7 and MDA-MB-468 Cell Lines

A total of twenty-six compounds of furfuryl or benzyl (*S*)-2-(substituted arylmethyl)-1-oxo-1,2,3,4-tetrahydropyrazino[1,2-*a*]indole-3-carboxamide analogs were synthesized and evaluated for cytotoxic activity against the hormone receptor-positive breast cancer cell line (MCF-7) and the EGFR-overexpressed TNBC cell line (MDA-MB-468), using a dose-dependent MTT (3-(4,5-dimethylthiazol-2-yl)-2,5-diphenyltetrazolium bromide) assay, as shown in Table 1.

In series **1** (**1a**–**1m**) with furfurylamide scaffold, all compounds except compound **1j** showed less cytotoxicity than compound Ref 1 without a substituent on the *N*2-benzyl group of the tetrahydropyrazino[1,2-*a*]indole ring against the MCF-7 cell line. Only compound **1j**, possessing a *meta*-methylbenzyl group, showed moderate activity and it (GI_50_ value: 12.2 µM) exhibited slightly more potent activity than compound Ref 1 (GI_50_ value: 19.3 µM). In addition, their activity against the MDA-MB-468 cell line was more potent than that against the MCF-7 cell line. Their activity against the MDA-MB-468 cell line was less potent than that of compound Ref 1 (GI_50_ value: 5.2 µM). Except for compound **1m**, they displayed GI_50_ values (6.57–19.9 µM) below 20 µM against the MDA-MB-468 cell line. Eight compounds that contained fluorobenzyl (**1c** and **1d**), trifluoromethylbenzyl (**1g**), trifluoromethoxybenzyl (**1h** and **1i**), methylbenzyl (**1j** and **1k**) or 2-naphthylmethyl (**1l**) exhibited GI_50_ values under 10 µM (8.03, 8.75, 8.18, 7.95, 8.34, 6.57, 9.33, and 9.52 µM, respectively) and compound **1j** among them showed a GI_50_ value of 6.57 µM, which was the most potent activity observed in the MDA-MB-468 cell line. Unlike the substituent species, their position in the *N*2-benzyl group had a low effect on GI_50_ values against the MDA-MB-468 cell line. Of the compounds with a methylbenzyl group (**1j** and **1k**), compound **1j** with the methyl group at the *meta*-position exhibited slightly more potent activity than compound **1k** with the methyl group in its *para*-position.

In series **2** (**2a**–**2m**), with a benzylamide group at the 3-position of the tetrahydropyrazino[1,2-*a*]indole moiety, several compounds, such as **2c**, **2j**, **2k**, and **2l**, showed GI_50_ values below 30 µM against the MCF-7 cell line. Only compound **2j** (GI_50_ value: 8.79 µM) and compound **2l** (GI_50_ value: 9.46 µM), with the *meta*-methylbenzyl and 2-naphthylmethyl groups at the *N*-2 position of tetrahydropyrazino[1,2-*a*]indole ring, respectively, had slightly more potent activity than compound Ref 2 (GI_50_ value: 9.6 µM), which had an *N*2-benzyl group. In the cell viability of the MDA-MB-468 cell line in series **2**, the GI_50_ values of all compounds, except for compound **2m**, were less than 30 μM, of which 10 compounds showed GI_50_ values of less than 10 μM, especially compounds including 4-NO_2_ (**2b**; GI_50_: 4.03 µM), 4-CN (**2f**; GI_50_: 2.94 µM), 4-CF_3_ (**2g**; GI_50_: 5.89 µM), 3-OCF_3_ (**2h**; GI_50_: 7.05 µM), or 4-OCF_3_ (**2i**; GI_50_: 2.96 µM) on the *N*2-benzyl ring, which had more potent inhibitory activity than the other compounds in series **2** and compound Ref 2 (GI_50_: 7.6 µM). Compounds **2f** and **2i** exhibited the best inhibitory activity and had about 2.6- and 4.4-times stronger growth inhibitory activity than compound Ref 2 and gefitinib, respectively, against the MDA-MB-468 cell line.

Regarding the selectivity of antitumor activity between the MCF-7 and MDA-MB-468 cell lines, the compounds in the MDA-MB-468 cell line were more potent (tumor-selectivity ratio from 0.91 to 10.2 or higher), except for compound **2k** (0.93) and **2l** (0.91). In series **1**, the compounds showed less growth inhibitory activity than compound Ref 1, but compound **1c** (ratio: >3.74) and **1h** (ratio: >3.77) showed higher tumor-selectivity (ratio: 3.71). In series **2**, only compounds with a methylbenzyl (**2i**, ratio: 1.07; **2j**, ratio: 0.93) or 2-naphtylmethyl (**2k**, ratio: 0.91) group at the *N*2-position of the tetrahydropyrazino[1,2-*a*]indole ring showed a lower tumor-selectivity ratio than compound Ref 2 (ratio: 1.26). Except for compounds **2j** and **2k**, nine compounds (**2a**–**i**) had a tumor-selectivity ratio from 1.07 to 10.2 or higher activity against MDA-MB-468 than MCF-7 cell lines. Substituents with heteroatoms at the *para*-position of the *N*2-benzyl group improved the selectivity of MDA-MB-468 cells. Above all, benzyl 1-oxo-1,2,3,4-tetrahydropyrazino[1,2-*a*]indole-3-carboxamide analogs with 4-CN (**2f**) or 4-OCF_3_ (**2i**) in the *N*2-benzyl group exhibited the best activity (GI_50_: 2.94 and 2.96 µM, respectively) and the best tumor selectivity (ratio: more than 10.2 and 10.1, respectively) against the MDA-MB-468 cell line as TNBC.

In conclusion, the introduction of substituents into the *N*2-benzyl ring altered its growth inhibitory activity and tumor selectivity. In particular, it was interesting to observe that they improved in series **2** with the substituents that had heteroatoms, which generally decreased in series **1**. In addition, the growth inhibitory activity of the synthesized analogs showed higher sensitivity in MDA-MB-468 cells than in MCF-7 cells, suggesting that it may act on the activated pathway only in MDA-MB-468 cells. Compounds **2b**, **2f** and **2i**, with NO_2_, CN and OCF_3_ groups at the *para*-position of the *N*2-benzyl ring, respectively, greatly increased tumor selectivity and inhibitory activity. Therefore, we performed additional biological studies to confirm the mechanism of action.

#### 2.2.2. Flow Cytometric Analysis

**Figure 2:**
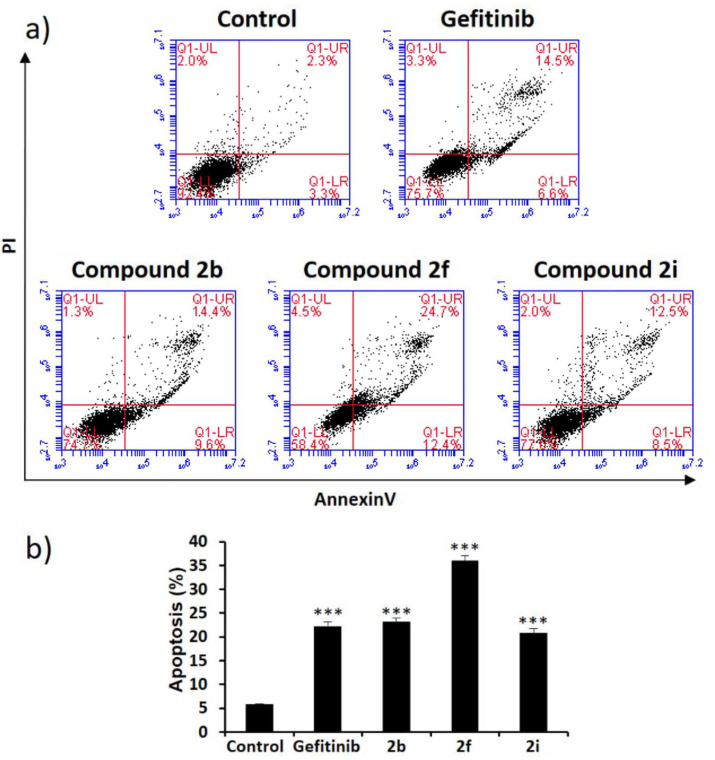
Flow cytometric analysis to determine apoptosis in MDA-MB-468 cells. (**a**) The cells were treated with 10 μM of gefitinib, and compound **2b**, **2f**, and **2i**, respectively, for 24 h. (**b**) Fluorescence-activated cell sorting (FACS) analysis of propidium iodide (PI) uptake and annexin V binding in non-permeabilized cells (lower-left, live cells; lower-right, early apoptotic cells; upper-right, late apoptotic cells; upper-left, necrotic cells). The quantification of apoptotic cells from three independent experiments. (*** *p* < 0.001, compared with control cells).

To confirm the apoptosis-inductive effect of the MDA-MB-468 cell line, compounds **2b**, **2f** and **2i**, as the groups with the most potent and tumor-selective activity, were analyzed by flow cytometric analysis with annexin V-FITC and propidium iodide (PI) (Figure 2). The rates of apoptosis induction activated by compounds **2b** and **2i** were 24.0% and 21.0%, respectively, which were similar to gefitinib (21.1%) as a positive control at a concentration of 10 µM for 24 h. Notably, compound **2f** showed 37.1% apoptosis induction, which was 1.76 times more potent than gefitinib. These results imply that compounds **2b**, **2f** and **2i**, which all showed cytotoxic activity, induce apoptosis in the form of programmed cell death in MDA-MB-468 cells.

#### 2.2.3. Combinational Treatment of Compounds **2b**, **2f** and **2i** with Gefitinib

**Figure 3:**
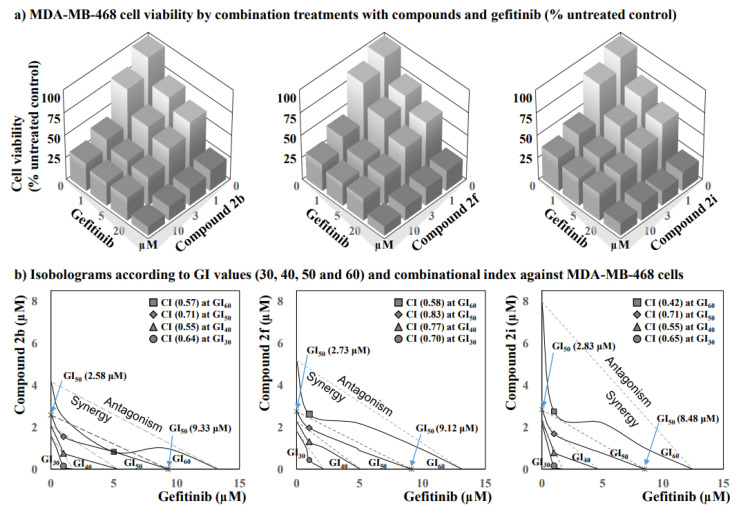
Combinational treatment with gefitinib and compounds (**2b**, **2f** and **2i**), and their isobolograms. (**a**) MDA-MB-468 cell viability by combination treatments with compounds and gefitinib (% untreated control); (**b**) isobolograms according to GI values (30, 40, 50 and 60) and combinational index against MDA-MB-468 cells.

In cancer research, combinational therapy is used a treatment to overcome single drug resistance [41,42]. Combination with inhibitors of the PI3K/AKT/mTOR pathway and the EGFR-TK inhibitor increases therapeutic effects by synergism against TNBC and NSCLC with resistance to EGFR-TK inhibitors [31,43]. In addition, our previous results confirmed that compounds Ref 1 and Ref 2 have synergistic effects in the combinational treatment of MDA-MB-468 cells with gefitinib [40]. Therefore, the synergistic effects were investigated to confirm the targeted pathway. In this study, compounds **2b**, **2f**, and **2i**, which showed potent cytotoxic activity against the MDA-MB-468 cell line as an EGFR-overexpressed cell line, were tested to confirm their specific target by combination treatment with gefitinib as an EGFR-TK inhibitor. Compounds **2b**, **2f**, and **2i** exhibited cytotoxicity at various concentrations (0, 1, 3, and 10 µM) in a dose-dependent manner against the MDA-MB-468 cell line. In addition, their combination treatment with the use of several doses (0, 1, 5, and 20 µM) of gefitinib resulted in the MDA-MB-468 cell viability graphs, as shown in Figure 3a. Generally, the addition of gefitinib decreases cell viability in a dose-dependent manner and is more cytotoxic than single treatment with compounds **2b**, **2f** and **2i**.

Based on the cell viability of the combination treatment, we performed several isobologram analyses to assess the interaction between gefitinib and the compound at concentrations of 30%, 40%, 50%, and 60% of the maximal inhibition of cell proliferation, referred to as GI_30_, GI_40_, GI_50_, and GI_60_, respectively (Figure 3b). Compounds **2b**, **2f**, and **2i** showed synergistic effects with gefitinib in all analyzed GI values. In a combinational analysis using the Chou and Talalay equation (CI = (D)_1_/(D_χ_)_1_ + (D)_2_/(D_χ_)_2_) [44], the combination index (CI) at GI_60_ was 0.57, 0.58, and 0.42 by compounds **2b**, **2f**, and **2i**, respectively, and they had more synergism than GI_30_, GI_40_, and GI_50_. At the GI_50_ values, compounds **2b**, **2f**, and **2i** with 1 µM gefitinib had the best combination index (CI) values of 0.71, 0.83, and 0.71, respectively, which indicates that they had a synergistic effect with a CI of less than 1.

Interestingly, they induced increased apoptosis and showed synergistic effects with gefitinib, as the EGFR-TK inhibitor, against MDA-MB-468 cells, as EGFR-overexpressing TNBC. MDA-MB-468 cells have been reported as cancer possessing overexpressed EGFR without mutation, but also with p53 mutation and PTEN deletion [32,33]. In the reported studies, it was shown that a synergistic effect with EGFR-TK inhibitor may occur in the case of using inhibitors of the PI3K/AKT/mTOR pathway because the PI3K/AKT/mTOR signaling pathway is hyper-activated by the deletion of PTEN [30,31]. Therefore, we evaluated their activity in the downstream signaling pathway of EGFR.

#### 2.2.4. Effects of the Compounds on Downstream Signaling of EGFR

To gain a better insight into the mechanisms underlying the activity of these compounds, the inhibitory effects of compounds **2b**, **2f**, and **2i** were tested and compared with gefitinib (GE), compound Ref 1, and Ref 2 in the downstream signaling pathway of EGFR in MDA-MB-468 cells, as an EGFR-overexpressed basal-like breast cancer cell line.

**Figure 4:**
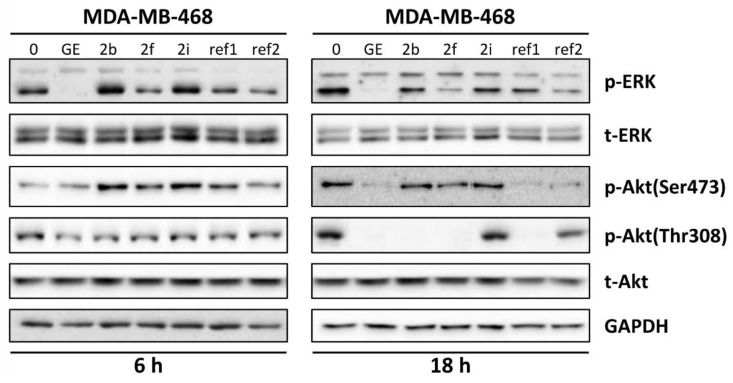
The effect of the compounds on EGFR signaling pathway in MDA-MB-468 cells. Cells were treated with 10 μM of gefitinib (GE), compound **2b**, **2f**, and **2i** for 6 h and 18 h, respectively. Protein expression of phospho-ERK, total-ERK, phospho-Akt(Ser473), phospho-Akt(Thr308) and total-Akt were detected by immunoblotting. GAPDH was used as a loading control.

In western blotting of compounds after 6 h, gefitinib, a known EGFR-TK inhibitor, reduced the phosphorylation of both ERK and Akt. However, compounds **2b** and **2i** did not suppress ERK phosphorylation or Akt Ser473 phosphorylation. The 2-arylmetyl-1-oxo-1,2,3,4-tetrahydropyrazino[1,2-*a*]indole-3-carboxamide analogs, including compounds Ref 1 and Ref 2, slightly inhibited Akt Thr308 phosphorylation and compounds **2f**, Ref 1, and Ref 2 showed inhibition of ERK phosphorylation. After 18 h, gefitinib exhibited potent inhibitory activity on the ERK and Akt pathways. Compounds Ref 1 and Ref 2 inhibit Akt Ser473 phosphorylation, as reported in a previous study. However, it was confirmed that compounds **2b**, **2f**, and **2i** did not inhibit Akt Ser473 phosphorylation. Among them, only compounds **2b**, and **2f** showed potent inhibitory activity on Akt Thr308 phosphorylation, as shown in Figure 4. In addition, compounds **2f** and Ref 2 inhibited ERK phosphorylation, although not as dramatically as gefitinib.

#### 2.2.5. Combinational Treatment of Compounds **2b**, **2f**, and **2i** with Gefitinib

Based on the results of western blotting, it was confirmed that they have inhibitory activity for the generation of phosphorylated Akt in the EGFR-overexpressed breast cancer cell line MDA-MB-468. To identify the targets of compounds that inhibit Akt phosphorylation, we tested their enzyme activity against 18 kinases including PI3K (phosphatidylinositol-3-kinase), PDK (phosphoinositide-dependent protein kinase), mTOR (mechanistic target of rapamycin), DNA-PK (DNA-dependent protein kinase), and TBK1 (TANK-binding kinase-1). These kinases directly phosphorylate Akt or induce the upstream activation of the Akt signaling pathway. Activation of PI3Ks produces PIP_3_ (phosphatidylinositol 3,4,5-triphosphate) through phosphorylation of PIP_2_ (phosphatidylinositol 4,5-bisphosphate), and PIP_3_ regulates PDK1 and sequentially induces Akt T308 phosphorylation. Akt S473 phosphorylation is also regulated by DNA-PK, PDK2, and mTORC2. In addition, TBK1 promotes the phosphorylation of Akt at S473 and T308 in a manner that is dependent on PI3K signaling [45,46,47].

As shown in Table 2, 2-arylmethyl-1-oxo-1,2,3,4-tetrahydropyrazino[1,2-*a*]indole-3-carboxamide analogs, except compound **2i**, exhibited higher inhibitory activity on PI3Kβ than other kinases at 10 µM. Compounds **2b**, **2f**, and Ref 2 showed the most potent activities on the PI3Kβ enzyme, with 71.8%, 70.9%, and 55.3% enzyme activity, respectively. Interestingly, compounds Ref 1 and Ref 2, which showed inhibitory activity of phosphorylation on both T308 and S473 in the western blot, exhibited more potent activity on PI3Kβ and DNA-PK than other kinases. Thus, the various effects of compounds, such as growth inhibitory activity, apoptosis, and inhibition of Akt phosphorylation may cautiously suggest that the inhibition of PI3Kβ is involved.

## 3. Discussion

To overcome resistance to EGFR-TK inhibitors, we designed 2-(substituted arylmethyl)-1-oxo-1,2,3,4-tetrahydropyrazino[1,2-*a*]indole-3-carboxamide analogs based on previous studies and prepared 18 novel compounds through a concise four-step synthetic procedure involving EDC-coupling, Mitsunobu, alkylation, and amidation reactions. In the MTT assay against MDA-MB-468 cells and MCF-7 cells, it was confirmed that the introduction of substituents into the *N*2-benzyl ring alters the growth inhibitory activity and tumor selectivity. Three compounds, **2b**, **2f**, and **2i**, exhibited the most potent activity (GI_50_: 4.03, 2.94, and 2.96 µM, respectively) and the best tumor selectivity (ratio: more than 7.44) against the MDA-MB-468 cell line as an EGFR-overexpressed TNBC. In particular, compound **2f** showed approximately 4.4 times stronger growth inhibitory activity and 4.4 times better selectivity on MDA-MB-468 than gefitinib, an EGFR-TK inhibitor.

In the analysis of apoptosis, compound **2f** exhibited 37.1% apoptosis induction, and was 1.76 times more potent than that of gefitinib. On MDA-MB-468 cells, which were resistant to EGFR-TK inhibitors because of PTEN mutation, combination treatment with gefitinib induced synergistic effects, and the combination index at GI_50_ showed the best effect with 1 µM gefitinib. These results are due to their activity in relation to the inhibition of Akt T308 phosphorylation, unlike the inhibition of EGFR-TK by gefitinib. It was confirmed that benzyl 2-benzyl-1-oxo-1,2,3,4-tetrahydropyrazino[1,2-*a*]indole-3-carboxamide analogs with 4-NO_2_ (**2b**), 4-CN (**2f**), or H (Ref 2) on the *N*2-benzyl ring tend to inhibit PI3Kβ among the kinase activities that are involved in the phosphorylation of Akt.

Recently, it has been reported that inhibition of the PI3Kβ isoform is very important for the progression of PTEN-null tumors [48,49]. In the PTEN-deficient TNBC cell line MDA-MB-468, a new potential therapeutic strategy for combination targeting of both EGFR and PI3Kβ was suggested to induce anti-tumor activity [50]. Therefore, these results, regarding 2-arylmethyl-1-oxo-1,2,3,4-tetrahydropyrazino[1,2-*a*]indole-3-carboxamide analogs, are expected for the development of anticancer agents against PTEN-deficient and EGFR-overexpressing cancer.

## 4. Materials and Methods

### 4.1. Chemistry

All reagents purchased from commercial sources were used without further purification. ^1^H NMR spectra were recorded on a JEOL JNM-EX400 (400 MHz). Chemical shifts were reported in ppm from tetramethylsilane (TMS) with the solvent resonance resulting from incomplete deuteration as the internal reference (CDCl_3_: 7.26 ppm) or relative to TMS (*δ* = 0.0). ^13^C NMR spectra were recorded on a JEOL JNM-EX400 (100 MHz) with complete proton decoupling. Chemical shifts were reported in ppm from tetramethylsilane with the solvent as the internal reference (CDCl_3_: 77.16 ppm). High-resolution mass spectra (HRMS) were expressed in *m*/*z* using the ESI-MS Agilent 6530 Accurate-Mass Quadruple Time-of-Flight (Q-TOF) method at the Metabolomics Research Center for Functional Materials, Kyungsung University, Republic of Korea.

Methyl (1*H*-indole-2-carbonyl)-L-serinate (**5**). To an anhydrous CH_2_Cl_2_ solution of indole-2-carboxylic acid (**3**) (389 mg, 2.41 mmol), HOBt (261 mg, 1.93 mmol) and L-serine methyl ester (**4**) (300 mg. 1.93 mmol), EDC (430 mL, 2.41 mmol) were added and cooled to 0 °C. Triethylamine was added dropwise for 30 min and then the reaction mixture was warmed to ambient temperature. After stirring for 18 h, the reaction mixture was concentrated under reduced pressure. The crude mixture was purified by flash column chromatography on silica gel (THF/DCM = 1/50) to afford the compound **5** (yield: 98%) [40]. ^1^H NMR (400 MHz, CDCl_3_) *δ* 9.81 (s, 1H, NH), 7.59 (dd, *J* = 8.0, 0.8 Hz, 1H), 7.42 (d, *J* = 7.6 Hz, 1H, NH), 7.38 (dd, *J* = 8.3, 0.8 Hz, 1H), 7.26 (td, *J* = 7.0, 1.0 Hz, 1H), 7.11 (td, *J* = 7.0, 1.0 Hz, 1H), 6.99 (dd, *J* = 2.1, 0.8 Hz, 1H), 4.89 (dt, *J* = 7.3, 3.5 Hz, 1H,), 4.04 (m, 2H), 3.75 (s, 3H).

Methyl 1-oxo-1,2,3,4-tetrahydropyrazino[1,2-*a*]indole-3-carboxylate (**6**). A mixture of methyl (1*H*-indole-2-carbonyl)-L-serinate (**5**) (504 mg, 1.92 mmol), triphenylphosphine (655 mg, 2.50 mmol), DEAD (1.10 mL, 2.50 mmol) and anhydrous THF (25 mL) was stirred for 24 h. After stirring for 24 h, the solvent was evaporated, then purification via flash column chromatography (2% THF in CH_2_Cl_2_) provided compound **6** (yield: 54%) [40]. ^1^H NMR (400 MHz, CDCl_3_) *δ* 9.10 (s, 1H, NH), 7.67 (dd, *J* = 8.0, 1.0 Hz, 1H), 7.39 (dd, *J* = 8.3, 0.9 Hz, 1H), 7.29 (td, *J* = 7.0, 1.0 Hz, 1H), 7.14 (td, *J* = 7.0, 0.9 Hz, 1H), 7.09 (m, 1H), 4.98 (dd, *J* = 10.5, 7.9 Hz, 1H), 4.74 (dd, *J* = 8.6, 7.9 Hz, 1H), 4.65 (dd, *J* = 10.5, 8.6 Hz, 1H), 3.84 (s, 3H).

#### 4.1.1. General Procedure of Methyl (*S*)-2-Arylmethyl-1-oxo-1,2,3,4-tetrahydropyrazino[1,2-*a*]indole-3-carboxylate (**7a**–**m**)

A mixture of methyl (*S*)-1-oxo-1,2,3,4-tetrahydropyrazino[1,2-*a*]indole-3-carboxylate (**6**) (140 mg, 0.573 mmol), K_2_CO_3_ (119 mg, 0.860 mmol) and anhydrous DMF (5 mL) was stirred, and then substituted arylmethyl bromide (4.01 mmol) was added. After stirring for 5 h, the solvent was evaporated, then purification via flash column chromatography provided compound **7a**–**m**.

Methyl (*S*)-2-(3-nitrobenzyl)-1-oxo-1,2,3,4-tetrahydropyrazino[1,2-*a*]indole-3-carboxylate (**7a**). Compound **7a** was synthesized according to the synthetic procedure given above, with a yield of 54%. ^1^H NMR (400 MHz, CDCl_3_) *δ* 8.12–8.04 (m, 2H), 7.71 (dt, *J* = 8.0, 0.9 Hz, 1H), 7.43–7.27 (m, 4H), 7.26 (s, 1H), 7.17 (ddd, *J* = 8.0, 5.0, 3.0 Hz, 1H), 6.10, 6.03 (ABq, *J* = 16.3 Hz, 2H), 4.95 (dd, *J* = 10.4, 7.6 Hz, 1H), 4.59 (dd, *J* = 8.6, 7.6 Hz, 1H), 4.52 (dd, *J* = 10.4, 8.6 Hz, 1H), 3.74 (s, 1H); ^13^C NMR (100 MHz, CDCl_3_) *δ* 171.6, 160.5, 148.5, 140.7, 139.2, 133.0, 129.6, 126.8, 125.4, 125.2, 122.6, 122.4, 122.2, 121.2, 110.3, 109.9, 69.1, 68.7, 53.6, 47.6.

Methyl (*S*)-2-(4-nitrobenzyl)-1-oxo-1,2,3,4-tetrahydropyrazino[1,2-*a*]indole-3-carboxylate (**7b**). Compound **7b** was synthesized according to the synthetic procedure given above, with a yield of 28%. ^1^H NMR (400 MHz, CDCl_3_) *δ* 8.09 (d, *J* = 8.7 Hz, 2H), 7.71 (d, *J* = 8.0 Hz, 1H), 7.29 (td, *J* = 7.5, 1.0 Hz, 1H), 7.25 (m, 1H), 7.21 (d, *J* = 8.7 Hz, 2H), 7.18 (td, *J* = 7.5, 1.0 zHz, 1H), 6.12, 6.03 (ABq, *J* = 16.8 Hz, 2H), 4.92 (dd, *J* = 10.4, 7.5 Hz, 1H), 4.57 (dd, *J* = 8.6, 7.5 Hz, 1H), 4.50 (dd, *J* = 10.4, 8.6 Hz, 1H), 3.73 (s, 3H); ^13^C NMR (100 MHz, CDCl_3_) *δ* 171.6, 160.5, 147.2, 146.0, 139.2, 127.6, 126.8, 125.4, 125.2, 123.9, 122.6, 121.2, 110.3, 109.9, 69.1, 68.7, 52.8, 47.8.

Methyl (*S*)-2-(3-fluorobenzyl)-1-oxo-1,2,3,4-tetrahydropyrazino[1,2-*a*]indole-3-carboxylate (**7c**). Compound **7c** was synthesized according to the synthetic procedure given above, with a yield of 51%. ^1^H NMR (400 MHz, CDCl_3_) δ 7.69 (dt, *J* = 8.0, 1.0 Hz, 1H), 7.33–7.30 (m, 1H), 7.29 (dd, *J* = 6.5, 1.2 Hz, 1H), 7.24 (d, *J* = 0.8 Hz, 1H), 7.20 (td, *J* = 7.9, 5.9 Hz, 1H), 7.16 (ddd, *J* = 8.0, 6.4, 1.5 Hz, 1H), 6.93–6.85 (m, 2H), 6.85–6.80 (m, 1H), 6.00, 5.95 (ABq, *J* = 16.3 Hz, 2H), 4.96 (dd, *J* = 10.4, 7.4 Hz, 1H), 4.60 (dd, *J* = 8.6, 7.4 Hz, 1H), 4.50 (dd, *J* = 10.4, 8.6 Hz, 1H), 3.76 (s, 3H); ^13^C NMR (100 MHz, CDCl_3_) *δ* 171.7, 163.1 (*J_FC_* = 245.8 Hz), 160.6, 141.1 (*J_FC_* = 7.1 Hz), 139.3, 130.0 (*J_FC_* = 8.3 Hz), 126.7, 125.5, 124.9, 122.5 (*J_FC_* = 2.9 Hz), 122.4, 120.9, 114.1 (*J_FC_* = 21.1 Hz), 114.0 (*J_FC_* = 22.0 Hz), 110.7, 109.6, 69.1, 68.5, 52.8, 47.7 (*J_FC_* = 2.0 Hz).

Methyl (*S*)-2-(4-fluorobenzyl)-1-oxo-1,2,3,4-tetrahydropyrazino[1,2-*a*]indole-3-carboxylate (**7d**). Compound **7d** was synthesized according to the synthetic procedure given above, with a yield of 56%. ^1^H NMR (400 MHz, CDCl_3_) *δ* 7.69 (dt, *J* = 8.0, 1.0 Hz, 1H), 7.35–7.30 (m, 1H), 7.30–7.25 (m, 1H), 7.23 (d, *J* = 0.7 Hz, 1H), 7.15 (ddd, *J* = 7.9, 6.7, 1.3 Hz, 1H), 7.11–7.06 (m, 2H), 6.95–6.88 (m, 2H), 5.98, 5.91 (ABq, *J* = 16.2 Hz, 2H), 4.96 (dd, *J* = 10.4, 7.5 Hz, 1H), 4.59 (dd, *J* = 8.6, 7.5 Hz, 1H), 4.50 (dd, *J* = 10.4, 8.6 Hz, 1H), 3.76 (s, 3H); ^13^C NMR (100 MHz, CDCl_3_) *δ* 171.7, 162.0 (*J_FC_* = 244.9 Hz), 160.7, 139.3, 134.1 (*J_FC_* = 3.1 Hz), 128.6 (*J_FC_* = 8.0 Hz), 126.8, 125.5, 124.8, 122.4, 120.8, 115.4 (*J_FC_* = 21.5 Hz), 110.8, 109.5, 69.1, 68.5, 52.7, 47.5 (*J_FC_* = 0.8 Hz).

Methyl (*S*)-2-(3-cyanobenzyl)-1-oxo-1,2,3,4-tetrahydropyrazino[1,2-*a*]indole-3-carboxylate (**7e**). Compound **7e** was synthesized according to the synthetic procedure given above, with a yield of 44%. ^1^H NMR (400 MHz, CDCl_3_) *δ* 7.71 (dt, *J* = 8.0, 1.0 Hz, 1H), 7.51–7.47 (m, 1H), 7.44–7.43 (m, 1H), 7.35–7.33 (m, 2H), 7.30–7.27 (m, 2H), 7.25 (d, *J* = 0.7 Hz, 1H), 7.17 (ddd, *J* = 8.0, 6.3, 1.7 Hz, 1H), 6.01, 5.97 (ABq, *J* = 16.3 Hz, 2H), 4.95 (dd, *J* = 10.4, 7.5 Hz, 1H), 4.59 (dd, *J* = 8.6, 7.5 Hz, 1H), 4.51 (dd, *J* = 10.4, 8.6 Hz, 1H), 3.77 (s, 3H); ^13^C NMR (100 MHz, CDCl_3_) *δ* 171.6, 160.5, 140.1, 139.2, 131.4, 131.0, 130.7, 129.4, 126.8, 125.3, 125.2, 122.6, 121.1, 118.9, 112.6, 110.3, 109.9, 69.1, 68.6, 52.8, 47.5.

Methyl (*S*)-2-(4-cyanobenzyl)-1-oxo-1,2,3,4-tetrahydropyrazino[1,2-*a*]indole-3-carboxylate (**7f**). Compound **7f** was synthesized according to the synthetic procedure given above, with a yield of 45%. ^1^H NMR (400 MHz, CDCl_3_) *δ* 7.70 (dt, *J* = 8.0, 1.0 Hz, 1H), 7.54–7.50 (m, 2H), 7.29 (ddd, *J* = 8.1, 6.7, 1.2 Hz, 1H), 7.25 (d, *J* = 0.9 Hz, 1H), 7.25–7.22 (m, 1H), 7.17 (ddd, *J* = 7.9, 6.7, 1.2 Hz, 1H), 7.16–7.13 (m, 2H), 6.08, 5.99 (ABq, *J* = 16.7 Hz, 2H), 4.92 (dd, *J* = 10.4, 7.4 Hz, 1H), 4.57 (dd, *J* = 8.7, 7.4 Hz, 1H), 4.49 (dd, *J* = 10.4, 8.6 Hz, 1H), 3.74 (s, 3H); ^13^C NMR (100 MHz, CDCl_3_) *δ* 171.5, 160.5, 144.0, 139.2, 132.4, 128.5, 127.5, 126.8, 125.4, 125.1, 122.6, 121.1, 111.0, 110.4, 109.8, 69.0, 68.6, 52.7, 47.9.

Methyl (*S*)-2-(4-trifluoromethylbenzyl)-1-oxo-1,2,3,4-tetrahydropyrazino[1,2-*a*]indole-3-carboxylate (**7g**). Compound **7g** was synthesized according to the synthetic procedure given above, with a yield of 33%. ^1^H NMR (400 MHz, CDCl_3_) *δ* 7.70 (dt, *J* = 8.0, 1.0 Hz, 1H), 7.49 (d, *J* = 8.1 Hz, 2H), 7.29–7.26 (m, 2H), 7.25 (s, 1H), 7.18–7.14 (m, 3H), 6.09, 5.99 (ABq, *J* = 16.5 Hz, 2H), 4.93 (dd, *J* = 10.4, 7.4 Hz, 1H), 4.58 (dd, *J* = 8.6, 7.4 Hz, 1H), 4.50 (dd, *J* = 10.4, 8.6 Hz, 1H), 3.72 (s, 3H); ^13^C NMR (100 MHz, CDCl_3_) *δ* 171.6, 160.6, 142.6 (*J_FC_* = 1.4 Hz), 139.3, 129.4 (*J_FC_* = 32.4 Hz), 127.0, 126.8, 125.54 (*J_FC_* = 3.8 Hz), 125.53, 124.1 (*J_FC_* = 273 Hz), 125.1, 122.5, 121.0, 110.6, 109.7, 69.09, 68.59, 52.74, 47.84.

Methyl (*S*)-2-(3-trifluoromethoxylbenzyl)-1-oxo-1,2,3,4-tetrahydropyrazino[1,2-*a*]indole-3-carboxylate (**7h**). Compound **7h** was synthesized according to the synthetic procedure given above, with a yield of 41%. ^1^H NMR (400 MHz, CDCl_3_) *δ* 7.69 (dt, *J* = 8.0, 1.0 Hz, 1H), 7.32–7.28 (m, 2H), 7.25–7.21 (m, 2H), 7.16 (ddd, *J* = 8.0, 5.8, 2.1 Hz, 1H), 7.05 (d, *J* = 7.6 Hz, 1H), 7.04 (s, 1H), 6.96 (d, *J* = 8.0 Hz, 1H), 6.01, 5.96 (ABq, *J* = 16.3 Hz, 2H), 4.95 (dd, *J* = 10.4, 7.5 Hz, 1H), 4.59 (dd, *J* = 8.6, 7.5 Hz, 1H), 4.50 (dd, *J* = 10.4, 8.6 Hz, 1H), 3.75 (s, 3H); ^13^C NMR (100 MHz, CDCl_3_) *δ* 171.7, 160.6, 149.5 (*J_FC_* = 1.8 Hz), 140.9, 139.3, 129.9, 126.8, 125.5, 125.2, 125.0, 122.5, 121.0, 120.5 (*J_FC_* = 258 Hz), 119.8 (*J_FC_* = 0.9 Hz), 119.5 (*J_FC_* = 0.9 Hz), 110.6, 109.7, 69.1, 68.6, 52.7, 47.7.

Methyl (*S*)-2-(4-trifluoromethoxylbenzyl)-1-oxo-1,2,3,4-tetrahydropyrazino[1,2-*a*]indole-3-carboxylate (**7i**). Compound **7i** was synthesized according to the synthetic procedure given above, with a yield of 41%. ^1^H NMR (400 MHz, CDCl_3_) *δ* 7.70 (dt, *J* = 8.0, 1.0 Hz, 1H), 7.32–7.27 (m, 2H), 7.24 (s, 1H), 7.16 (ddd, *J* = 8.0, 6.0, 2.0 Hz, 1H), 7.12 (d, *J* = 9.0 Hz, 2H), 7.08 (d, *J* = 9.0 Hz, 2H), 6.01, 5.95 (ABq, *J* = 16.2 Hz, 2H), 4.95 (dd, *J* = 10.4, 7.5 Hz, 1H), 4.59 (dd, *J* = 8.6, 7.5 Hz, 1H), 4.50 (dd, *J* = 10.4, 8.6 Hz, 1H), 3.74 (s, 3H); ^13^C NMR (100 MHz, CDCl_3_) *δ* 171.7, 160.6, 148.3 (*J_CF_* = 1.8 Hz), 139.3, 137.2, 128.3, 126.8, 125.5, 125.0, 122.5, 121.1 (*J_CF_* = 1.0 Hz), 120.9, 120.6 (*J_CF_* = 257 Hz), 110.7, 109.6, 69.1, 68.6, 52.7, 47.5.

Methyl (*S*)-2-(3-methylbenzyl)-1-oxo-1,2,3,4-tetrahydropyrazino[1,2-*a*]indole-3-carboxylate (**7j**). Compound **7j** was synthesized according to the synthetic procedure given above, with a yield of 45%. ^1^H NMR (400 MHz, CDCl_3_) *δ* 7.68 (d, *J* = 8.0 Hz, 1H), 7.32 (dd, *J* = 8.3, 0.8 Hz, 1H), 7.26 (dd, *J* = 8.3, 1.3 Hz, 1H), 7.24 (d, *J* = 0.6 Hz, 1H), 7.15 (dd, *J* = 7.9, 1.0 Hz, 1H), 7.11 (d, *J* = 7.7 Hz, 1H), 7.00 (d, *J* = 7.7 Hz, 1H), 6.94 (s, 1H), 6.86 (d, *J* = 7.7 Hz, 1H), 5.89 (ABq, *J* = 16.2 Hz, 2H), 4.96 (dd, *J* = 10.4, 7.6 Hz, 1H), 4.60 (dd, *J* = 8.6, 7.6 Hz, 1H), 4.50 (dd, *J* = 10.4, 8.6 Hz, 1H), 3.75 (s, 3H), 2.26 (s, 3H); ^13^C NMR (100 MHz, CDCl_3_) *δ* 171.7, 160.7, 139.4, 138.3, 138.1, 128.4, 127.9, 127.4, 126.7, 126.0, 124.7, 123.8, 122.2, 120.6, 111.0, 109.2, 69.1, 68.5, 52.7, 48.2, 21.6.

Methyl (*S*)-2-(4-methylbenzyl)-1-oxo-1,2,3,4-tetrahydropyrazino[1,2-*a*]indole-3-carboxylate (**7k**). Compound **7k** was synthesized according to the synthetic procedure given above, with a yield of 58%. ^1^H NMR (400 MHz, CDCl_3_) *δ* 7.67 (dt, *J* = 8.0, 0.8 Hz, 1H), 7.33 (dd, *J* = 8.3, 0.8 Hz, 1H), 7.25 (ddd, *J* = 8.3, 6.9, 1.2 Hz, 1H), 7.22 (d, *J* = 0.7 Hz, 1H), 7.13 (ddd, *J* = 7.9, 6.9, 1.1 Hz, 1H), 7.04 (d, *J* = 8.1 Hz, 2H), 6.99 (d, *J* = 8.1 Hz, 2H), 6.01, 5.88 (ABq, *J* = 16.1 Hz, 2H), 4.96 (dd, *J* = 10.4, 7.5 Hz, 1H), 4.59 (dd, *J* = 8.6, 7.5 Hz, 1H), 4.49 (dd, *J* = 10.4, 8.6 Hz, 1H), 3.76 (s, 3H), 2.27 (s, 3H); ^13^C NMR (100 MHz, CDCl_3_) *δ* 171.8, 160.8, 139.4, 136.7, 135.4, 129.2, 126.8, 126.8, 125.7, 124.7, 122.3, 120.7, 111.0, 109.3, 69.2, 68.5, 52.7, 47.9, 21.2.

Methyl (*S*)-2-(naphthalen-2-ylmethyl)-1-oxo-1,2,3,4-tetrahydropyrazino[1,2-*a*]indole-3-carboxylate (**7l**). Compound **7l** was synthesized according to the synthetic procedure given above, with a yield of 55%. ^1^H NMR (400 MHz, CDCl_3_) *δ* 7.80–7.67 (m, 4H), 7.48 (s, 1H), 7.44–7.39 (m, 2H), 7.36 (dd, *J* = 8.4, 1.0 Hz, 1H), 7.29–7.22 (m, 3H), 7.15 (ddd, *J* = 8.0, 6.9, 1.0 Hz, 1H), 6.21, 6.10 (ABq, *J* = 16.4 Hz, 1H), 4.95 (dd, *J* = 10.4, 7.4 Hz, 1H), 4.59 (dd, *J* = 8.6, 7.4 Hz, 1H), 4.49 (dd, *J* = 10.4, 8.6 Hz, 1H), 3.67 (s, 3H); ^13^C NMR (100 MHz, CDCl_3_) *δ* 171.7, 160.8, 139.5, 136.0, 133.4, 132.7, 128.3, 127.9, 127.7, 126.8, 126.1, 125.8, 125.8, 125.3, 125.1, 124.8, 122.3, 120.8, 111.0, 109.4, 69.2, 68.6, 52.7, 48.4.

Methyl (*S*)-2-([1,1′-biphenyl]-4-ylmethyl)-1-oxo-1,2,3,4-tetrahydropyrazino[1,2-*a*]indole-3-carboxylate (**7m**). Compound **7m** was synthesized according to the synthetic procedure given above, with a yield of 26%. ^1^H NMR (400 MHz, CDCl_3_) *δ* 7.69 (d, *J* = 7.9 Hz, 1H), 7.51 (d, *J* = 7.4 Hz, 2H), 7.45 (d, *J* = 8.2 Hz, 2H), 7.42–7.34 (m, 3H), 7.33–7.27 (m, 2H), 7.25 (s, 1H), 7.19–7.12 (m, 3H), 6.09, 5.97 (ABq, *J* = 16.2 Hz, 2H), 4.96 (dd, *J* = 10.4, 7.5 Hz, 1H), 4.60 (dd, *J* = 8.6, 7.5 Hz, 1H), 4.50 (dd, *J* = 10.4, 8.6 Hz, 1H), 3.74 (s, 3H); ^13^C NMR (100 MHz, CDCl_3_) *δ* 171.7, 160.8, 141.0, 140.1, 139.5, 137.5, 128.9, 127.32, 127.31, 127.28, 127.1, 126.8, 125.7, 124.8, 122.4, 120.8, 111.0, 109.4, 69.2, 68.6, 52.8, 48.0.

#### 4.1.2. General Procedure of (*S*)-*N*-(Furan-2-ylmethyl)-2-arylmethyl-1-oxo-1,2,3,4-tetrahydropyrazino[1,2-*a*]indol-3-carboxamide (**1a**–**m**)

To a solution of (*S*)-methyl 2-arylmethyl-1-oxo-1,2,3,4-tetrahydropyrazino[1,2-*a*]-indole-3-carboxylate (**7a**–**m**) (1.00 equiv.) in absolute methanol, furfurylamine (5.00 equiv.) was added and stirred for 15h at r.t. After evaporation, the residue was purified by column chromatography (EtOAc/hexanes = 1:2).

(*S*)-*N*-(Furan-2-ylmethyl)-2-(3-nitrobenzyl)-1-oxo-1,2,3,4-tetrahydropyrazino[1,2-*a*]indole-3-carboxamide (**1a**). Compound **1a** was synthesized according to the synthetic procedure given above, with a yield of 41%. ^1^H NMR (400 MHz, CDCl_3_) *δ* 8.07–8.04 (m, 1H), 7.98 (s, 1H), 7.74 (d, *J* = 8.0 Hz, 1H), 7.36 (t, *J* = 8.0 Hz, 1H), 7.34–7.30 (m, 2H), 7.27–7.25 (m, 2H), 7.22–7.18 (m, 2H), 6.32 (t, *J* = 5.9 Hz, 1H, NH), 6.27 (dd, *J* = 3.2, 1.9 Hz, 1H), 6.21 (d, *J* = 16.9 Hz, 1H), 6.08 (dd, *J* = 3.2, 0.8 Hz, 1H), 5.83 (d, *J* = 16.9 Hz, 1H), 4.85 (dd, *J* = 11.0, 8.6 Hz, 1H), 4.64 (dd, *J* = 11.0, 8.6 Hz, 1H), 4.53 (t, *J* = 8.6 Hz, 1H), 4.36 (dd, *J* = 15.5, 5.9 Hz, 1H), 4.15 (dd, J = 15.6, 5.9 Hz, 1H); ^13^C NMR (100 MHz, CDCl_3_) *δ* 171.2, 160.4, 150.7, 148.6, 142.3, 140.8, 139.3, 131.9, 129.8, 126.7, 125.5, 125.2, 122.8, 122.4, 121.4, 121.1, 110.5, 110.2, 110.0, 107.6, 69.5, 69.4, 47.6, 36.1; HRMS (ESI-QTOF) *m*/*z* calcd for C_24_H_21_N_4_O_5_^+^ [M+H]^+^ 445.1506, found 445.1507.

(*S*)-*N*-(Furan-2-ylmethyl)-2-(4-nitrobenzyl)-1-oxo-1,2,3,4-tetrahydropyrazino[1,2-*a*]indole-3-carboxamide (**1b**). Compound **1b** was synthesized according to the synthetic procedure given above, with a yield of 73%. ^1^H NMR (400 MHz, CDCl_3_) *δ* 8.07 (d, *J* = 8.8 Hz, 2H), 7.74 (d, *J* = 7.9 Hz, 1H), 7.32 (ddd, *J* = 8.5, 6.8, 1.1 Hz, 1H), 7.30 (d, *J* = 0.9 Hz, 1H), 7.29 (dd, *J* = 1.9, 0.9 Hz, 1H), 7.24 (dd, *J* = 8.5, 0.9 Hz, 1H), 7.21 (ddd, *J* = 7.9, 6.8, 1.1 Hz, 1H), 7.09 (d, *J* = 8.8 Hz, 2H), 6.27 (dd, *J* = 3.2, 1.9 Hz, 1H), 6.26 (d, *J* = 17.3 Hz, 1H), 6.23 (s, 1H, NH) 6.07 (dd, *J* = 3.2, 0.9 Hz, 1H), 5.79 (d, *J* = 17.3 Hz, 1H), 4.84 (dd, *J* = 11.0, 8.6 Hz, 1H), 4.63 (dd, *J* = 11.0, 8.6 Hz, 1H), 4.51 (t, *J* = 8.6 Hz, 1H), 4.31 (dd, *J* = 15.5, 5.9 Hz, 1H), 4.17 (dd, *J* = 15.5, 5.9 Hz, 1H); ^13^C NMR (100 MHz, CDCl_3_) *δ* 171.2, 160.4, 150.6, 147.2, 146.1, 142.4, 139.4, 126.7, 126.6, 125.5, 125.2, 124.1, 122.8, 121.4, 110.5, 110.2, 110.0, 107.6, 69.5, 69.4, 47.8, 36.0; HRMS (ESI-QTOF) *m*/*z* calcd for C_24_H_21_N_4_O_5_^+^ [M+H]^+^ 445.1506, found 445.1510.

(*S*)-*N*-(Furan-2-ylmethyl)-2-(3-fluorobenzyl)-1-oxo-1,2,3,4-tetrahydropyrazino[1,2-*a*]indole-3-carboxamide (**1c**). Compound **1c** was synthesized according to the synthetic procedure given above, with a yield of 54%. ^1^H NMR (400 MHz, CDCl_3_) *δ* 7.73 (dt, *J* = 8.0, 1.0 Hz, 1H), 7.33–7.29 (m, 3H), 7.28 (s, 1H), 7.22–7.17 (m, 2H), 6.87 (td, *J* = 8.4, 2.6 Hz, 1H), 6.75 (ddd, *J* = 7.7, 1.7, 0.9 Hz, 1H), 6.62 (dt, *J* = 9.7, 2.1 Hz, 1H), 6.35 (t, *J* = 5.9 Hz, 1H, NH), 6.29 (dd, *J* = 3.2, 1.8 Hz, 1H), 6.16 (d, *J* = 16.8 Hz, 1H), 6.09 (dd, *J* = 3.2, 0.9 Hz, 1H), 5.71 (d, *J* = 16.8 Hz, 1H), 4.85 (dd, *J* = 11.0, 8.4 Hz, 1H), 4.62 (dd, *J* = 11.0, 8.4 Hz, 1H), 4.50 (t, *J* = 8.4 Hz, 1H), 4.35 (dd, *J* = 15.6, 5.9 Hz, 1H), 4.11 (dd, *J* = 15.6, 5.9 Hz, 1H); ^13^C NMR (100 MHz, CDCl_3_) *δ* 171.5, 163.3 (*J_FC_* = 247 Hz), 160.3, 151.0, 142.2, 141.4 (*J_FC_* = 6.9 Hz), 139.6, 130.4 (*J_FC_* = 8.2 Hz), 126.5, 125.3, 125.2, 122.6, 121.4 (*J_FC_* = 2.9 Hz), 121.1, 114.2 (*J_FC_* = 21.1 Hz), 112.9 (*J_FC_* = 22.2 Hz), 110.5, 110.3, 109.9, 107.4, 69.5, 69.4, 47.7 (*J_FC_* = 2.0 Hz), 36.1; HRMS (ESI-QTOF) *m*/*z* calcd for C_24_H_21_FN_3_O_3_^+^ [M+H]^+^ 418.1561, found 418.1566.

(*S*)-*N*-(Furan-2-ylmethyl)-2-(4-fluorobenzyl)-1-oxo-1,2,3,4-tetrahydropyrazino[1,2-*a*]indole-3-carboxamide (**1d**). Compound **1d** was synthesized according to the synthetic procedure given above, with a yield of 67%. ^1^H NMR (400 MHz, CDCl_3_) *δ* 7.72 (d, *J* = 8.1 Hz, 1H), 7.33 – 7.29 (m, 3H), 7.26 (s, 1H), 7.18 (dt, *J* = 8.0, 4.0 Hz, 1H), 6.95–6.85 (m, 4H), 6.35 (t, *J* = 6.0 Hz, 1H, NH), 6.30 (dd, *J* = 3.3, 1.9 Hz, 1H), 6.18–6.06 (m, 2H), 5.66 (d, *J* = 16.5 Hz, 1H), 4.86 (dd, *J* = 11.0, 8.4 Hz, 1H), 4.62 (dd, *J* = 11.0, 8.4 Hz, 1H), 4.50 (t, *J* = 8.4 Hz, 1H), 4.35 (dd, *J* = 15.5, 6.0 Hz, 1H), 4.14 (dd, *J* = 15.5, 6.0 Hz, 1H); ^13^C NMR (100 MHz, CDCl_3_) *δ* 171.5, 161.8 (*J_FC_* = 245 Hz), 160.4, 150.9, 142.4, 139.6, 134.3 (*J_FC_* = 3.1 Hz), 127.4 (*J_FC_* = 7.9 Hz), 126.6, 125.3, 125.2, 122.6, 121.1, 115.8 (*J_FC_* = 21.5 Hz), 110.5, 110.4, 109.8, 107.5, 69.5, 69.4, 47.5, 36.1; HRMS (ESI-QTOF) *m*/*z* calcd for C_24_H_21_FN_3_O_3_^+^ [M+H]^+^ 418.1561, found 418.1565.

(*S*)-*N*-(Furan-2-ylmethyl)-2-(3-cyanobenzyl)-1-oxo-1,2,3,4-tetrahydropyrazino[1,2-*a*]indole-3-carboxamide (**1e**). Compound **1e** was synthesized according to the synthetic procedure given above, with a yield of 44%. ^1^H NMR (400 MHz, CDCl_3_) *δ* 7.74 (d, *J* = 7.9 Hz, 1H), 7.48 (d, *J* = 7.6 Hz, 1H), 7.35–7.32 (m, 2H), 7.32–7.28 (m, 2H), 7.26–7.23 (m, 2H), 7.23–7.17 (m, 2H), 6.34–6.29 (m, 1H), 6.28 (t, *J* = 5.9 Hz, 1H, NH), 6.12 (d, *J* = 16.9 Hz, 1H), 6.12 (dt, *J* = 3.2, 0.8 Hz, 2H), 5.80 (d, *J* = 16.9 Hz, 1H), 4.84 (dd, *J* = 11.0, 8.6 Hz, 1H), 4.63 (dd, *J* = 11.0, 8.6 Hz, 1H), 4.52 (t, *J* = 8.6 Hz, 1H), 4.37 (dd, *J* = 15.6, 5.9 Hz, 1H), 4.17 (dd, *J* = 15.6, 5.9 Hz, 1H); ^13^C NMR (100 MHz, CDCl_3_) *δ* 171.3, 160.4, 150.8, 142.4, 140.3, 139.4, 131.1, 130.3, 129.6, 129.4, 126.7, 125.6, 125.2, 122.8, 121.4, 118.7, 113.1, 110.6, 110.2, 110.1, 107.7, 69.5, 69.4, 47.6, 36.1; HRMS (ESI-QTOF) *m*/*z* calcd for C_25_H_21_N_4_O_3_^+^ [M+H]^+^ 425.1608, found 425.1610.

(*S*)-*N*-(Furan-2-ylmethyl)-2-(4-cyanobenzyl)-1-oxo-1,2,3,4-tetrahydropyrazino[1,2-*a*]indole-3-carboxamide (**1f**). Compound **1f** was synthesized according to the synthetic procedure given above, with a yield of 33%. ^1^H NMR (400 MHz, CDCl_3_) *δ* 7.73 (d, *J* = 8.0 Hz, 1H), 7.47 (d, *J* = 8.2 Hz, 2H), 7.37–7.34 (m, 1H), 7.32 (ddd, *J* = 8.4, 6.9, 1.2 Hz, 1H), 7.29 (s, 1H), 7.24 (d, *J* = 8.0 Hz, 1H), 7.22–7.17 (m, 1H), 7.04 (d, *J* = 8.2 Hz, 2H), 6.35 (dd, *J* = 3.1, 1.9 Hz, 1H), 6.26 (t, *J* = 5.8 Hz, 1H, NH), 6.21 (d, *J* = 17.2 Hz, 1H), 6.15 (d, *J* = 3.1 Hz, 1H), 5.75 (d, *J* = 17.2 Hz, 1H), 4.84 (dd, *J* = 11.0, 8.6 Hz, 1H), 4.63 (dd, *J* = 11.0, 8.6 Hz, 1H), 4.51 (t, *J* = 8.6 Hz, 1H), 4.35 (dd, *J* = 15.5, 5.8 Hz, 1H), 4.17 (dd, *J* = 15.5, 5.8 Hz, 1H); ^13^C NMR (100 MHz, CDCl_3_) *δ* 171.3, 160.4, 150.7, 144.2, 142.5, 139.4, 132.7, 126.7, 126.6, 125.5, 125.2, 122.8 121.4, 118.7, 111.3, 110.7, 110.12, 110.08, 107.8, 69.5, 69.4, 48.0, 36.1; HRMS (ESI-QTOF) *m*/*z* calcd for C_25_H_21_N_4_O_3_^+^ [M+H]^+^ 425.1608, found 425.1607.

(*S*)-*N*-(Furan-2-ylmethyl)-2-(4-trifluoromethylbenzyl)-1-oxo-1,2,3,4-tetrahydropyrazino[1,2-*a*]indole-3-carboxamide (**1g**). Compound **1g** was synthesized according to the synthetic procedure given above, with a yield of 77%. ^1^H NMR (400 MHz, CDCl_3_) *δ* 7.74 (d, *J* = 7.8 Hz, 1H), 7.49 (d, *J* = 8.0 Hz, 2H), 7.34–7.29 (m, 3H), 7.28 (s, 1H), 7.23–7.16 (m, 1H), 7.07 (d, *J* = 8.0 Hz, 2H), 6.34 (t, *J* = 6.0 Hz, 1H, NH), 6.29 (dd, *J* = 3.2, 1.9 Hz, 1H), 6.25 (d, *J* = 16.9 Hz, 1H), 6.10 (d, *J* = 3.2 Hz, 1H), 5.76 (d, *J* = 16.9 Hz, 1H), 4.85 (dd, *J* = 11.0, 8.5 Hz, 1H), 4.62 (dd, *J* = 11.0, 8.5 Hz, 1H), 4.51 (t, *J* = 8.5 Hz, 1H), 4.35 (dd, *J* = 15.6, 6.4 Hz, 1H), 4.09 (dd, *J* = 15.6, 5.5 Hz, 1H); ^13^C NMR (100 MHz, CDCl_3_) *δ* 171.4, 160.4, 150.9, 142.8 (*J_FC_* = 1.4 Hz), 142.4, 139.5, 129.6 (*J_FC_* = 32.5 Hz), 126.7, 126.2, 125.9 (*J_FC_* = 3.8 Hz), 125.4, 125.3, 124.1 (*J_FC_* = 272 Hz), 122.7, 121.3, 110.5, 110.2, 110.0, 107.5, 69.5, 69.4, 47.9, 36.0; HRMS (ESI-QTOF) *m*/*z* calcd for C_25_H_21_F_3_N_3_O_3_^+^ [M+H]^+^ 468.1530, found 468.1537.

(*S*)-*N*-(Furan-2-ylmethyl)-2-(3-trifluoromethoxybenzyl)-1-oxo-1,2,3,4-tetrahydropyrazino[1,2-*a*]indole-3-carboxamide (**1h**). Compound **1h** was synthesized according to the synthetic procedure given above, with a yield of 62%. ^1^H NMR (400 MHz, CDCl_3_) *δ* 7.73 (dt, *J* = 8.3, 0.9 Hz, 1H), 7.34–7.29 (m, 3H), 7.28 (s, 1H), 7.23–7.16 (m, 2H), 7.08–7.02 (m, 1H), 6.88 (s, 1H), 6.83 (ddd, *J* = 7.7, 1.7, 0.9 Hz, 1H), 6.35 (t, *J* = 5.9 Hz, 1H, NH), 6.28 (dd, *J* = 3.2, 1.9 Hz, 1H), 6.13 (d, *J* = 16.9 Hz, 1H), 6.09 (dd, *J* = 3.2, 0.8 Hz, 1H), 5.76 (d, *J* = 16.9 Hz, 1H), 4.85 (dd, *J* = 11.0, 8.5 Hz, 1H), 4.62 (dd, *J* = 11.0, 8.5 Hz, 1H), 4.51 (t, *J* = 8.5 Hz, 1H), 4.35 (dd, *J* = 15.6, 5.9 Hz, 1H), 4.14 (dd, *J* = 15.6, 5.9 Hz, 1H); ^13^C NMR (100 MHz, CDCl_3_) *δ* 171.4, 160.4, 151.0, 149.8 (*J_FC_* = 1.8 Hz), 142.3, 141.2, 139.5, 130.3, 126.6, 125.4, 125.3, 124.1, 122.6, 121.2, 120.5 (*J_FC_* = 258 Hz), 119.4, 118.5, 110.5, 110.3, 110.0, 107.4, 69.5, 69.4, 47.8, 36.1; HRMS (ESI-QTOF) *m*/*z* calcd for C_25_H_21_F_3_N_3_O_4_^+^ [M+H]^+^ 484.1479, found 484.1494.

(*S*)-*N*-(Furan-2-ylmethyl)-2-(4-trifluoromethoxybenzyl)-1-oxo-1,2,3,4-tetrahydropyrazino[1,2-*a*]indole-3-carboxamide (**1i**). Compound **1i** was synthesized according to the synthetic procedure given above, with a yield of 66%. ^1^H NMR (400 MHz, CDCl_3_) *δ* 7.72 (d, *J* = 8.0 Hz, 1H), 7.35–7.29 (m, 3H), 7.28 (s, 1H), 7.19 (ddd, *J* = 8.0, 5.8, 2.0 Hz, 1H), 7.07 (d, *J* = 8.5 Hz, 2H), 6.99 (d, *J* = 8.5 Hz, 2H), 6.41 (t, *J* = 5.8 Hz, 1H, NH), 6.30 (dd, *J* = 3.2, 1.9 Hz, 1H), 6.17 (d, *J* = 16.7 Hz, 1H), 6.13 (d, *J* = 3.2 Hz, 1H), 5.72 (d, *J* = 16.7 Hz, 1H), 4.86 (dd, *J* = 11.0, 8.5 Hz, 1H), 4.63 (dd, *J* = 11.0, 8.5 Hz, 1H), 4.52 (t, *J* = 8.5 Hz, 1H), 4.38 (dd, *J* = 15.6, 5.8 Hz, 1H), 4.13 (dd, *J* = 15.6, 5.8 Hz, 1H); ^13^C NMR (100 MHz, CDCl_3_) *δ* 171.4, 160.4, 150.9, 149.7 (*J_FC_* = 1.4 Hz), 142.3, 141.2, 139.5, 130.2, 126.6, 125.4, 125.3, 124.0, 122.6, 121.2, 120.5 (*J_FC_* = 257 Hz), 119.4, 118.5, 110.5, 110.3, 109.9, 107.4, 69.5, 69.4, 47.8, 36.1; HRMS (ESI-QTOF) *m*/*z* calcd for C_25_H_21_F_3_N_3_O_4_^+^ [M+H]^+^ 484.1479, found 484.1491.

(*S*)-*N*-(Furan-2-ylmethyl)-2-(3-methylbenzyl)-1-oxo-1,2,3,4-tetrahydropyrazino[1,2-*a*]indole-3-carboxamide (**1j**). Compound **1j** was synthesized according to the synthetic procedure given above, with a yield of 49%. ^1^H NMR (400 MHz, CDCl_3_) *δ* 7.73 (dd, *J* = 8.0, 1.0 Hz, 1H), 7.38–7.28 (m, 3H), 7.27 (s, 1H), 7.18 (ddd, *J* = 8.0, 6.4, 1.4 Hz, 1H), 7.09 (t, *J* = 7.6 Hz, 1H), 6.99 (d, *J* = 7.6 Hz, 1H), 6.77 (s, 1H), 6.71 (d, *J* = 7.6 Hz, 1H), 6.28 (dd, *J* = 3.2, 1.9 Hz, 1H), 6.26 (s, 1H, NH), 6.14 (d, *J* = 16.7 Hz, 1H), 6.06 (dd, *J* = 3.2, 0.9 Hz, 1H), 5.69 (d, *J* = 16.7 Hz, 1H), 4.84 (dd, *J* = 11.0, 8.3 Hz, 1H), 4.60 (dd, *J* = 11.0, 8.3 Hz, 1H), 4.50 (t, *J* = 8.3 Hz, 1H), 4.28 (dd, *J* = 15.6, 6.3 Hz, 1H), 4.02 (dd, *J* = 15.7, 6.3 Hz, 1H), 2.22 (s, 3H); ^13^C NMR (100 MHz, CDCl_3_) *δ*171.8, 160.4, 151.3, 142.0, 139.8, 138.8, 138.7, 128.7, 128.0, 126.5, 126.3, 125.4, 125.1, 122.7, 122.5, 120.9, 110.47, 110.46, 109.5, 107.1, 69.5, 69.4, 48.2, 36.2, 21.6; HRMS (ESI-QTOF) *m*/*z* calcd for C_25_H_24_N_3_O_3_^+^ [M+H]^+^ 414.1812, found 414.1818.

(*S*)-*N*-(Furan-2-ylmethyl)-2-(4-methylbenzyl)-1-oxo-1,2,3,4-tetrahydropyrazino[1,2-*a*]indole-3-carboxamide (**1k**). Compound **1k** was synthesized according to the synthetic procedure given above, with a yield of 45%. ^1^H NMR (400 MHz, CDCl_3_) *δ* 7.72 (dt, *J* = 8.0, 1.0 Hz, 1H), 7.38–7.28 (m, 3H), 7.26 (d, *J* = 0.8 Hz, 1H), 7.18 (ddd, *J* = 8.0, 6.7, 1.2 Hz, 1H), 7.01 (d, *J* = 8.0 Hz, 2H), 6.83 (d, *J* = 8.0 Hz, 2H), 6.34 (t, *J* = 5.8 Hz, 1H, NH), 6.29 (dd, *J* = 3.2, 1.8 Hz, 1H), 6.14 (d, *J* = 16.6 Hz, 1H), 6.06 (dd, *J* = 3.2, 0.9 Hz, 1H), 5.68 (d, *J* = 16.6 Hz, 1H), 4.84 (dd, *J* = 11.0, 8.0 Hz, 1H), 4.60 (dd, *J* = 11.0, 8.6 Hz, 1H), 4.49 (dd, *J* = 8.6, 8.0 Hz, 1H), 4.30 (dd, *J* = 15.7, 5.8 Hz, 1H), 4.02 (dd, *J* = 15.7, 5.8 Hz, 1H), 2.25 (s, 3H); ^13^C NMR (100 MHz, CDCl_3_) *δ* 171.8, 160.4, 151.3, 142.0, 139.8, 136.75, 135.72, 129.6, 126.5, 125.7, 125.3, 125.1, 122.5, 120.9, 110.48, 110.46, 109.5, 107.1, 69.5, 69.4, 48.0, 36.1, 21.1; HRMS (ESI-QTOF) *m*/*z* calcd for C_25_H_24_N_3_O_3_^+^ [M+H]^+^ 414.1812, found 414.1817.

(*S*)-*N*-(Furan-2-ylmethyl)-2-(naphthalen-1-ylmethyl)-1-oxo-1,2,3,4-tetrahydropyrazino[1,2-*a*]indole-3-carboxamide (**1l**). Compound **1l** was synthesized according to the synthetic procedure given above, with a yield of 48%. ^1^H NMR (400 MHz, CDCl_3_) *δ* 7.79–7.74 (m, 2H), 7.72 (d, *J* = 8.5 Hz, 1H), 7.65–7.60 (m, 1H), 7.45–7.35 (m, 3H), 7.34–7.28 (m, 2H), 7.25 (s, 1H), 7.23–7.17 (m, 2H), 7.16 (dd, *J* = 1.9, 0.9 Hz, 1H), 6.36 (d, *J* = 16.9 Hz, 1H), 6.16 (dd, *J* = 3.2, 1.9 Hz, 1H), 6.13 (d, *J* = 6.3 Hz, 1H, NH), 5.86 (d, *J* = 16.9 Hz, 1H), 5.81 (dd, *J* = 3.2, 0.9 Hz, 1H), 4.82 (dd, *J* = 10.9, 7.9 Hz, 1H), 4.59 (dd, *J* = 10.9, 8.7 Hz, 1H), 4.48 (dd, *J* = 8.7, 7.9 Hz, 1H), 3.98 (dd, *J* = 15.6, 6.3 Hz, 1H), 3.55 (dd, *J* = 15.6, 6.3 Hz, 1H); ^13^C NMR (100 MHz, CDCl_3_) *δ* 171.6, 160.4, 151.0, 142.0, 139.8, 136.3, 133.4, 132.6, 128.8, 127.9, 127.8, 126.6, 126.5, 126.0, 125.5, 125.2, 124.1, 124.0, 122.6, 121.0, 110.5, 110.3, 109.7, 106.9, 69.5, 69.4, 48.4, 35.8; HRMS (ESI-QTOF) *m*/*z* calcd for C_28_H_24_N_3_O_3_^+^ [M+H]^+^ 450.1812, found 450.1818.

(*S*)-*N*-(Furan-2-ylmethyl)-2-([1,1′-biphenyl]-4-ylmethyl)-1-oxo-1,2,3,4-tetrahydropyrazino[1,2-*a*]indole-3-carboxamide (**1m**). Compound **1m** was synthesized according to the synthetic procedure given above, with a yield of 69%. ^1^H NMR (400 MHz, CDCl_3_) *δ* 7.74 (dt, *J* = 8.0, 1.0 Hz, 1H), 7.50–7.47 (m, 2H), 7.47–7.43 (m, 2H), 7.43–7.39 (m, 2H), 7.38–7.36 (m, 1H), 7.35–7.30 (m, 2H), 7.29 (d, *J* = 0.8 Hz, 1H), 7.22 (dd, *J* = 1.9, 0.8 Hz, 1H), 7.24–7.14 (m, 1H), 7.02 (d, *J* = 8.5 Hz, 2H), 6.40 (t, *J* = 6.1 Hz, 1H, NH), 6.24 (d, *J* = 16.8 Hz, 1H), 6.19 (dd, *J* = 3.2, 1.9 Hz, 1H), 5.95 (dd, *J* = 3.2, 0.9 Hz, 1H), 5.76 (d, *J* = 16.8 Hz, 1H), 4.87 (dd, *J* = 11.0, 8.0 Hz, 1H), 4.62 (dd, *J* = 11.0, 8.7 Hz, 1H), 4.51 (dd, *J* = 8.7, 8.0 Hz, 1H), 4.28 (dd, *J* = 15.6, 6.1 Hz, 1H), 3.97 (dd, *J* = 15.6, 6.1 Hz, 1H); ^13^C NMR (100 MHz, CDCl_3_) *δ* 171.7, 160.5, 142.1, 140.5, 140.0, 139.7, 129.0, 128.9, 127.6, 127.5, 127.1, 127.0, 126.6, 126.2, 125.4, 125.2, 122.5, 121.0, 110.5, 110.4, 109.7, 107.2, 69.5, 69.4, 48.0, 36.1; HRMS (ESI-QTOF) *m*/*z* calcd for C_30_H_26_N_3_O_3_^+^ [M+H]^+^ 476.1969, found 476.1977.

#### 4.1.3. General Procedure of *N*-Benzyl (*S*)-2-Arylmethyl-1-oxo-1,2,3,4-tetrahydropyrazino[1,2-*a*]indol-3-carboxamide (**2a**–**m**)

To a solution of (*S*)-methyl 2-arylmethyl-1-oxo-1,2,3,4-tetrahydropyrazino[1,2-*a*]-indole-3-carboxylate (**7a**–**m**) (1.00 equiv.) in absolute methanol, benzylamine (5.00 equiv.) was added and stirred for 15h at r.t. After evaporation, the residue was purified by column chromatography (EtOAc/hexanes = 1:2).

(*S*)-*N*-Benzyl-2-(3-nitrobenzyl)-1-oxo-1,2,3,4-tetrahydropyrazino[1,2-*a*]indole-3-carboxamide (**2a**). Compound **2a** was synthesized according to the synthetic procedure given above, with a yield of 73%. ^1^H NMR (400 MHz, CDCl_3_) *δ* 7.91 (s, 1H), 7.86 (dt, *J* = 7.1, 2.3 Hz, 1H), 7.74 (d, *J* = 7.9 Hz, 1H), 7.34–7.26 (m, 5H), 7.25–7.19 (m, 2H), 7.18–7.11 (m, 2H), 7.09–7.04 (m, 2H), 6.33 (t, *J* = 6.3 Hz, 1H, NH), 6.19, 5.79 (ABq, *J* = 16.9 Hz, 2H), 4.90 (dd, *J* = 11.0, 8.4 Hz, 1H), 4.68 (dd, *J* = 11.0, 8.8 Hz, 1H), 4.57 (t, *J* = 8.6 Hz, 1H), 4.38 (dd, *J* = 15.0, 6.4 Hz, 1H), 4.20 (dd, *J* = 15.0, 5.7 Hz, 1H); ^13^C NMR (100 MHz, CDCl_3_) *δ* 171.4, 160.5, 148.5, 140.8, 139.4, 137.7, 131.8, 129.7, 128.8, 127.7, 127.5, 126.8, 125.6, 125.3, 122.8, 122.4, 121.4, 121.0, 110.3, 110.1, 69.59, 69.57, 47.7, 43.1; HRMS (ESI-QTOF) *m*/*z* calcd for C_26_H_23_N_4_O_4_^+^ [M+H]^+^ 455.1714, found 455.1719.

(*S*)-*N*-Benzyl-2-(4-nitrobenzyl)-1-oxo-1,2,3,4-tetrahydropyrazino[1,2-*a*]indole-3-carboxamide (**2b**). Compound **2b** was synthesized according to the synthetic procedure given above, with a yield of 52%. ^1^H NMR (400 MHz, CDCl_3_) *δ* 7.89 (d, *J* = 8.8 Hz, 2H), 7.76–7.72 (m, 1H), 7.33–7.26 (m, 5H), 7.23–7.18 (m, 2H), 7.07–7.03 (m, 2H), 7.00 (d, *J* = 8.8 Hz, 2H), 6.30–6.23 (m, 2H), 5.75 (d, *J* = 17.3 Hz, 1H), 4.89 (dd, *J* = 11.0, 8.4 Hz, 1H), 4.67 (dd, *J* = 11.0, 8.8 Hz, 1H), 4.56 (t, *J* = 8.6 Hz, 1H), 4.36 (dd, *J* = 15.0, 6.4 Hz, 1H), 4.16 (dd, *J* = 15.0, 5.7 Hz, 1H); ^13^C NMR (100 MHz, CDCl_3_) *δ* 171.4, 160.4, 147.1, 146.0, 139.4, 137.6, 128.9, 127.8, 127.3, 126.7, 126.5, 125.6, 125.2, 124.1, 122.8, 121.5, 110.2, 110.1, 69.58, 69.54, 47.8, 43.1; HRMS (ESI-QTOF) *m*/*z* calcd for C_26_H_23_N_4_O_4_^+^ [M+H]^+^ 455.1714, found 455.1717.

(*S*)-*N*-Benzyl-2-(3-fluorobenzyl)-1-oxo-1,2,3,4-tetrahydropyrazino[1,2-*a*]indole-3-carboxamide (**2c**). Compound **2c** was synthesized according to the synthetic procedure given above, with a yield of 44%. ^1^H NMR (400 MHz, CDCl_3_) *δ* 7.72 (d, *J* = 8.0 Hz, 1H), 7.32–7.24 (m, 6H), 7.18 (ddd, *J* = 7.9, 5.6, 2.3 Hz, 1H), 7.12–7.08 (m, 2H), 7.00 (td, *J* = 7.9, 5.8 Hz, 1H), 6.74 (td, *J* = 8.2, 2.3 Hz, 1H), 6.69 (d, *J* = 7.7 Hz, 1H), 6.59 (d, *J* = 9.2 Hz, 1H), 6.41 (t, *J* = 6.4 Hz, 1H, NH), 6.15, 5.69 (ABq, *J* = 16.8 Hz, 2H), 4.88 (dd, *J* = 10.9, 8.1 Hz, 1H), 4.64 (dd, *J* = 10.9, 8.7 Hz, 1H), 4.52 (dd, *J* = 8.7, 8.1 Hz, 1H), 4.37 (dd, *J* = 15.0, 6.7 Hz, 1H), 4.12 (dd, *J* = 15.0, 5.8 Hz, 1H); ^13^C NMR (100 MHz, CDCl_3_) *δ* 171.7, 163.2 (*J_FC_* = 247 Hz), 160.3, 141.5 (*J_FC_* = 6.9 Hz), 139.5, 137.9, 130.3 (*J_FC_* = 8.2 Hz), 128.7, 127.5, 127.4, 126.6, 125.3 (*J_FC_* = 2.4 Hz), 122.6, 121.2 (*J_FC_* = 2.6 Hz), 121.1, 114.1 (*J_FC_* = 21.1 Hz), 112.8 (*J_FC_* = 22.1 Hz), 110.2, 109.8, 69.5, 69.5, 47.7, 42.9; HRMS (ESI-QTOF) *m*/*z* calcd for C_26_H_23_FN_3_O_2_^+^ [M+H]^+^ 428.1769, found 428.1775.

(*S*)-*N*-Benzyl-2-(4-fluorobenzyl)-1-oxo-1,2,3,4-tetrahydropyrazino[1,2-*a*]indole-3-carboxamide (**2d**). Compound **2d** was synthesized according to the synthetic procedure given above, with a yield of 67%. ^1^H NMR (400 MHz, CDCl_3_) *δ* 7.72 (dt, *J* = 8.0, 1.0 Hz, 1H), 7.33–7.26 (m, 6H), 7.17 (ddd, *J* = 8.0, 5.2, 2.8 Hz, 1H), 7.14–7.10 (m, 2H), 6.87–6.81 (m, 2H), 6.77–6.69 (m, 2H), 6.33 (d, *J* = 6.5 Hz, 1H, NH), 6.13, 5.62 (ABq, *J* = 16.6 Hz, 2H), 4.89 (dd, *J* = 10.9, 8.1 Hz, 1H), 4.64 (dd, *J* = 10.9, 8.7 Hz, 1H), 4.53 (dd, *J* = 8.7, 8.1 Hz, 1H), 4.38 (dd, *J* = 15.0, 6.7 Hz, 1H), 4.11 (dd, *J* = 15.0, 5.7 Hz, 1H); ^13^C NMR (100 MHz, CDCl_3_) *δ* 171.7, 161.8 (*J_FC_* = 245 Hz), 160.4, 139.6, 137.9 134.3 (*J_FC_* = 3.1 Hz), 128.8, 127.6, 127.5, 127.3 (*J_FC_* = 7.9 Hz), 126.6, 125.3, 125.2, 122.6, 121.1, 115.7 (*J_FC_* = 21.5 Hz), 110.3, 109.8, 69.6, 69.5, 47.5, 43.0; HRMS (ESI-QTOF) *m*/*z* calcd for C_26_H_23_FN_3_O_2_^+^ [M+H]^+^ 428.1769, found 428.1778.

(*S*)-*N*-Benzyl-2-(3-cyanobenzyl)-1-oxo-1,2,3,4-tetrahydropyrazino[1,2-*a*]indole-3-carboxamide (**2e**). Compound **2e** was synthesized according to the synthetic procedure given above, with a yield of 67%. ^1^H NMR (400 MHz, CDCl_3_) *δ* 7.74 (d, *J* = 7.9 Hz, 1H), 7.35–7.26 (m, 6H), 7.24–7.17 (m, 3H), 7.13–7.06 (m, 4H), 6.29 (t, *J* = 5.8 Hz, 1H, NH), 6.10, 5.75 (ABq, *J* = 16.9 Hz, 2H), 4.89 (dd, *J* = 11.0, 8.4 Hz, 1H), 4.66 (dd, *J* = 11.0, 8.7 Hz, 1H), 4.56 (t, *J* = 8.5 Hz, 1H), 4.41 (dd, *J* = 15.1, 6.4 Hz, 1H), 4.20 (dd, *J* = 15.0, 5.7 Hz, 1H); ^13^C NMR (100 MHz, CDCl_3_) *δ* 171.4, 160.4, 140.2, 139.3, 137.7, 130.9, 130.1, 129.5, 129.2, 128.9, 127.7, 127.5, 126.7, 125.5, 125.1, 122.8, 121.4, 118.6, 112.9, 110.2, 110.0, 69.52, 69.50, 47.5, 43.1; HRMS (ESI-QTOF) *m*/*z* calcd for C_27_H_23_N_4_O_2_^+^ [M+H]^+^ 435.1816, found 435.1823.

(*S*)-*N*-Benzyl-2-(4-cyanobenzyl)-1-oxo-1,2,3,4-tetrahydropyrazino[1,2-*a*]indole-3-carboxamide (**2f**). Compound **2f** was synthesized according to the synthetic procedure given above, with a yield of 70%. ^1^H NMR (400 MHz, CDCl_3_) *δ* 7.73 (d, *J* = 7.9 Hz, 1H), 7.37–7.27 (m, 5H), 7.24 (d, *J* = 8.6 Hz, 2H), 7.22–7.17 (m, 2H), 7.13 (dd, *J* = 7.7, 1.8 Hz, 2H), 6.93 (d, *J* = 8.3 Hz, 2H), 6.25 (t, *J* = 6.1 Hz, 1H, NH), 6.20, 5.69 (ABq, *J* = 17.2 Hz, 2H), 4.88 (dd, *J* = 10.9, 8.3 Hz, 1H), 4.65 (dd, *J* = 11.0, 8.8 Hz, 1H), 4.55 (t, *J* = 8.5 Hz, 1H), 4.39 (dd, *J* = 15.0, 6.4 Hz, 1H), 4.15 (dd, *J* = 15.0, 5.6 Hz, 1H); ^13^C NMR (100 MHz, CDCl_3_) *δ* 171.4, 160.4, 144.1, 139.4, 137.7, 132.6, 129.0, 127.8, 127.5, 126.6, 126.4, 125.5, 125.2, 122.7, 121.4, 118.6, 111.1, 110.1, 110.0, 69.52, 69.49, 47.9, 43.1; HRMS (ESI-QTOF) *m*/*z* calcd for C_27_H_23_N_4_O_2_^+^ [M+H]^+^ 435.1816, found 435.1821.

(*S*)-*N*-Benzyl-2-(4-trifluoromethylbenzyl)-1-oxo-1,2,3,4-tetrahydropyrazino[1,2-*a*]indole-3-carboxamide (**2g**). Compound **2g** was synthesized according to the synthetic procedure given above, with a yield of 72%. ^1^H NMR (400 MHz, CDCl_3_) *δ* 7.74 (d, *J* = 8.0 Hz, 1H), 7.35 (d, *J* = 8.1 Hz, 2H), 7.33–7.26 (m, 5H), 7.26–7.23 (m, 1H), 7.19 (ddd, *J* = 7.9, 6.7, 1.1 Hz, 1H), 7.14–7.10 (m, 2H), 7.00 (d, *J* = 8.0 Hz, 2H), 6.38 (t, *J* = 6.3 Hz, 1H, NH), 6.24, 5.73 (ABq, *J* = 16.9 Hz, 2H), 4.89 (dd, *J* = 11.0, 8.3 Hz, 1H), 4.65 (dd, *J* = 11.0, 8.7 Hz, 1H), 4.55 (t, *J* = 8.5 Hz, 1H), 4.40 (dd, *J* = 15.1, 6.8 Hz, 1H), 4.07 (dd, *J* = 15.1, 5.6 Hz, 1H); ^13^C NMR (100 MHz, CDCl_3_) *δ* 171.5, 160.4, 142.8 (*J_FC_* = 1.3 Hz), 139.5, 137.9, 129.5 (*J_FC_* = 32.5 Hz), 128.8, 127.6, 127.4, 126.7, 126.2, 125.90 (*J_FC_* = 3.8 Hz), 125.4, 125.3, 124.1 (*J_FC_* = 272 Hz), 122.7, 121.3, 110.2, 110.0, 69.6, 69.5, 47.9, 42.9; HRMS (ESI-QTOF) *m*/*z* calcd for C_27_H_23_F_3_N_3_O_2_^+^ [M+H]^+^ 478.1737, found 478.1746.

(*S*)-*N*-Benzyl-2-(3-trifluoromethoxybenzyl)-1-oxo-1,2,3,4-tetrahydropyrazino[1,2-*a*]indole-3-carboxamide (**2h**). Compound **2h** was synthesized according to the synthetic procedure given above, with a yield of 85%. ^1^H NMR (400 MHz, CDCl_3_) *δ* 7.73 (d, *J* = 8.0 Hz, 1H), 7.34–7.26 (m, 6H), 7.19 (ddd, *J* = 8.0, 6.5, 1.4 Hz, 1H), 7.12–7.08 (m, 2H), 7.01 (t, *J* = 7.9 Hz, 1H), 6.92 (d, *J* = 8.2 Hz, 1H), 6.84 (s, 1H), 6.76 (d, *J* = 7.6 Hz, 1H), 6.36 (t, *J* = 5.7 Hz, 1H, NH), 6.13, 5.72 (ABq, *J* = 16.8 Hz, 2H), 4.88 (dd, *J* = 11.0, 8.3 Hz, 1H), 4.65 (dd, *J* = 11.0, 8.7 Hz, 1H), 4.54 (t, *J* = 8.5 Hz, 1H), 4.37 (dd, *J* = 15.1, 6.6 Hz, 1H), 4.15 (dd, *J* = 15.1, 5.8 Hz, 1H); ^13^C NMR (100 MHz, CDCl_3_) *δ* 171.6, 160.4, 149.7 (*J_FC_* = 1.7 Hz), 141.2, 139.5, 137.9, 130.2, 128.8 127.6, 127.5, 126.6, 125.4, 125.3, 123.9, 122.6, 121.2, 120.5 (*J_FC_* = 258 Hz), 119.3 (*J_FC_* = 0.8 Hz), 118.4 (*J_FC_* = 0.8 Hz), 110.2, 109.9, 69.6, 69.5, 47.8, 43.0; HRMS (ESI-QTOF) *m*/*z* calcd for C_27_H_23_F_3_N_3_O_3_^+^ [M+H]^+^ 494.1686, found 494.1699.

(*S*)-*N*-Benzyl-2-(4-trifluoromethoxybenzyl)-1-oxo-1,2,3,4-tetrahydropyrazino[1,2-*a*]indole-3-carboxamide (**2i**). Compound **2i** was synthesized according to the synthetic procedure given above, with a yield of 70%. ^1^H NMR (400 MHz, CDCl_3_) *δ* 7.72 (d, *J* = 8.0 Hz, 1H), 7.34–7.25 (m, 6H), 7.18 (t, *J* = 7.0 Hz, 1H), 7.14 (d, *J* = 6.9 Hz, 2H), 6.91 (m, 4H), 6.42 (t, *J* = 6.5 Hz, 1H, NH), 6.15, 5.68 (ABq, *J* = 16.7 Hz, 2H), 4.90 (dd, *J* = 10.9, 8.2 Hz, 1H), 4.65 (dd, *J* = 10.9, 8.7 Hz, 1H), 4.54 (t, *J* = 8.5 Hz, 1H), 4.41 (dd, *J* = 15.0, 6.7 Hz, 1H), 4.10 (dd, *J* = 15.0, 5.6 Hz, 1H); ^13^C NMR (100 MHz, CDCl_3_) *δ* 171.6, 160.4, 148.2 (*J_FC_* = 1.8 Hz), 139.5, 137.9, 137.3, 128.8, 127.6, 127.5, 127.2, 126.6, 125.3, 125.2, 122.61, 121.3 (*J_FC_* = 1.0 Hz), 121.2, 120.5 (*J_FC_* = 257 Hz), 110.3, 109.9, 69.6, 69.2, 47.5, 43.0; HRMS (ESI-QTOF) *m*/*z* calcd for C_27_H_23_F_3_N_3_O_3_^+^ [M+H]^+^ 494.1686, found 494.1695.

(*S*)-*N*-Benzyl-2-(3-methylbenzyl)-1-oxo-1,2,3,4-tetrahydropyrazino[1,2-*a*]indole-3-carboxamide (**2j**). Compound **2j** was synthesized according to the synthetic procedure given above, with a yield of 28%. ^1^H NMR (400 MHz, CDCl_3_) *δ* 7.73 (dt, *J* = 8.0, 1.0 Hz, 1H), 7.33–7.23 (m, 6H), 7.18 (ddd, *J* = 8.0, 6.2, 1.8 Hz, 1H), 7.09–7.04 (m, 2H), 6.97 (t, *J* = 7.6 Hz, 1H), 6.88 (d, *J* = 7.6 Hz, 1H), 6.73 (s, 1H), 6.66 (d, *J* = 7.6 Hz, 1H), 6.29 (t, *J* = 6.3 Hz, 1H, NH), 6.16, 5.65 (ABq, *J* = 16.7 Hz, 2H), 4.87 (dd, *J* = 10.9, 7.8 Hz, 1H), 4.62 (dd, *J* = 10.9, 8.7 Hz, 1H), 4.52 (dd, *J* = 8.7, 7.8 Hz, 1H), 4.30 (dd, *J* = 15.1, 6.9 Hz, 1H), 4.03 (dd, *J* = 15.1, 5.9 Hz, 1H), 2.11 (s, 3H); ^13^C NMR (100 MHz, CDCl_3_) *δ* 172.0, 160.4, 139.8, 138.8, 138.6, 138.2, 128.68, 128.65, 127.9, 127.33, 127.26, 126.5, 126.2, 125.4, 125.1, 122.6, 122.5, 120.9, 110.4, 109.5, 69.6, 69.5, 48.2, 42.9, 21.5; HRMS (ESI-QTOF) *m*/*z* calcd for C_27_H_26_N_3_O_2_^+^ [M+H]^+^ 424.2020, found 424.2026.

(*S*)-*N*-Benzyl-2-(4-methylbenzyl)-1-oxo-1,2,3,4-tetrahydropyrazino[1,2-*a*]indole-3-carboxamide (**2k**). Compound **2k** was synthesized according to the synthetic procedure given above, with a yield of 45%. ^1^H NMR (400 MHz, CDCl_3_) *δ* 7.72 (d, *J* = 8.1 Hz, 1H), 7.35–7.24 (m, 6H), 7.17 (ddd, *J* = 7.9, 6.5, 1.3 Hz, 1H), 7.09–7.06 (m, 2H), 6.89 (d, *J* = 7.7 Hz, 2H), 6.79 (d, *J* = 7.9 Hz, 2H), 6.39 (t, *J* = 6.4 Hz, 1H, NH), 6.17 (d, *J* = 16.5 Hz, 1H), 5.64 (d, *J* = 16.5 Hz, 1H), 4.88 (dd, *J* = 11.0, 7.9 Hz, 1H), 4.62 (dd, *J* = 11.0, 8.7 Hz, 1H), 4.51 (dd, *J* = 8.7, 7.9 Hz, 1H), 4.33 (dd, *J* = 15.2, 6.8 Hz, 1H), 4.04 (dd, *J* = 15.2, 5.9 Hz, 1H), 2.16 (s, 3H); ^13^C NMR (100 MHz, CDCl_3_) *δ* 171.9, 160.4, 139.7, 138.1, 136.7, 135.7, 129.6, 128.7, 127.4, 127.2, 126.5, 125.6, 125.4, 125.1, 122.5, 120.9, 110.5, 109.5, 69.55, 69.56, 47.9, 42.8, 21.1; HRMS (ESI-QTOF) *m*/*z* calcd for C_27_H_26_N_3_O_2_^+^ [M+H]^+^ 424.2020, found 424.2021.

(*S*)-*N*-Benzyl-2-(naphthalen-1-ylmethyl)-1-oxo-1,2,3,4-tetrahydropyrazino[1,2-*a*]indole-3-carboxamide (**2l**). Compound **2l** was synthesized according to the synthetic procedure given above, with a yield of 88%. ^1^H NMR (400 MHz, CDCl_3_) *δ* 7.77 (d, *J* = 8.0 Hz, 1H), 7.70 (dd, *J* = 6.3, 2.8 Hz, 1H), 7.60 (d, *J* = 6.7 Hz, 1H), 7.57 (d, *J* = 9.0 Hz, 1H), 7.42–7.38 (m, 2H), 7.38–7.35 (m, 1H), 7.33 (s, 1H), 7.31 (ddd, *J* = 8.4, 6.9, 1.2 Hz, 1H), 7.22 (d, *J* = 2.0 Hz, 1H), 7.21–7.13 (m, 5H), 6.83–6.79 (m, 2H), 6.38 (d, *J* = 16.8 Hz, 1H), 6.16 (t, *J* = 6.2 Hz, 1H, NH), 5.82 (d, *J* = 16.8 Hz, 1H), 4.85 (dd, *J* = 10.9, 7.9 Hz, 1H), 4.61 (dd, *J* = 10.9, 8.6 Hz, 1H), 4.51 (dd, *J* = 8.6, 7.9 Hz, 1H), 4.00 (dd, *J* = 15.0, 7.0 Hz, 1H), 3.50 (dd, *J* = 15.0, 5.7 Hz, 1H); ^13^C NMR (100 MHz, CDCl_3_) *δ* 171.8, 160.4, 139.8, 137.9, 136.3, 133.4, 132.5, 128.7, 128.5, 127.8, 127.3, 127.21, 128.20, 126.60, 126.55, 126.0, 125.4, 125.2, 124.0, 123.9, 122.6, 121.0, 110.4, 109.6, 69.52, 69.49, 48.4, 42.6; HRMS (ESI-QTOF) *m*/*z* calcd for C_30_H_26_N_3_O_2_^+^ [M+H]^+^ 460.2020, found 460.2022.

(*S*)-*N*-Benzyl-2-([1,1′-biphenyl]-4-ylmethyl)-1-oxo-1,2,3,4-tetrahydropyrazino[1,2-*a*]indole-3-carboxamide (**2m**). Compound **2m** was synthesized according to the synthetic procedure given above, with a yield of 50%. ^1^H NMR (400 MHz, CDCl_3_) *δ* 7.75 (d, *J* = 8.2 Hz, 1H), 7.39 (d, *J* = 4.4 Hz, 4H), 7.37–7.33 (m, 2H), 7.33–7.30 (m, 4H), 7.23–7.16 (m, 4H), 6.97 (d, *J* = 7.9 Hz, 2H), 6.96–6.93 (m, 2H), 6.39 (t, *J* = 6.4 Hz, 1H, NH), 6.26 (d, *J* = 16.7 Hz, 1H), 5.71 (d, *J* = 16.7 Hz, 1H), 4.91 (dd, *J* = 10.9, 8.0 Hz, 1H), 4.64 (dd, *J* = 10.9, 8.8 Hz, 1H), 4.54 (t, *J* = 8.4 Hz, 1H), 4.29 (dd, *J* = 15.2, 6.9 Hz, 1H), 3.94 (dd, *J* = 15.2, 5.7 Hz, 1H); ^13^C NMR (100 MHz, CDCl_3_) *δ* 171.9, 160.4, 140.3, 140.0, 139.7, 138.0, 137.8, 128.9, 128.6, 127.54, 127.49, 127.3, 127.1, 127.0, 126.6, 126.2, 125.4, 125.2, 122.6, 121.0, 110.4, 109.7, 69.56, 69.57, 48.0, 42.8; HRMS (ESI-QTOF) *m*/*z* calcd for C_32_H_28_N_3_O_2_^+^ [M+H]^+^ 486.2176, found 486.2179.

### 4.2. Cell Culture

Human breast cancer cell lines, MCF-7 and MDA-MB-468, were purchased from American Type Culture Collection (ATCC, Manassas, VA, USA). Cells were cultured in RPMI1640 (Capricorn scientific, Ebsdorfergrund, Germany) containing 10% fetal bovine serum (FBS, Hyclone, Logan, UT, USA), 100 U/mL penicillin and 100 μg/mL streptomycin (GenDEPOT, Barker, TX, USB) at 37 °C in a humidified incubator with 5% CO_2_.

### 4.3. Cell Viability Assays

Cells were plated into 96 well plates at a density of 7 × 10^3^ (MCF-7), 9 × 10^3^ (MDA-MB-468) cells per well and incubated for 24 h at 37 °C in a CO_2_ incubator. Furthermore, cells were treated with various concentrations of each compound that contained gefitinib (MedKoo Biosciences, CHAPEL Hill, NC, USA) and incubated for 48 h. To determine cell viability, 3-(4,5-dimethylthiazol)-2,5-diphenyltetrazolium bromide (MTT, USB, Cleveland, OH, USA) solution was added to each well and incubated 37 °C for 1 h. After cells were dissolved in dimethyl sulfoxide (DMSO), absorbance per well was measured at 540 nm using the MULTISKAN GO reader (Thermo Scientific, Waltham, MA, USA). The results are expressed as a percentage (%) of the control cell viability.

### 4.4. Protein Extraction and Western Blotting Analysis

Cells were plated in 6 well plate and treated with 10 µM of each compound. The cells were lysed with ice-cold RIPA buffer (Thermo Scientific, Waltham, MA, USA) and protein concentration was quantified using the BCA reagents (Thermo scientific, Sunnyvale, CA, USA). Equal amounts of protein samples were separated by SDS-PAGE using 10% polyacrylamide gel and then transferred onto a polyvinylidene difluoride (PVDF) membrane (Millipore, Billerica, MA, USA). The membranes were incubated with primary antibodies (dilution 1:5000): anti-phospho-ERK, anti-ERK, anti-phospho-Akt(Ser473), anti-phospho-Akt(Thr308), anti-Akt (Cell signaling, Danvers, MA, USA); anti-GAPDH (Santa Cruz Biotechonology, Dallas, TX, USA). The antigens and antibodies were detected using a Western Bright ECL HRP substrate kit (Advansta, Menlo Park, CA, USA).

### 4.5. Flow Cytometric Analysis with Annexin V and Propidium Iodide

Apoptotic and live cells were determined by fluorescence-activated cell sorting (FACS) analysis according to the instructions provided with the annexin V-FITC Apoptosis Detection Kit (BD Biosciences, Bedford, MA, USA). Gefitinib and compounds **2b**, **2f** and **2i** were treated for 24 h and harvested, trypsinized, washed with cold PBS, and resuspended in 1X binding buffer. The stained cells by annexin V-FITC and propidium iodide (PI) were analyzed using the Becton Dickinson FACSscan flow cytometer (BD Biosciences, San Jose, CA, USA) and BD FACSDiva software (BD Biosciences, San Jose, CA, USA).

### 4.6. Kinase Assay

In vitro assay of the 18 kinases was performed at Reaction Biology Corporation. Kinase and substrate pairs, along with the required cofactors, were prepared in base reaction buffer: 20 mM Hepes pH 7.5, 10 mM MgCl_2_, 1 mM EGTA, 0.02% Brij35, 0.02 mg/mL BSA, 0.1 mM Na_3_VO_4_, 2 mM DTT, 1% DMSO. Compounds in 100% DMSO were delivered into the kinase reaction mixture by Acoustic technology (Labcyte^®^ Echo550; nanoliter range), followed 20 min later by the addition of a mixture of ^33^P-ATP (PerkinElmer, Shelton, CT, USA; 10 µCi/µL) to a concentration of 10 µM. Reactions were carried out at 25 °C for 120 min, followed by spotting of the reactions onto Whatman^®^ P81 ion exchange filter paper. Unbound phosphate was removed by extensive washing of filters in 0.75% phosphoric acid. After the subtraction of background derived from the control reactions containing inactive enzyme, kinase activity data were expressed as the percentages of remaining kinase activity in test samples compared to vehicle (DMSO) reactions.

## 5. Patents

“Pyrazinoindolone derivatives”. Korean patent, KR2271065, June 2021.

## Figures and Tables

**Table 1:** Cell viability at 3 and 30 µM and GI_50_ value of 2-(substituted arylmetyl)-1-oxo-1,2,3,4-tetrahydropyrazino[1,2-*a*]indole-3-carboxamide analogs (series **1** and **2**) against the MCF-7 and MDA-MB-468 cell lines.

Analogs	% Cell Viability at 3 µM		% Cell Viability at 30 µM		GI_50_ Value ^a^ (µM)		
	MCF-7	MDA-MB-468	MCF-7	MDA-MB-468	MCF-7	MDA-MB-468	Ratio
Ctrl	100 ± 3.3	100 ± 2.6	100 ± 3.4	100 ± 2.0	-	-	-
Ge	82.2 ± 4.0	62.7 ± 1.9	69.8 ± 4.2	16.4 ± 0.9	>30	13	>2.31
**1a**	77.4 ± 3.6	84.9 ± 4.8	68.0 ± 4.0	39.1 ± 0.9	>30	19.8	>1.52
**1b**	87.2 ± 3.1	83.9 ± 3.5	84.4 ± 4.3	39.8 ± 1.9	>30	17.3	>1.73
**1c**	91.4 ± 10.8	83.7 ± 5.2	63.3 ± 3.6	27.1 ± 0.5	>30	8.03	>3.74
**1d**	93.6 ± 3.6	79.8 ± 2.5	66.7 ± 3.6	30.3 ± 2.0	>30	8.75	>3.43
**1e**	91.5 ± 5.7	84.2 ± 4.0	76.9 ± 2.2	32.0 ± 1.8	>30	19.9	>1.51
**1f**	82.7 ± 4.6	76.2 ± 3.8	80.0 ± 3.0	39.7 ± 1.1	>30	19.1	>1.57
**1g**	89.5 ± 7.9	79.8 ± 3.7	56.5 ± 3.5	27.6 ± 2.2	>30	8.18	>3.67
**1h**	90.3 ± 4.3	74.9 ± 0.7	75.9 ± 4.1	31.5 ± 2.1	>30	7.95	>3.77
**1i**	76.7 ± 7.3	60.9 ± 0.9	84.3 ± 1.5	41.7 ± 1.5	>30	8.34	>3.60
**1j**	87.1 ± 2.4	77.0 ± 2.5	40.1 ± 1.3	16.5 ± 0.5	12.2	6.57	1.86
**1k**	86.8 ± 6.2	83.9 ± 3.9	74.8 ± 5.5	41.3 ± 1.4	>30	9.33	>3.22
**1l**	88.5 ± 4.9	80.7 ± 1.5	59.9 ± 2.3	38.5 ± 1.0	>30	9.52	>3.15
**1m**	94.3 ± 7.7	89.0 ± 1.6	88.4 ± 3.2	66.9 ± 3.1	>30	>30	-
**2a**	89.8 ± 3.2	79.2 ± 7.5	72.0 ± 4.3	23.4 ± 1.9	>30	7.58	>3.96
**2b**	72.9 ± 6.6	50.9 ± 2.2	67.4 ± 7.0	34.7 ± 2.3	>30	4.03	>7.44
**2c**	72.5 ± 2.2	81.6 ± 1.5	41.4 ± 0.6	31.3 ± 1.6	12.5	7.85	1.59
**2d**	75.3 ± 9.5	86.1 ± 2.4	60.1 ± 3.9	34.8 ± 1.1	>30	8.8	>3.41
**2e**	75.3 ± 3.0	86.5 ± 6.8	63.4 ± 3.1	32.2 ± 5.6	>30	8.39	>3.58
**2f**	73.1 ± 5.2	48.8 ± 3.0	63.8 ± 6.9	28.0 ± 1.7	>30	2.94	>10.2
**2g**	72.2 ± 3.4	59.5 ± 1.8	55.0 ± 2.8	27.7 ± 2.0	>30	5.89	>5.09
**2h**	66.7 ± 2.0	61.8 ± 2.0	55.3 ± 5.5	30.9 ± 0.9	>30	7.05	>4.26
**2i**	68.4 ± 1.5	49.3 ± 2.6	61.3 ± 3.6	35.3 ± 0.9	>30	2.96	>10.1
**2j**	73.3 ± 4.1	79.5 ± 2.7	31.0 ± 0.5	21.4 ± 0.6	8.79	8.25	1.07
**2k**	70.7 ± 3.0	75.5 ± 4.8	49.3 ± 0.5	45.2 ± 0.5	21.4	23.1	0.93
**2l**	62.8 ± 6.2	66.4 ± 1.7	47.2 ± 1.6	46.7 ± 1.3	9.46	10.4	0.91
**2m**	69.4 ± 3.7	74.9 ± 2.2	55.2 ± 1.3	52.4 ± 0.9	>30	>30	-
Ref 1	82.9 ± 5.1	73.7 ± 3.4	40.3 ± 0.9	21.1 ± 3.7	19.3	5.2	3.71
Ref 2	97.3 ± 5.9	91.6 ± 3.0	30.7 ± 1.7	20.7 ± 0.8	9.6	7.6	1.26

^a^ GI_50_ values are reported as the mean of five experiments and correspond to the agent’s concentration that causes a 50% decrease in net cell growth. Ge, gefitinib.

**Table 2:** Kinase profile of 2-arylmethyl-1-oxo-1,2,3,4-tetrahydropyrazino[1,2-*a*]indole-3-carboxamide analogs over a panel of 18 kinases (DNA-PK, mTOR, 5 PDKs, TBK1, and 10 PI3Ks).

Kinase	% Enzyme Activity ^a^				
	2b	2f	2i	Ref 1	Ref 2
DNA-PK	75.4	96.6	84.5	67.1	67
mTOR/FRAP1	99.2	109.8	101	117.1	118.8
PDK1/PDHK1	96.9	93.2	99.4	89.8	92.6
PDK1/PDPK1	88.7	95	99.2	101.1	95.8
PDK2/PDHK2	88.3	90.1	88.6	89.5	86.1
PDK3/PDHK3	107.7	103.8	101.9	101.8	105.9
PDK4/PDHK4	102.5	101.2	101.2	92.1	106.2
TBK1	123.8	123.3	113.6	88.6	91.5
PI3KC2A	87.7	87.5	89.6	91.3	82
PI3KC3	92.4	95.6	92	94.9	85.8
PI3Kα (p110α/p85α)	238	201.3	376.1	193.4	259.4
PI3Kβ (p110β/p85α)	71.8	70.9	87	69	55.3
PI3Kγ (p110γ)	80.8	82.7	84.7	76.1	85.4
PI3Kδ (p110δ/p85α)	120.1	107.6	110.1	130.4	124.6
PI3K (p110α/p65α)	95.5	89.9	80.1	106.5	103.1
PI3K (p110α(E542K)/p85α)	106.8	100.3	118.1	123.7	121.2
PI3K (p110α(E545K)/p85α)	128.4	120	151.6	130.1	150.9
PI3K (p110α(H1047R)/p85α)	90.6	95.1	95.4	101.6	102.3

^a^ Each enzyme activity was performed in duplicate at 10 μM by Reaction Biology Corporation. The results were normalized using a DMSO control.

## Data Availability

The data presented in this study are available in this article or associated supplementary material.

## Supplementary Materials

The following are available online at https://www.mdpi.com/article/10.3390/ph14100974/s1, Figures S1–S39: ^1^H and ^13^C NMR specta of componds **7a**–**m**, **2a**–**m**, and **1a**–**m**.
