# Supplementary material for: Synthesis and Biological Evaluation of (S)-2-(Substituted arylmethyl)-1-oxo-1,2,3,4-tetrahydropyrazino[1,2-a]indole-3-carboxamide Analogs and Their Synergistic Effect against PTEN-Deficient MDA-MB-468 Cells
Source: Pharmaceuticals (Basel). 2021 Sep 25;14(10):974. doi: 10.3390/ph14100974 (PMC8537755; doi:10.3390/ph14100974)

Article

Synthesis and biological evaluation of (*S*)-2-(substituted arylmethyl)-1-oxo-1,2,3,4-tetrahydropyrazino[1,2-*a*]indole-3-carboxamide analogs and their synergistic effect against PTEN-deficient MDA-MB-468 cells

Ye-Mi Kwon [1,†], Sou Hyun Kim [2,†], Young-Suk Jung [2,*] and Jae-Hwan Kwak [1,*]

1 College of Pharmacy, Kyungsung University, Busan, 48434, Republic of Korea; kym00109@naver.com (Y.-M.K.)
2 College of Pharmacy, Pusan National University, Busan, 46241, Republic of Korea; souhyun@pusan.ac.kr (S.H.K.)
* Correspondence: youngjung@pusan.ac.kr (Y.-S.J.); jhkwak@ks.ac.kr (J.-H.K.);
Tel.: +82-51-510-2816 (Y.-S.J.); +82-51-663-4889 (J.-H.K.);
† These authors contributed equally to this work.

Supplementary Materials

Table of Contents **Page**

1. ^{1}H and ^{13}C NMR spectra **S1-S39**

1. ^{1}H and ^{13}C NMR spectra

^{1}H NMR: Methyl (*S*)-2-(3-nitrobenzyl)-1-oxo-1,2,3,4-tetrahydropyrazino[1,2-*a*]indole-3-carboxylate (7a)

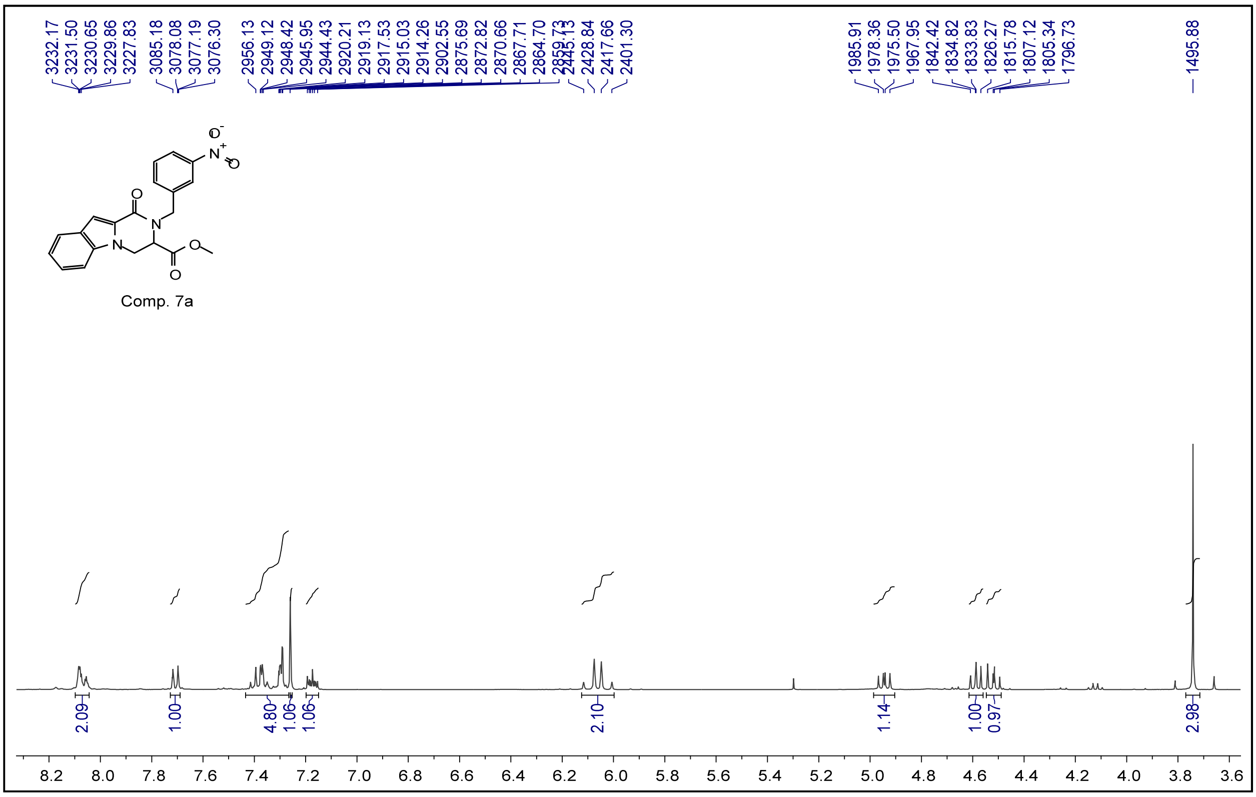

^{13}C NMR: Methyl (*S*)-2-(3-nitrobenzyl)-1-oxo-1,2,3,4-tetrahydropyrazino[1,2-*a*]indole-3-carboxylate (7a)

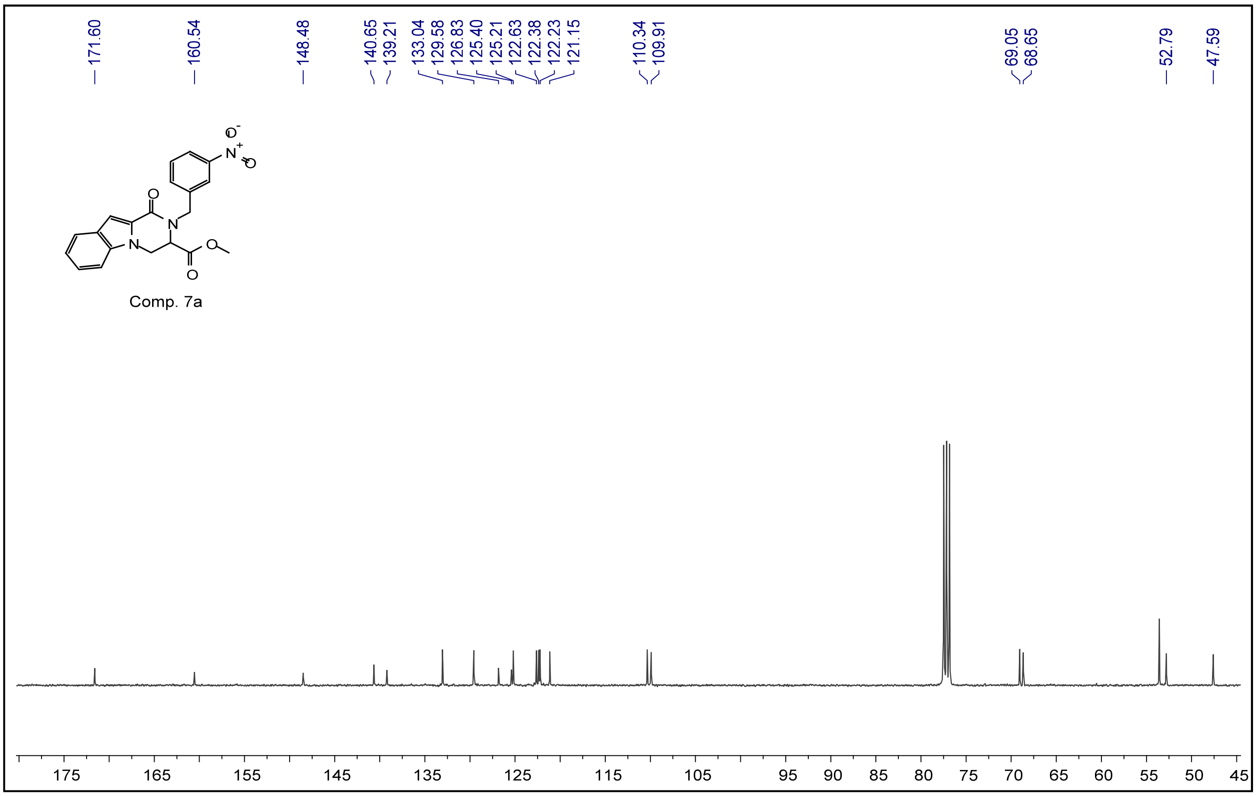

^{1}H NMR: Methyl (*S*)-2-(4-nitrobenzyl)-1-oxo-1,2,3,4-tetrahydropyrazino[1,2-*a*]indole-3-carboxylate (7b)

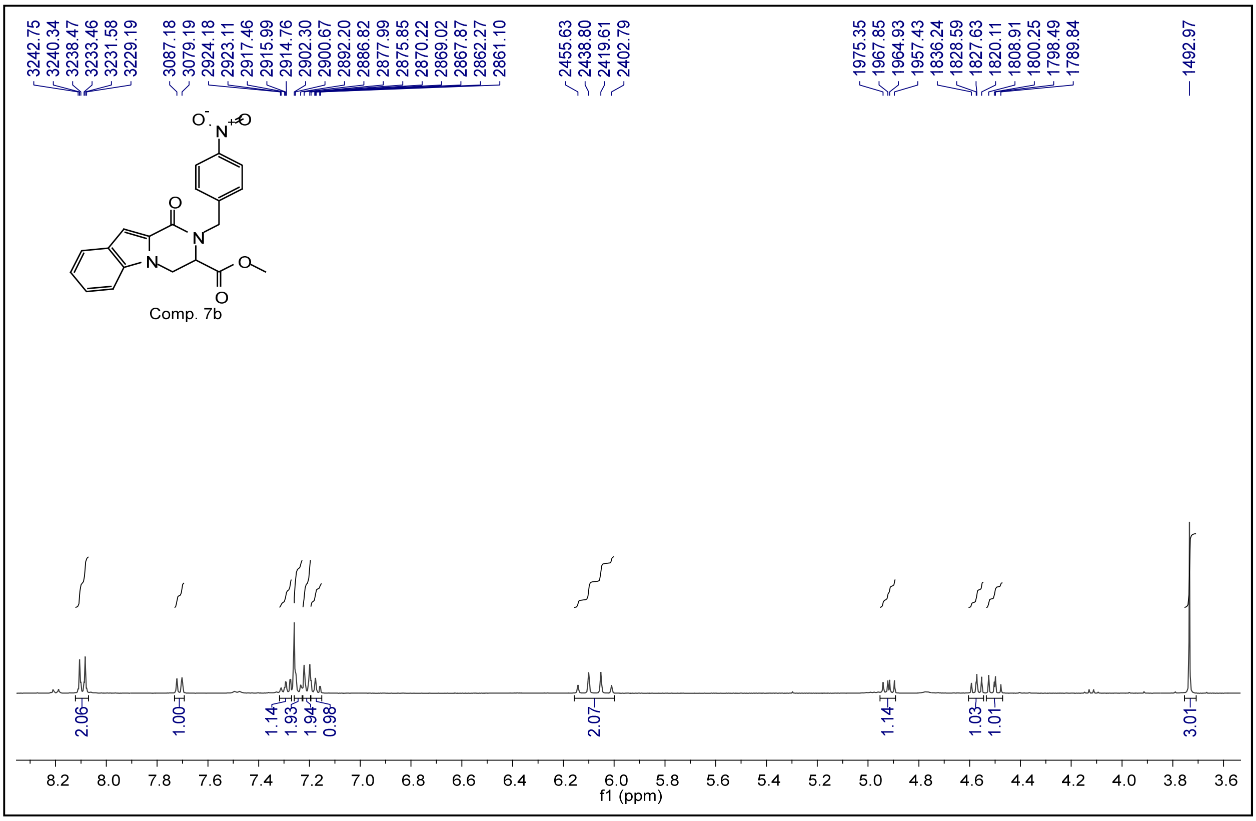

^{13}C NMR: Methyl (*S*)-2-(4-nitrobenzyl)-1-oxo-1,2,3,4-tetrahydropyrazino[1,2-*a*]indole-3-carboxylate (7b)

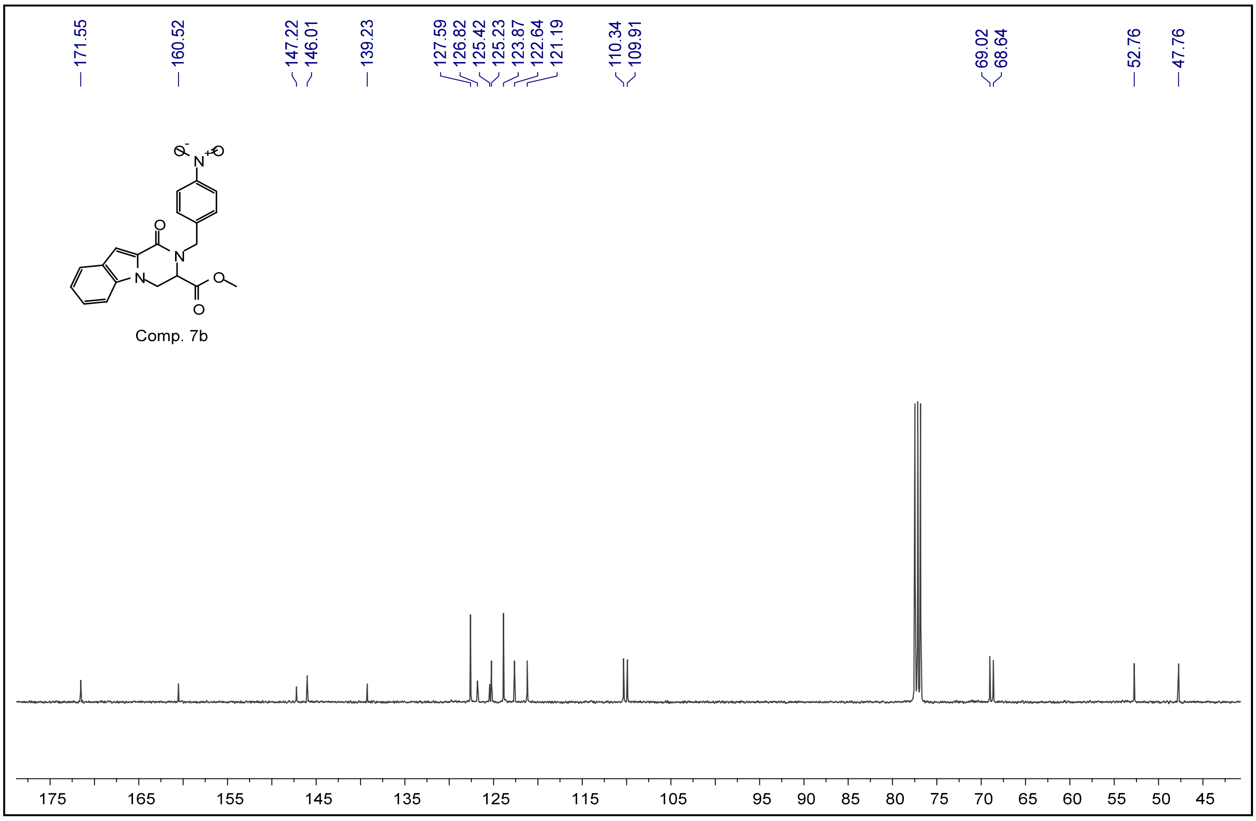

^{1}H NMR: Methyl (*S*)-2-(3-fluorobenzyl)-1-oxo-1,2,3,4-tetrahydropyrazino[1,2-*a*]indole-3-carboxylate (7c)

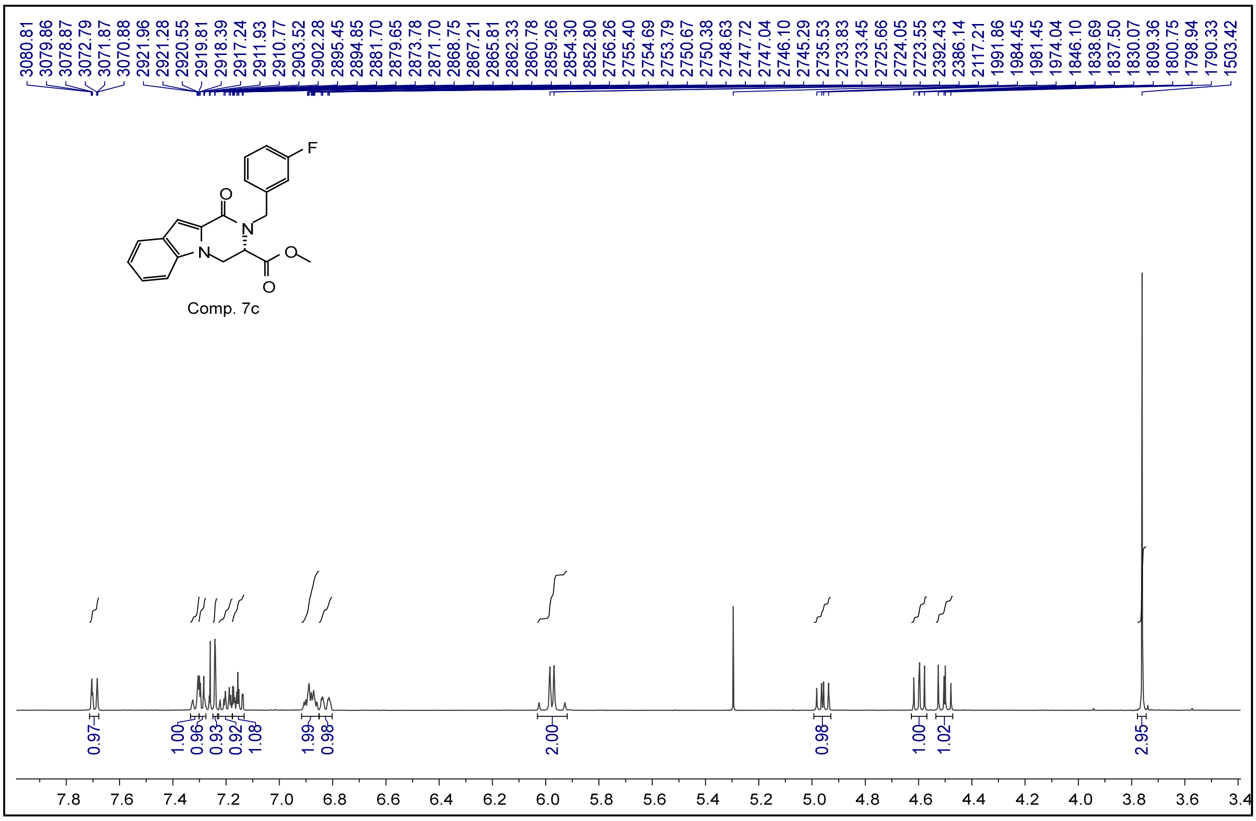

^{13}C NMR: Methyl (*S*)-2-(3-fluorobenzyl)-1-oxo-1,2,3,4-tetrahydropyrazino[1,2-*a*]indole-3-carboxylate (7c)

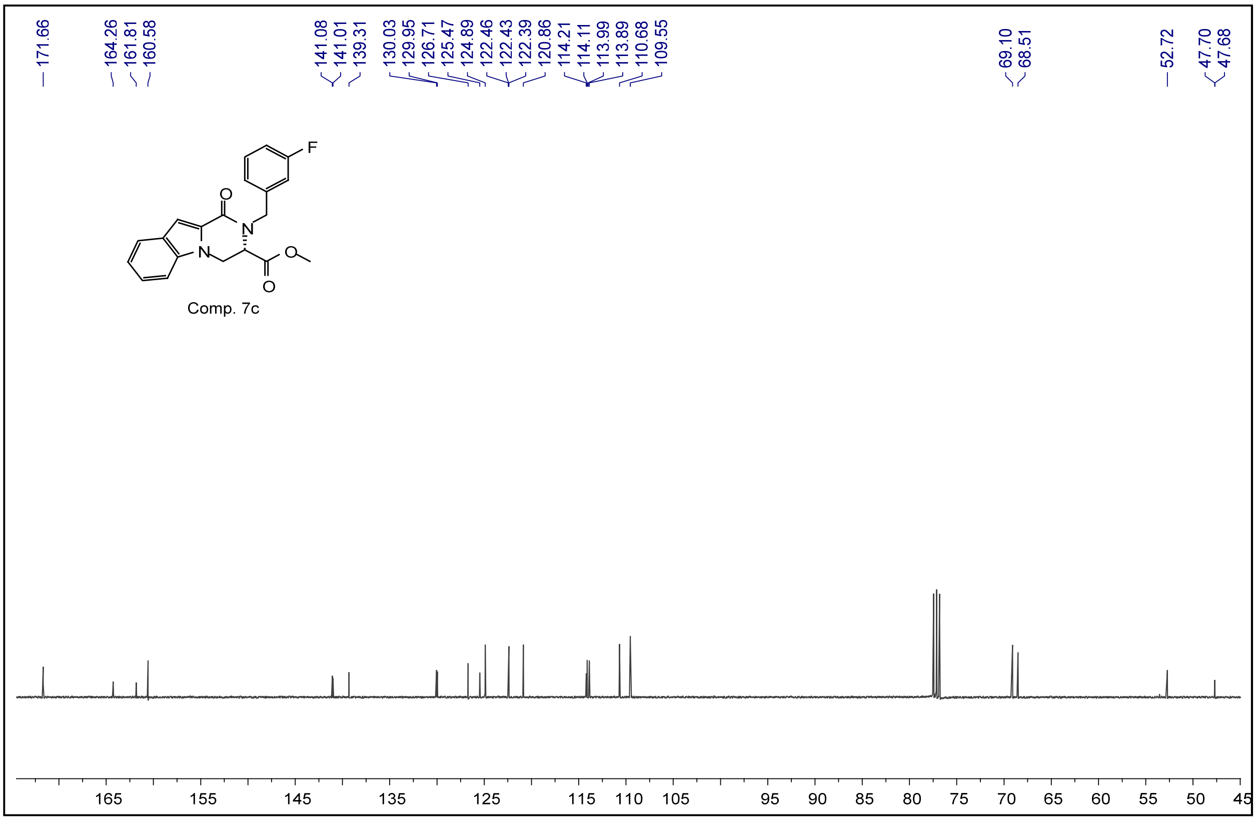

^{1}H NMR: Methyl (*S*)-2-(4-fluorobenzyl)-1-oxo-1,2,3,4-tetrahydropyrazino[1,2-*a*]indole-3-carboxylate (7d)

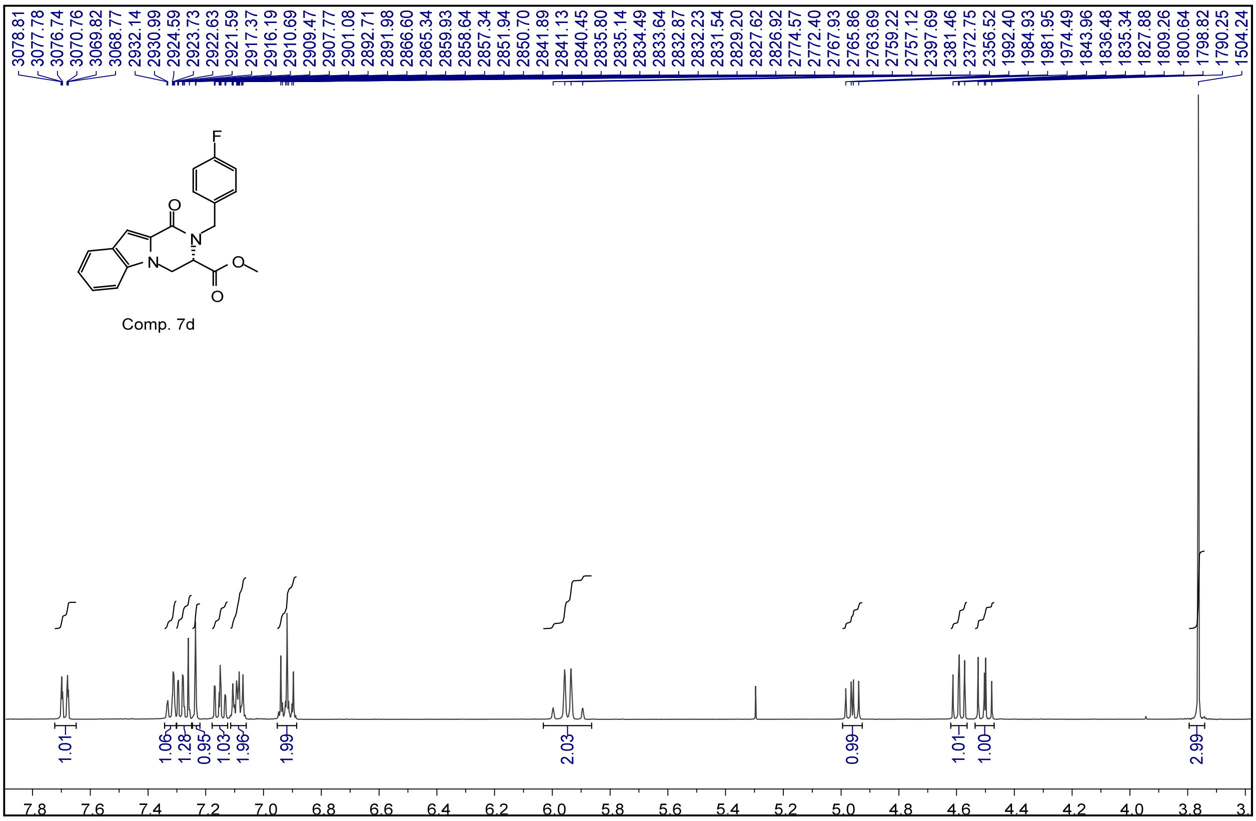

^{13}C NMR: Methyl (*S*)-2-(4-fluorobenzyl)-1-oxo-1,2,3,4-tetrahydropyrazino[1,2-*a*]indole-3-carboxylate (7d)

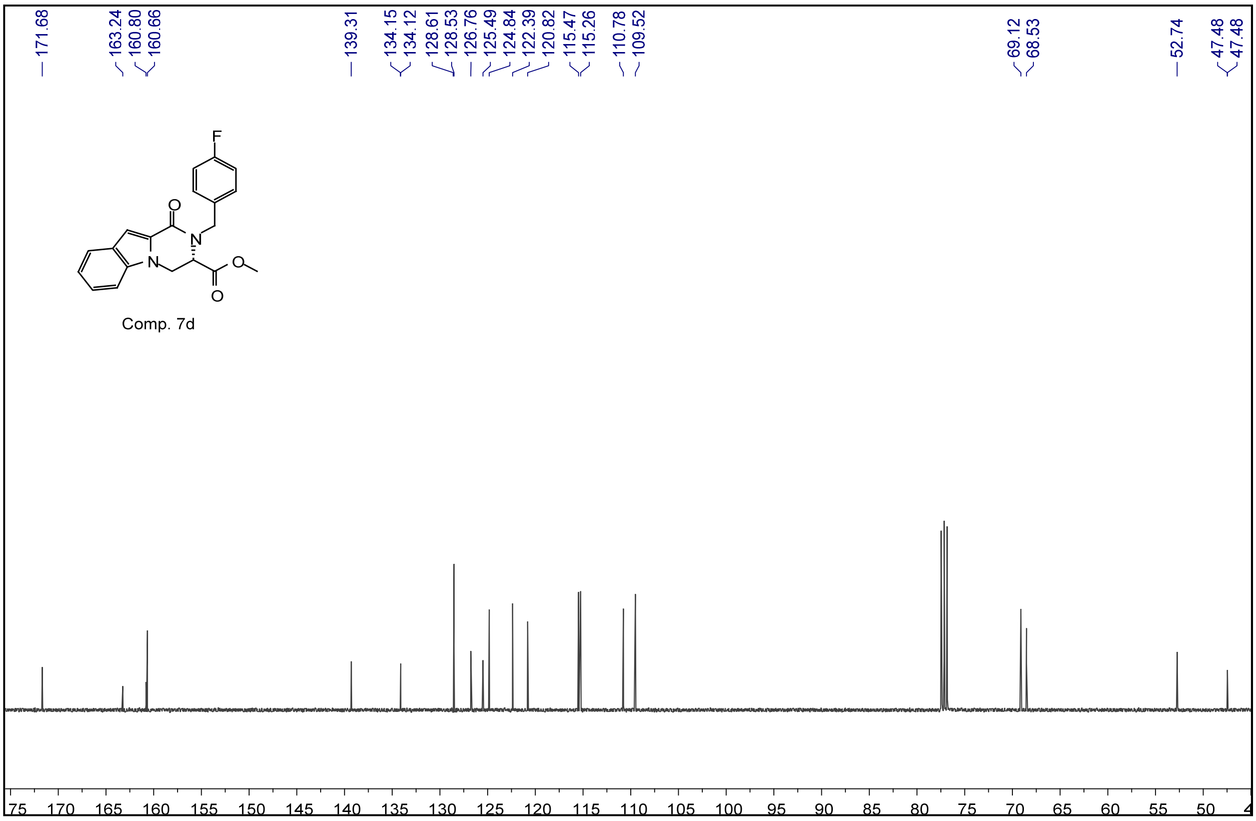

^{1}H NMR: Methyl (*S*)-2-(3-cyanobenzyl)-1-oxo-1,2,3,4-tetrahydropyrazino[1,2-*a*]indole-3-carboxylate (7e)

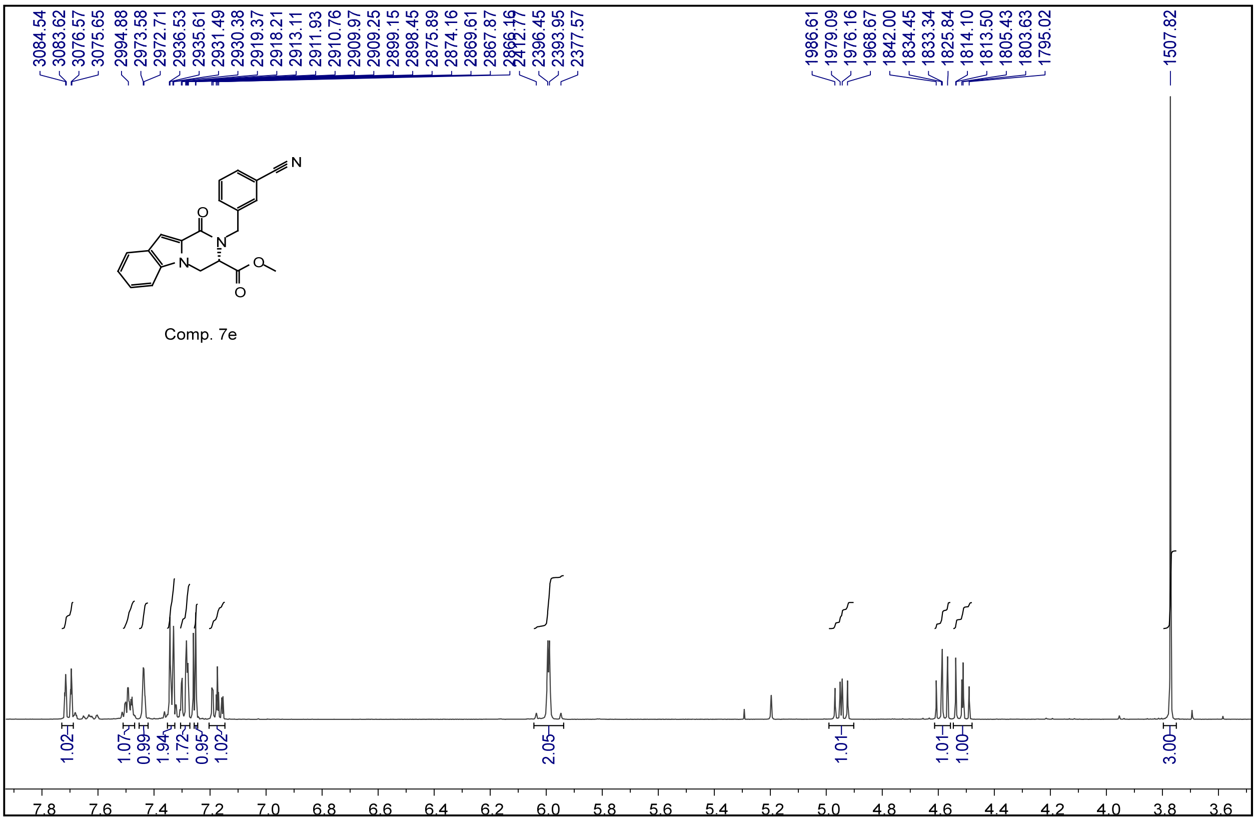

^{13}C NMR: Methyl (*S*)-2-(3-cyanobenzyl)-1-oxo-1,2,3,4-tetrahydropyrazino[1,2-*a*]indole-3-carboxylate (7e)

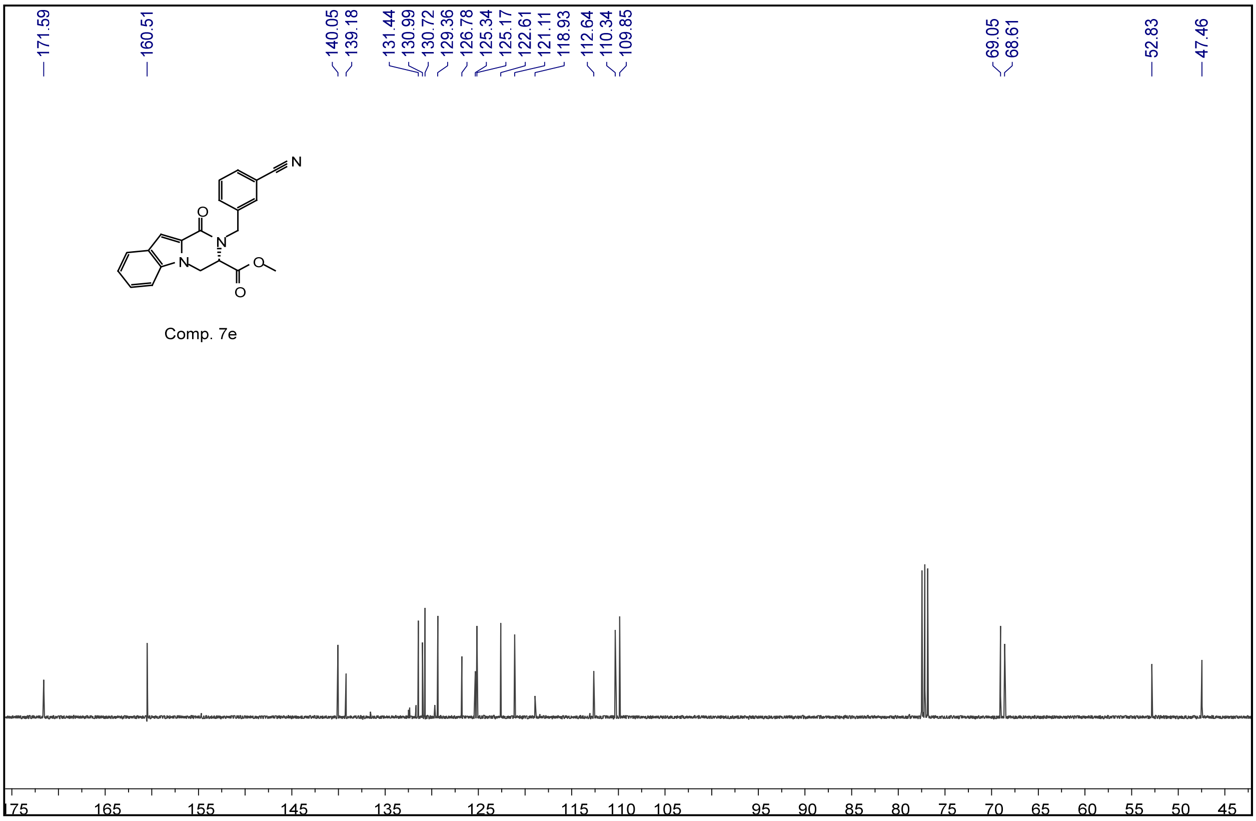

^{1}H NMR: Methyl (*S*)-2-(4-cyanobenzyl)-1-oxo-1,2,3,4-tetrahydropyrazino[1,2-*a*]indole-3-carboxylate (7f)

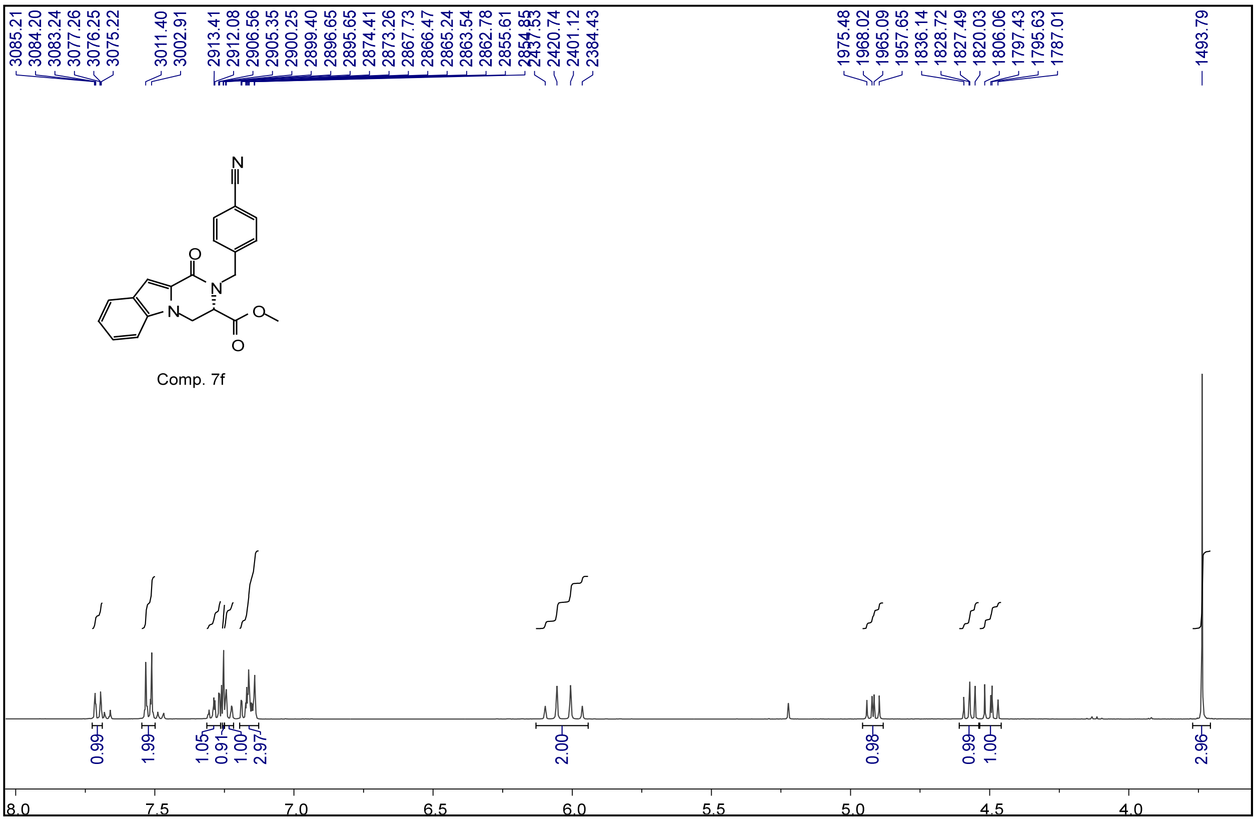

^{13}C NMR: Methyl (*S*)-2-(4-cyanobenzyl)-1-oxo-1,2,3,4-tetrahydropyrazino[1,2-*a*]indole-3-carboxylate (7f)

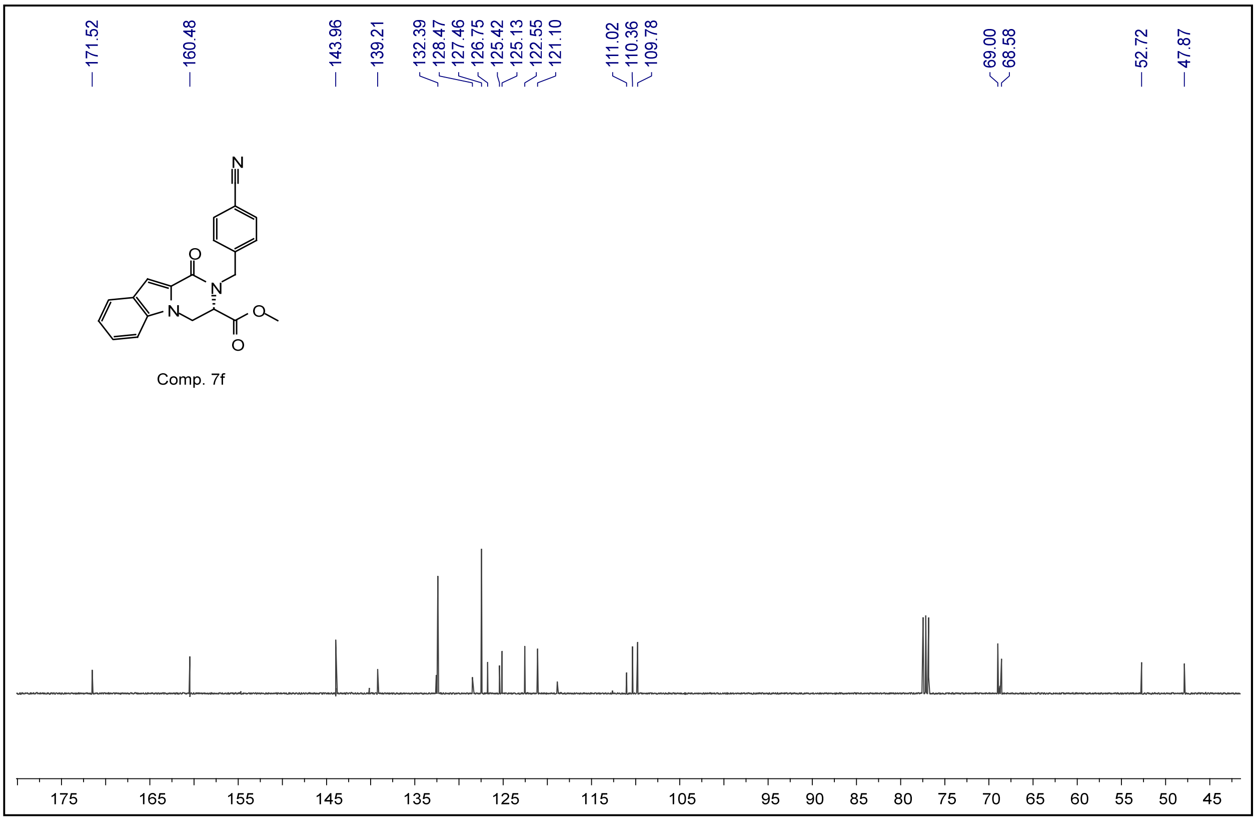

^{1}H NMR: Methyl (*S*)-2-(4-trifluoromethylbenzyl)-1-oxo-1,2,3,4-tetrahydropyrazino[1,2-*a*]indole-3-carboxylate (7g)

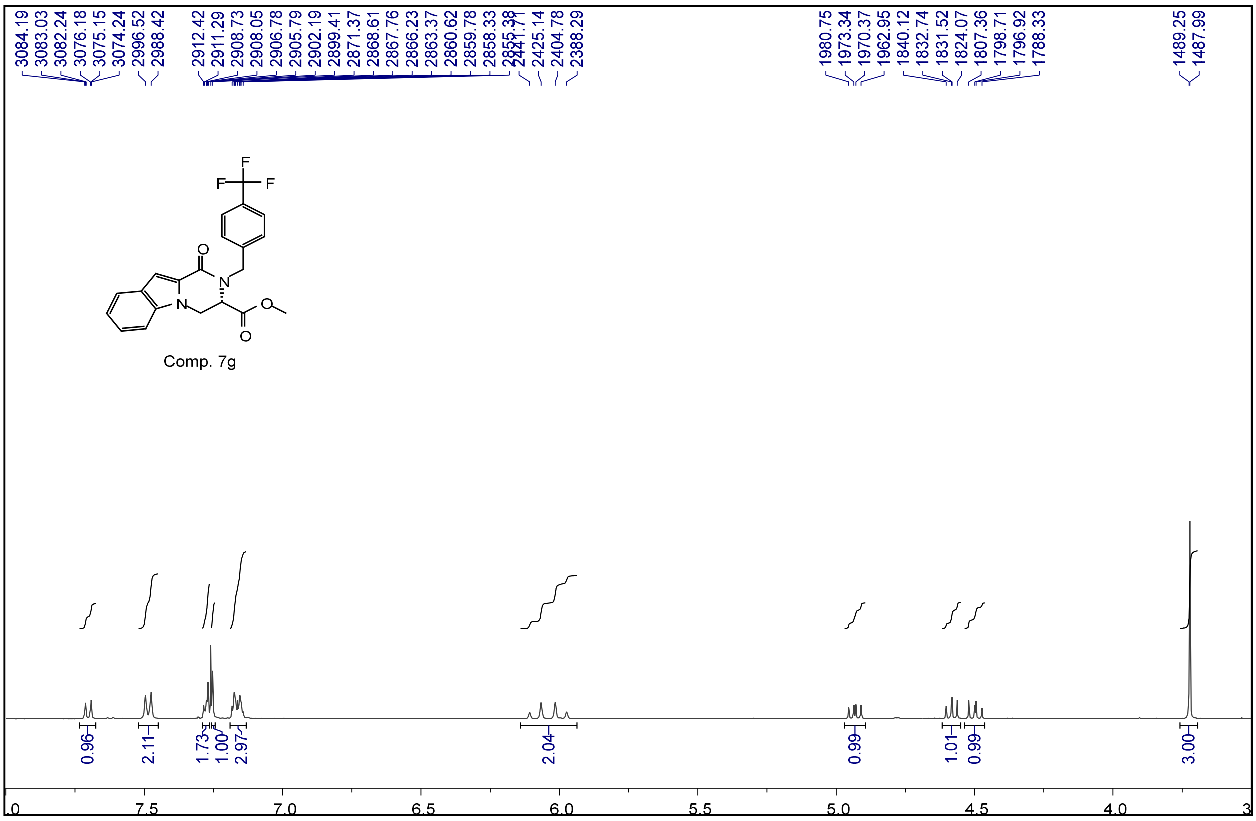

^{13}C NMR: Methyl (*S*)-2-(4-trifluoromethylbenzyl)-1-oxo-1,2,3,4-tetrahydropyrazino[1,2-*a*]indole-3-carboxylate (7g)

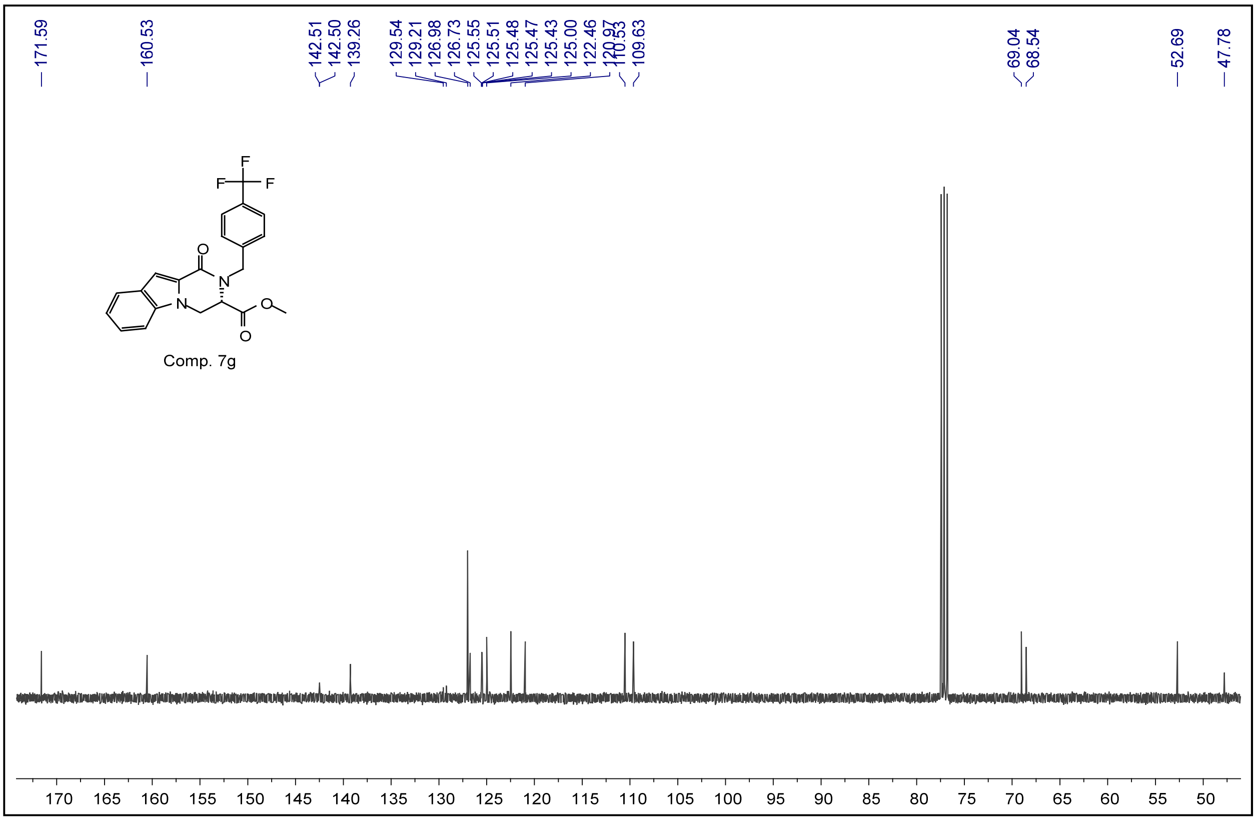

^{1}H NMR: Methyl (*S*)-2-(3-trifluoromethoxylbenzyl)-1-oxo-1,2,3,4-tetrahydropyrazino[1,2-*a*]indole-3-carboxylate (7h)

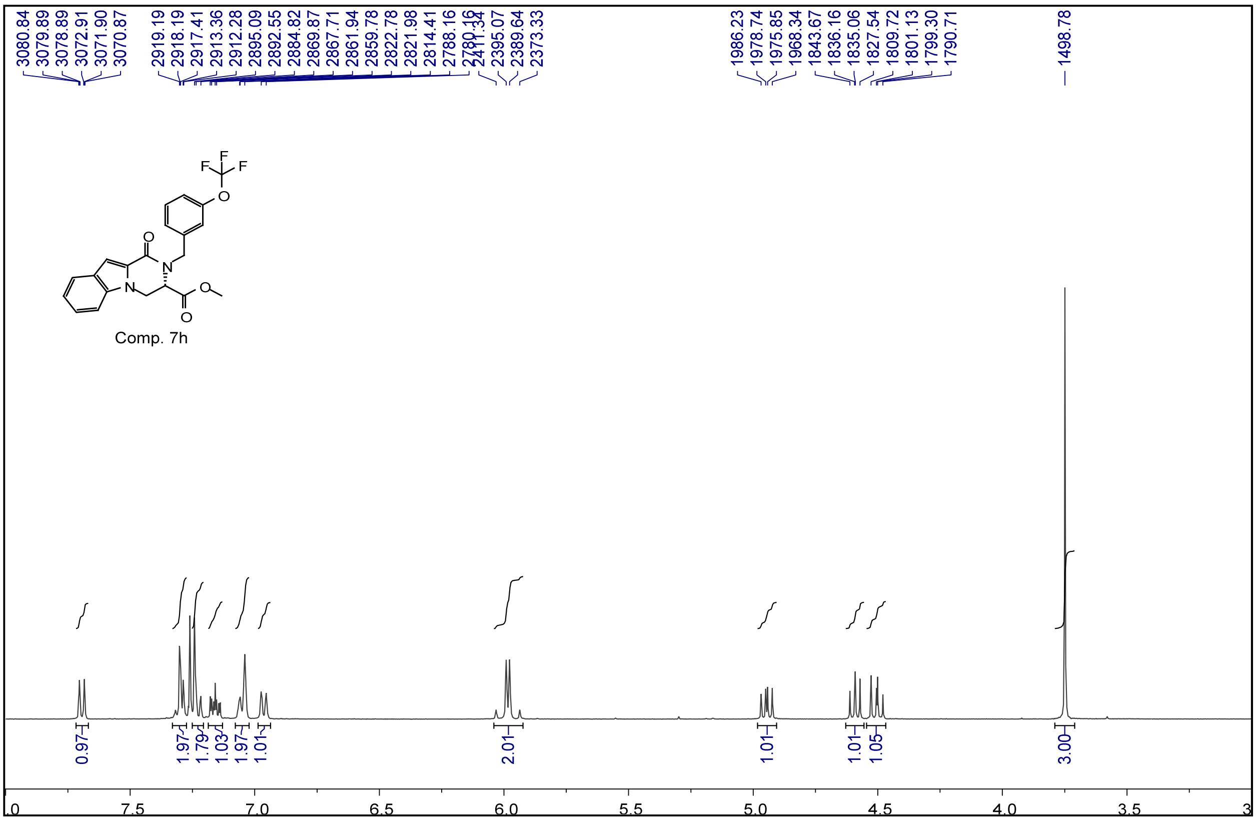

^{13}C NMR: Methyl (*S*)-2-(3-trifluoromethoxylbenzyl)-1-oxo-1,2,3,4-tetrahydropyrazino[1,2-*a*]indole-3-carboxylate (7h)

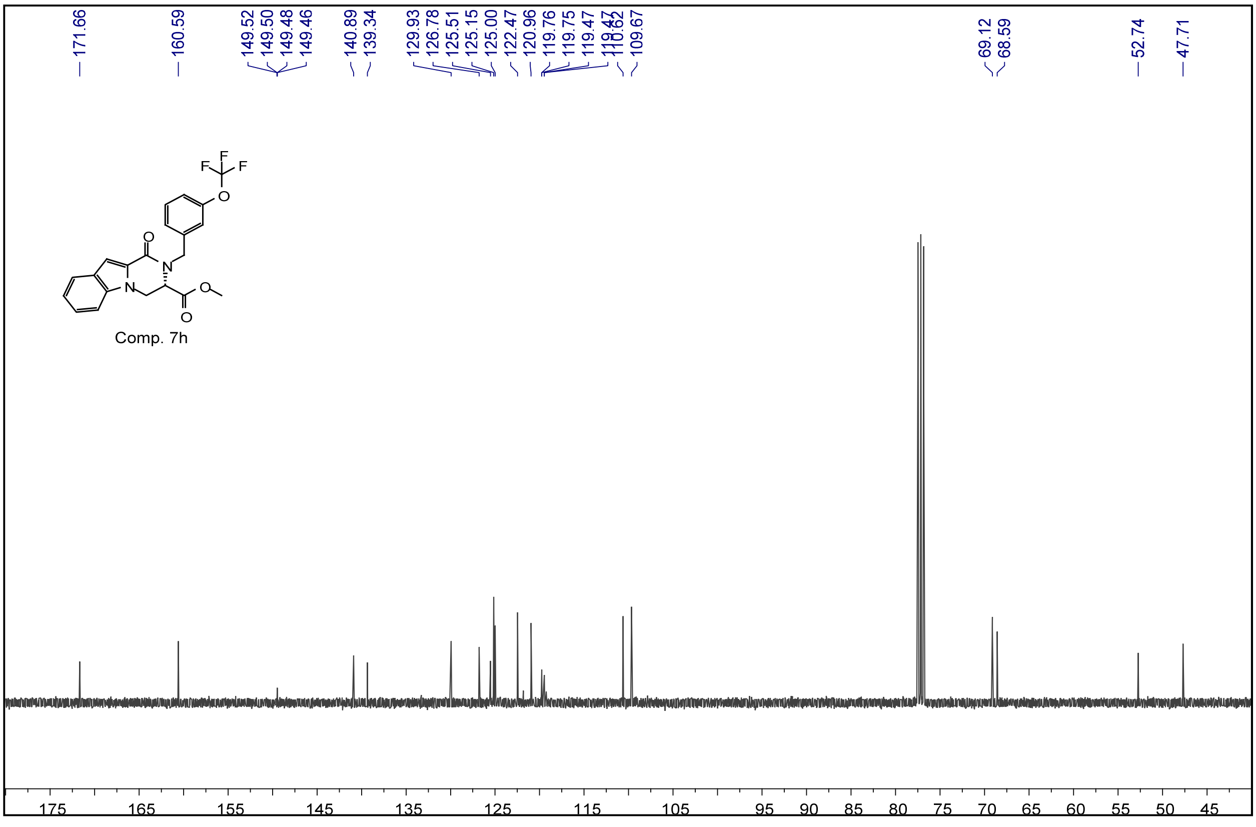

^{1}H NMR: Methyl (*S*)-2-(4-trifluoromethoxylbenzyl)-1-oxo-1,2,3,4-tetrahydropyrazino[1,2-*a*]indole-3-carboxylate (7i)

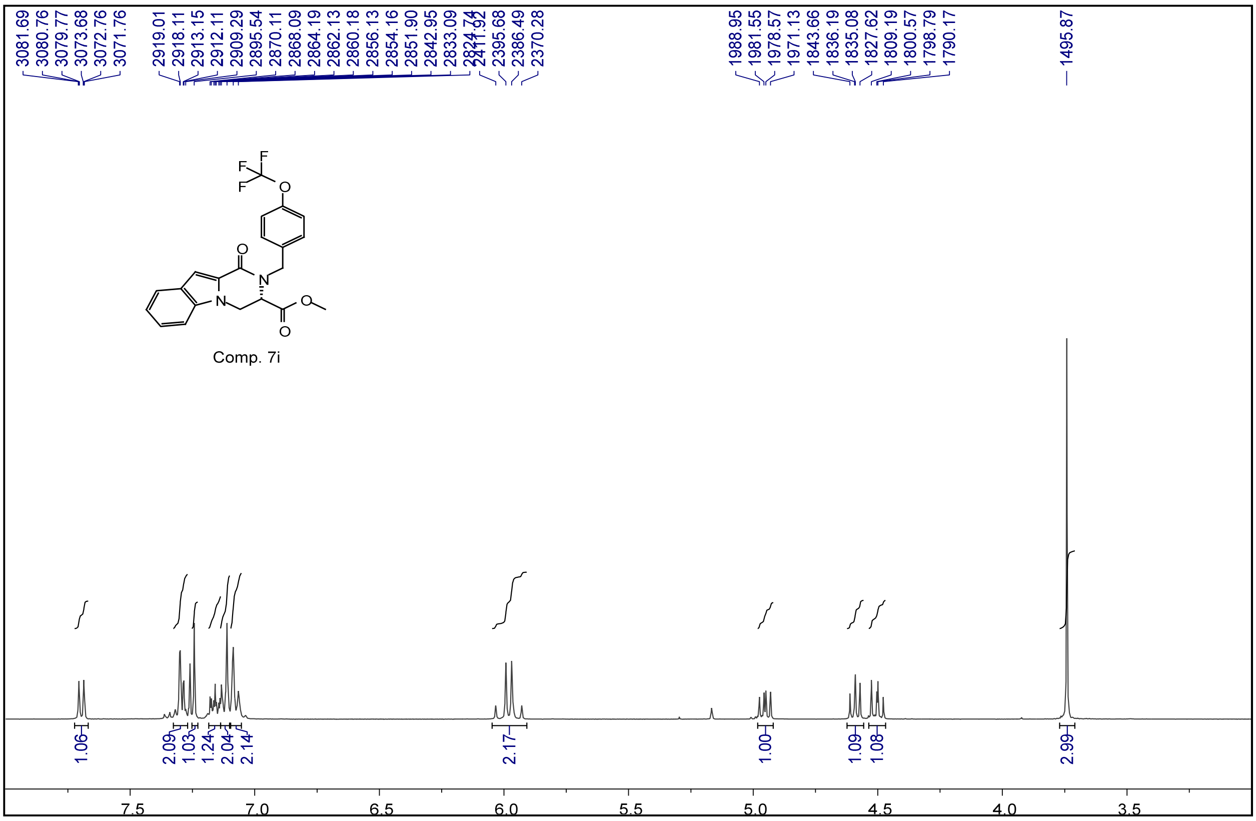

^{13}C NMR: Methyl (*S*)-2-(4-trifluoromethoxylbenzyl)-1-oxo-1,2,3,4-tetrahydropyrazino[1,2-*a*]indole-3-carboxylate (7i)

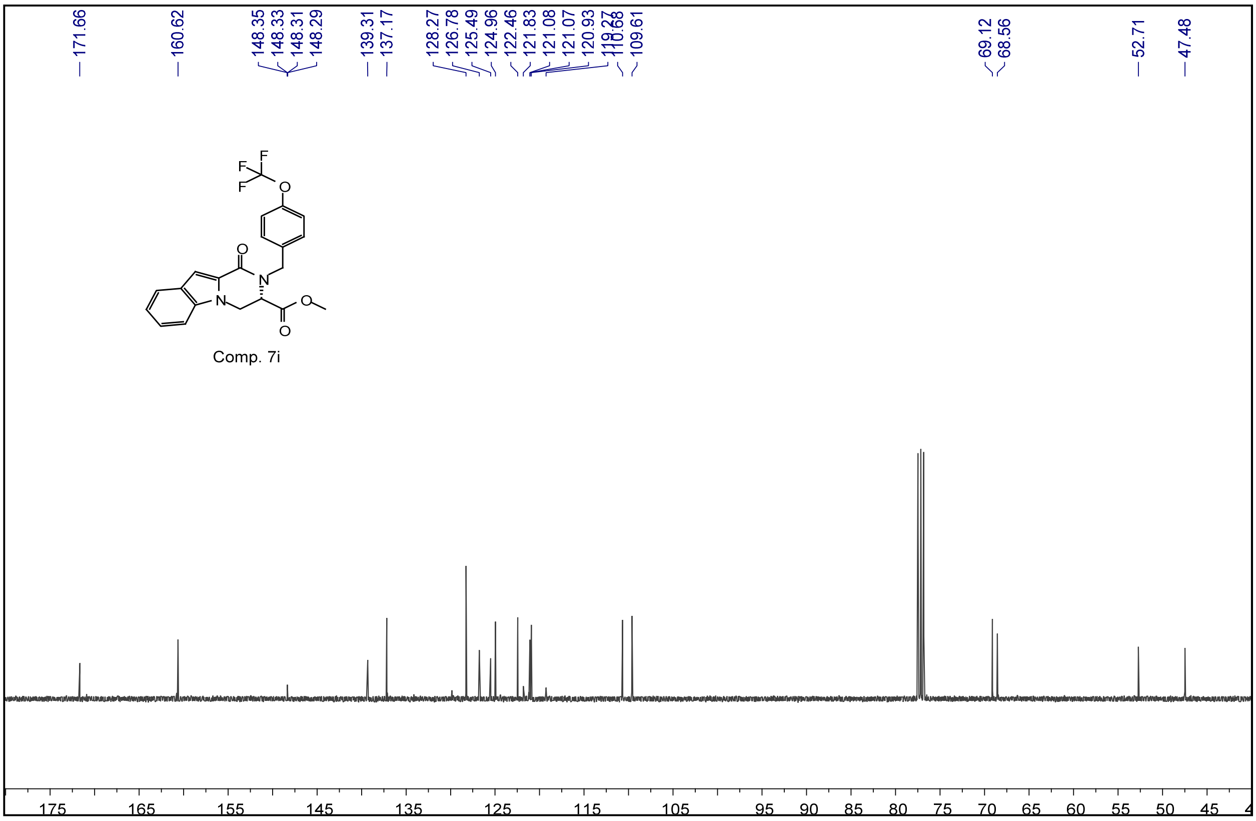

^{1}H NMR: Methyl (*S*)-2-(3-methylbenzyl)-1-oxo-1,2,3,4-tetrahydropyrazino[1,2-*a*]indole-3-carboxylate (7j)

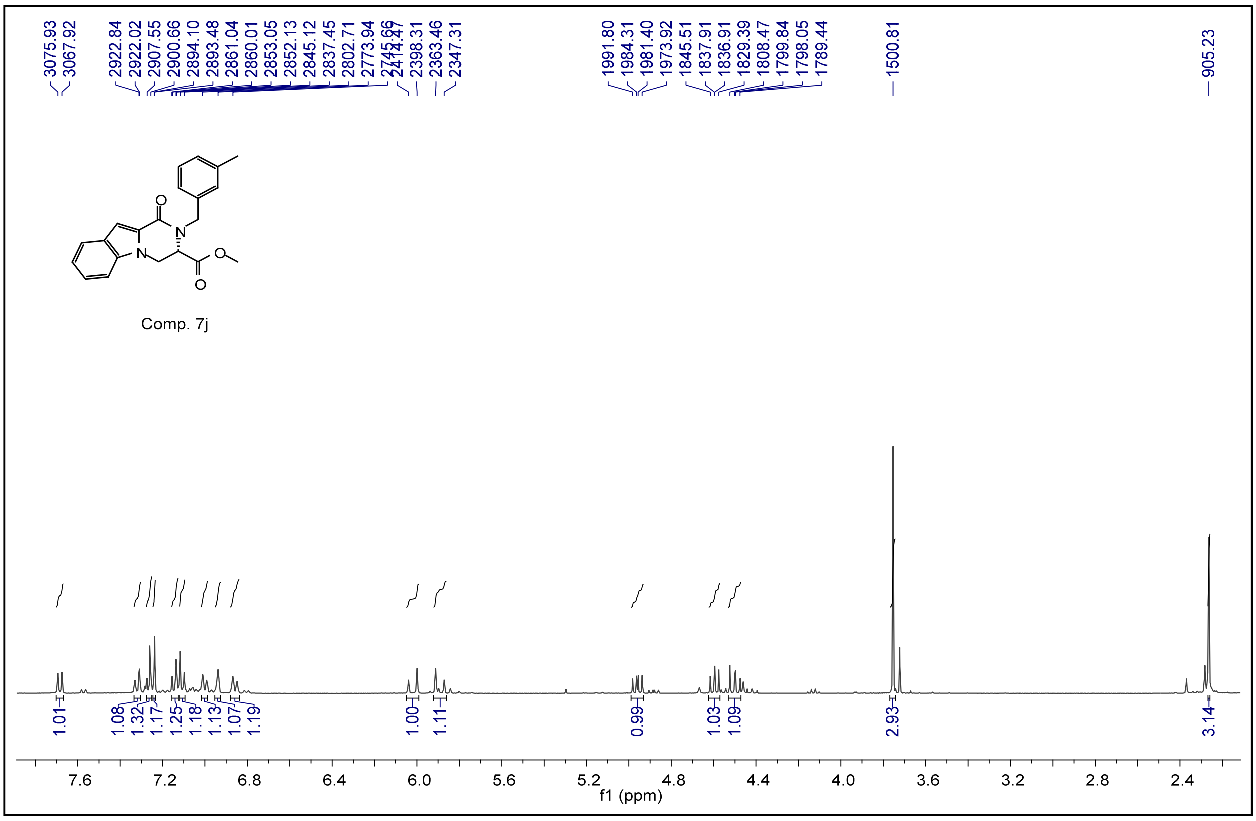

^{13}C NMR: Methyl (*S*)-2-(3-methylbenzyl)-1-oxo-1,2,3,4-tetrahydropyrazino[1,2-*a*]indole-3-carboxylate (7j)

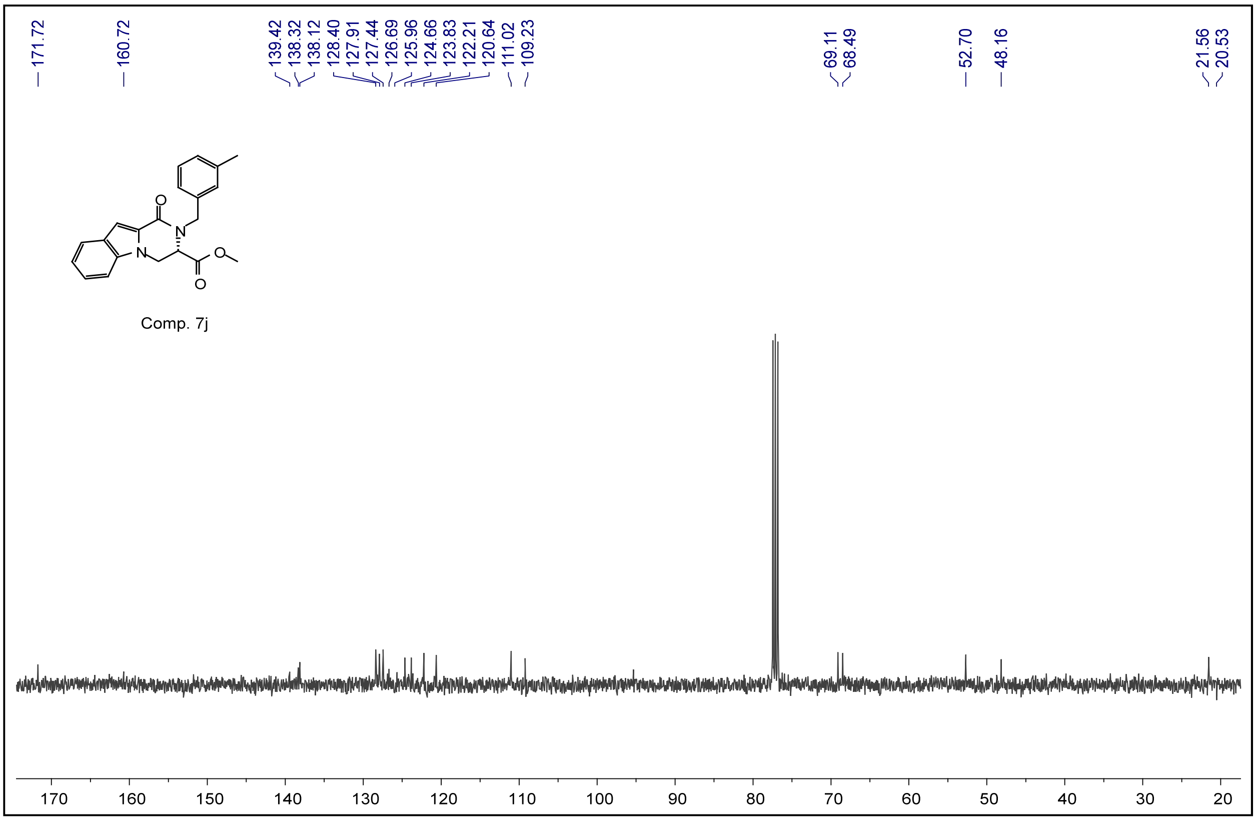

^{1}H NMR: Methyl (*S*)-2-(4-methylbenzyl)-1-oxo-1,2,3,4-tetrahydropyrazino[1,2-*a*]indole-3-carboxylate (7k)

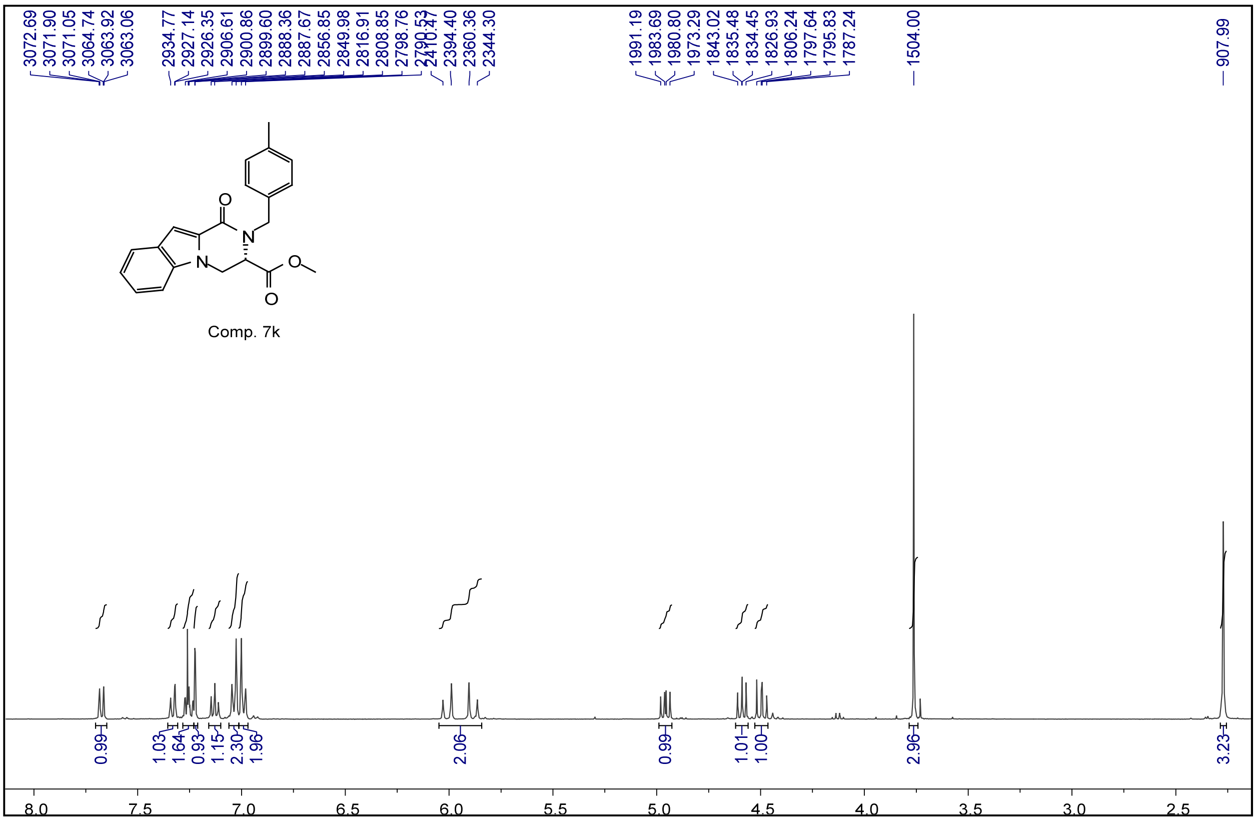

^{13}C NMR: Methyl (*S*)-2-(4-methylbenzyl)-1-oxo-1,2,3,4-tetrahydropyrazino[1,2-*a*]indole-3-carboxylate (7k)

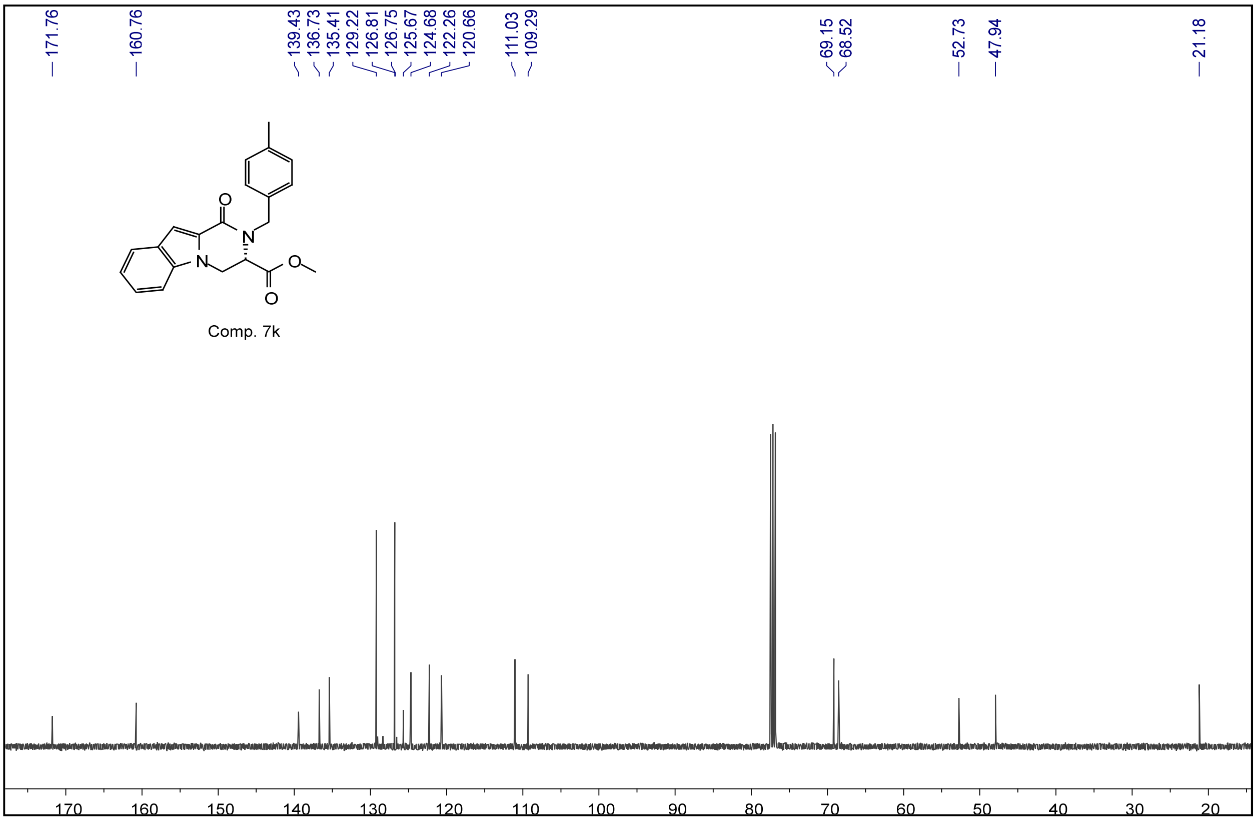

^{1}H NMR: Methyl (*S*)-2-(naphthalen-2-ylmethyl)-1-oxo-1,2,3,4-tetrahydropyrazino[1,2-*a*]indole-3-carboxylate (7l)

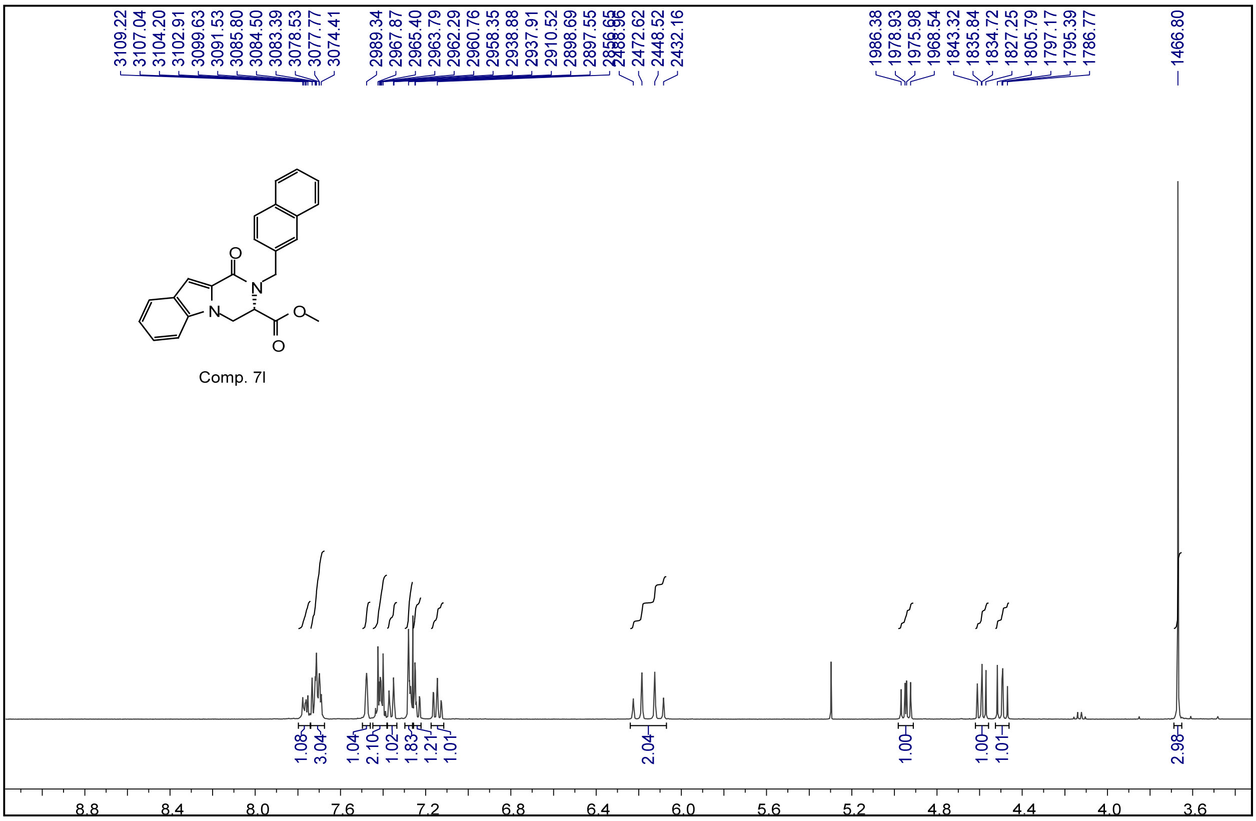

^{13}C NMR: Methyl (*S*)-2-(naphthalen-2-ylmethyl)-1-oxo-1,2,3,4-tetrahydropyrazino[1,2-*a*]indole-3-carboxylate (7l)

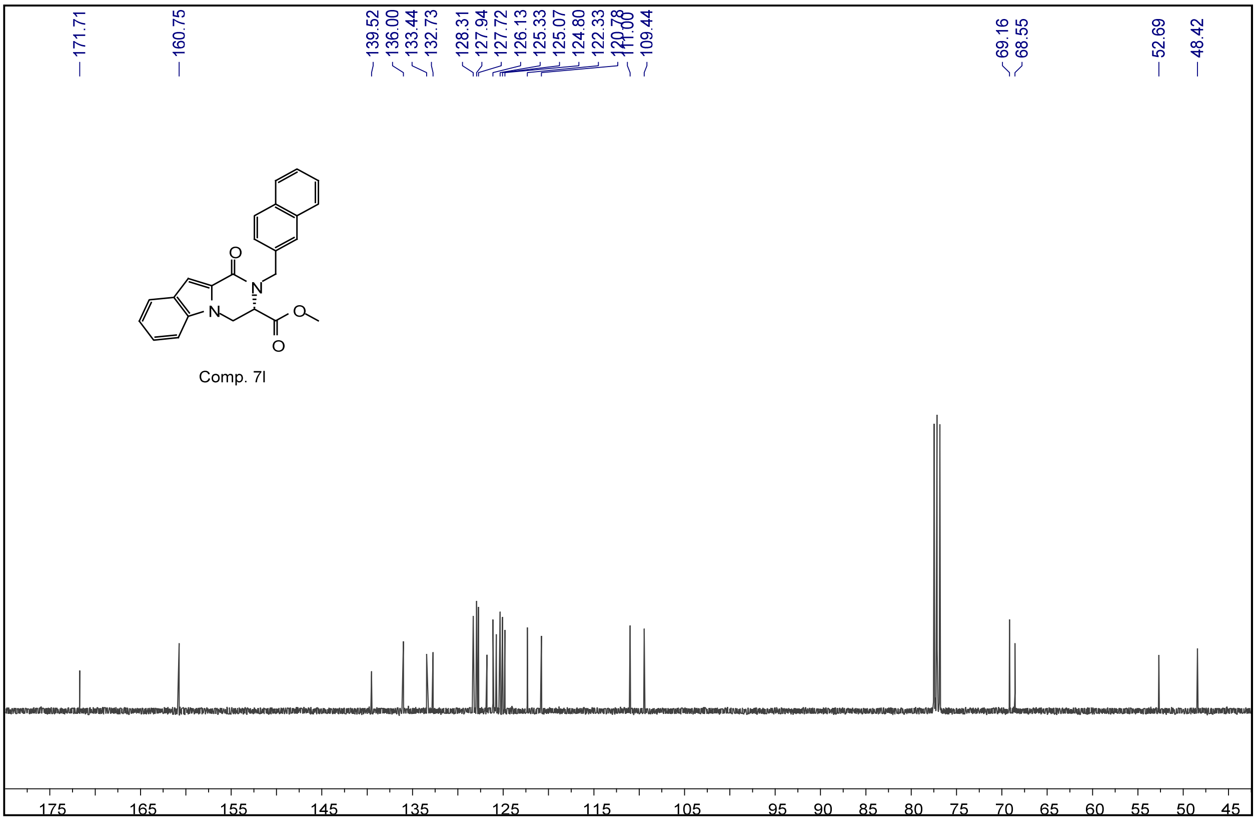

^{1}H NMR: Methyl (*S*)-2-([1,1'-biphenyl]-4-ylmethyl)-1-oxo-1,2,3,4-tetrahydropyrazino[1,2-*a*]indole-3-carboxylate (7m)

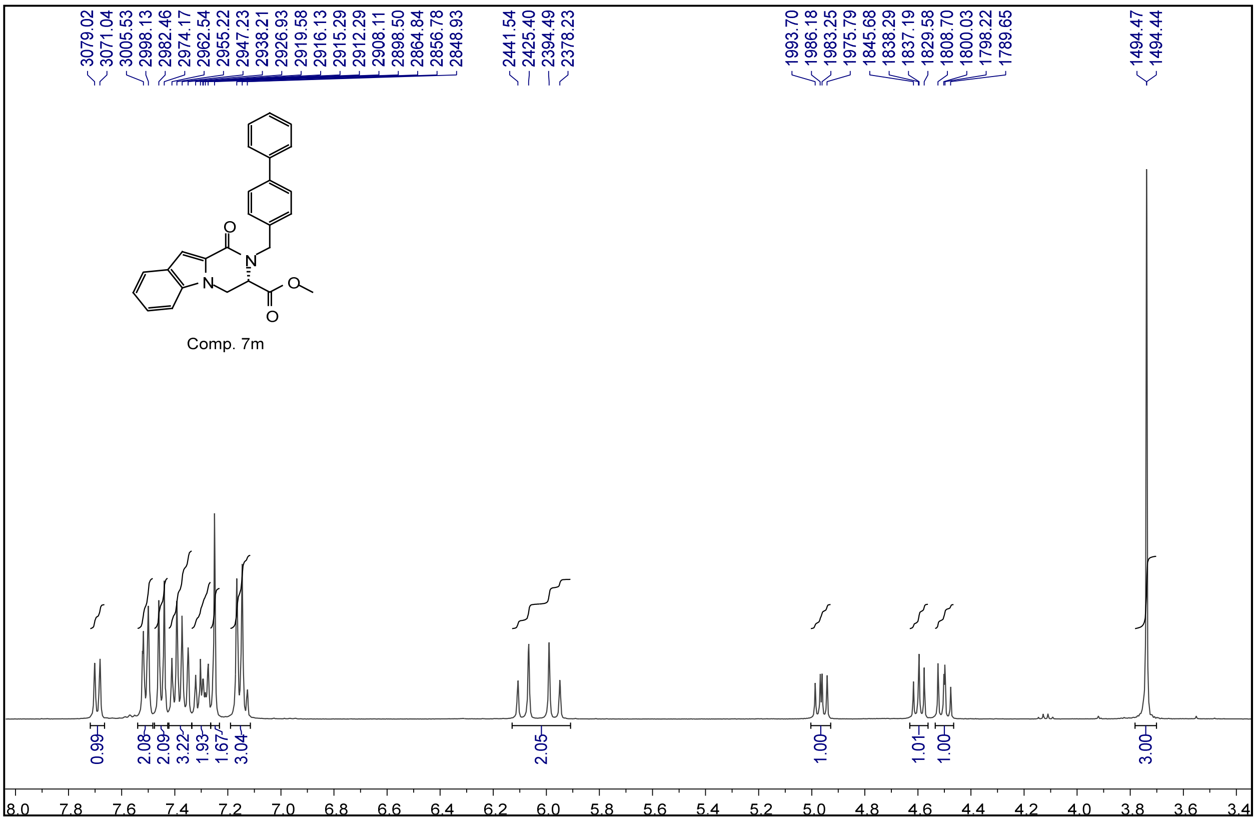

^{13}C NMR: Methyl (*S*)-2-([1,1'-biphenyl]-4-ylmethyl)-1-oxo-1,2,3,4-tetrahydropyrazino[1,2-*a*]indole-3-carboxylate (7m)

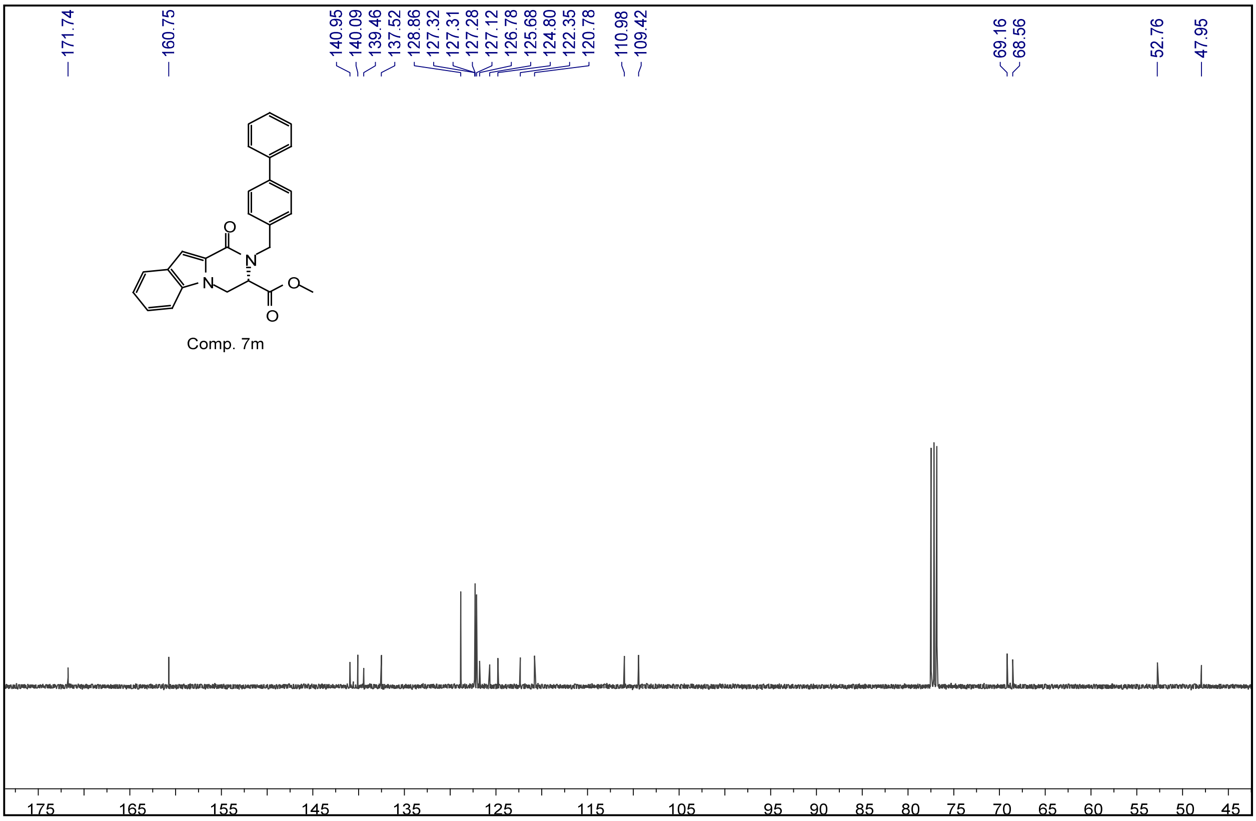

^{1}H NMR: (*S*)-*N*-(Furan-2-ylmethyl)-2-(3-nitrobenzyl)-1-oxo-1,2,3,4-tetrahydropyrazino[1,2-*a*]indole-3-carboxamide (1a)

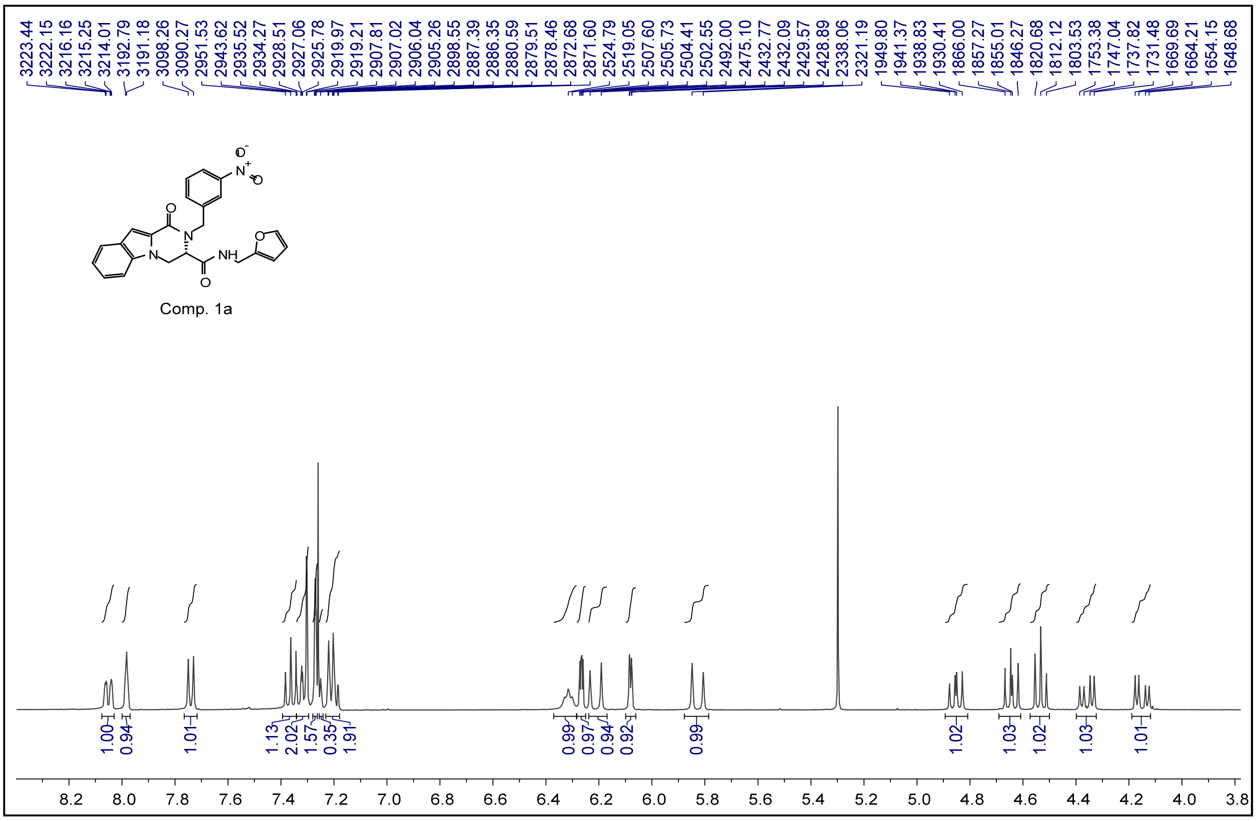

^{13}C NMR: (*S*)-*N*-(Furan-2-ylmethyl)-2-(3-nitrobenzyl)-1-oxo-1,2,3,4-tetrahydropyrazino[1,2-*a*]indole-3-carboxamide (1a)

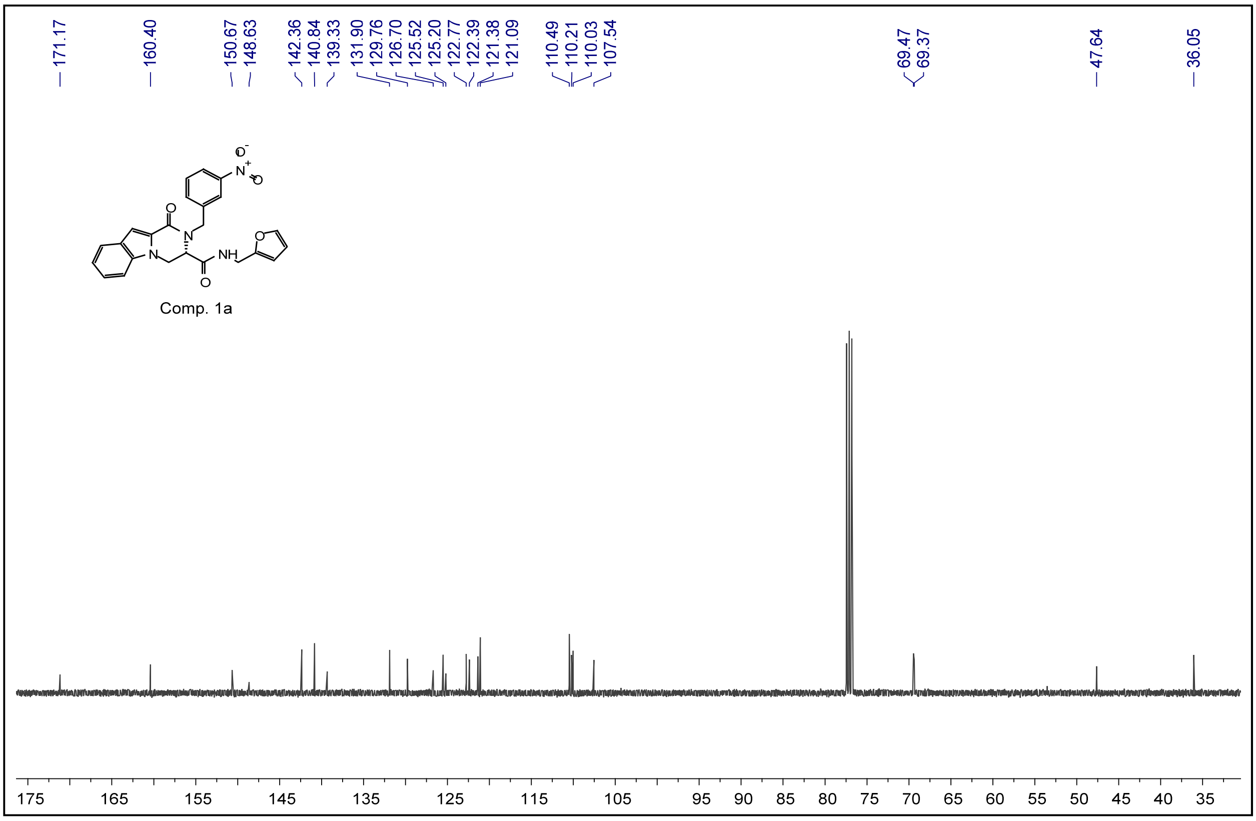

^{1}H NMR: (*S*)-*N*-(Furan-2-ylmethyl)-2-(4-nitrobenzyl)-1-oxo-1,2,3,4-tetrahydropyrazino[1,2-*a*]indole-3-carboxamide (1b)

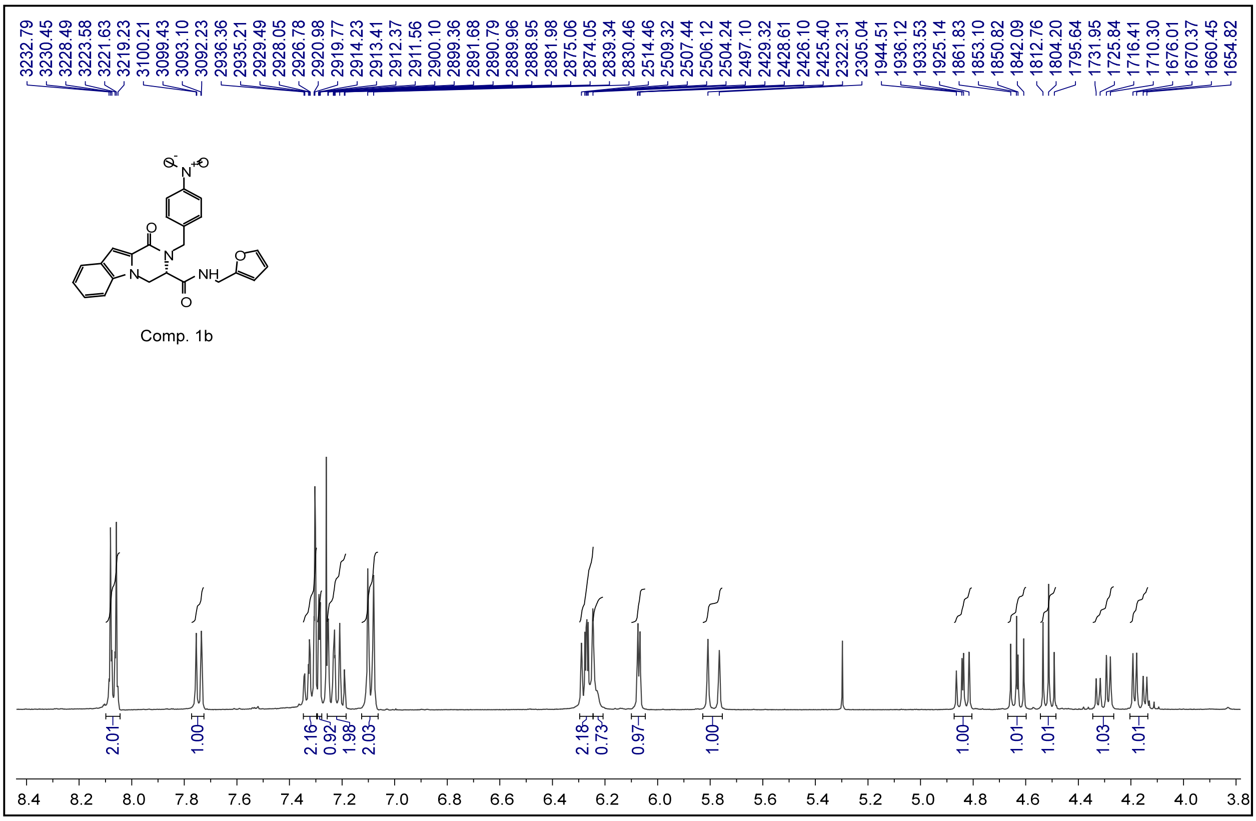

^{13}C NMR: (*S*)-*N*-(Furan-2-ylmethyl)-2-(4-nitrobenzyl)-1-oxo-1,2,3,4-tetrahydropyrazino[1,2-*a*]indole-3-carboxamide (1b)

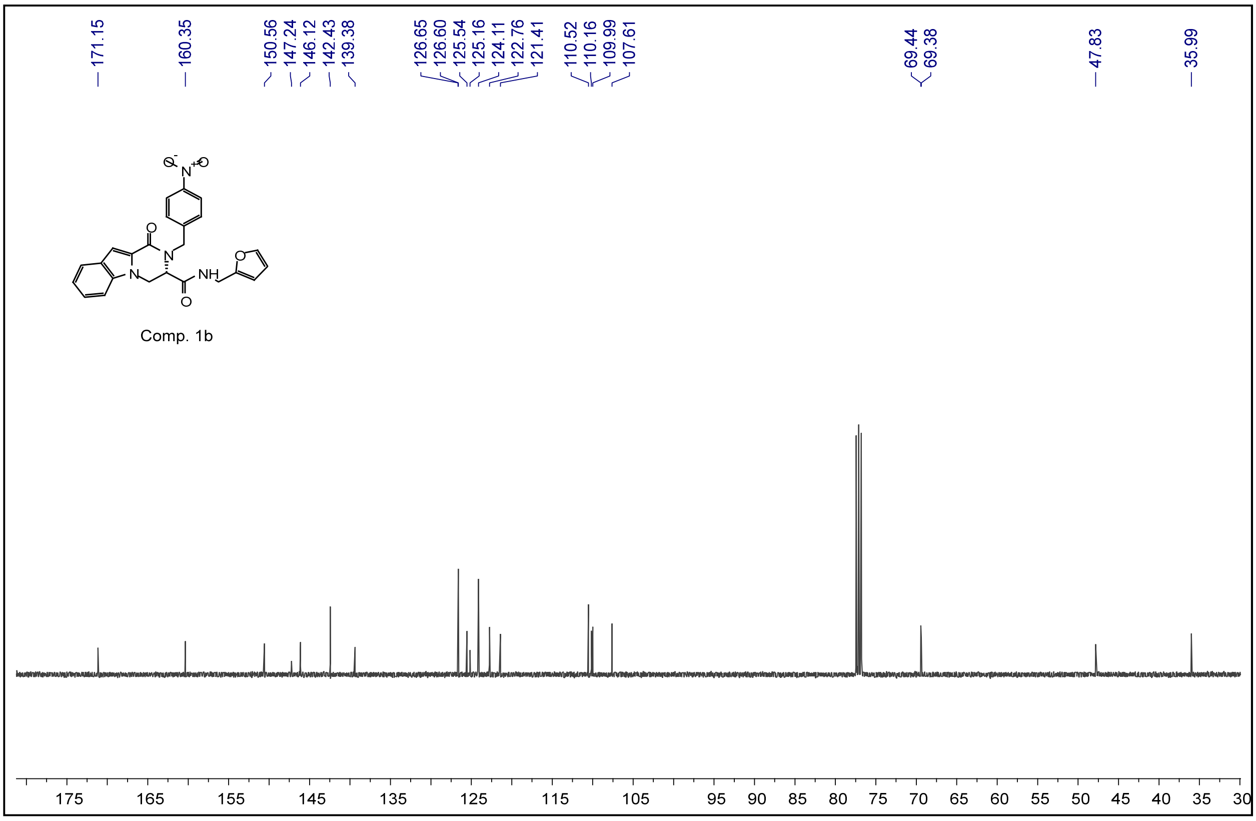

^{1}H NMR: (*S*)-*N*-(Furan-2-ylmethyl)-2-(3-fluorobenzyl)-1-oxo-1,2,3,4-tetrahydropyrazino[1,2-*a*]indole-3-carboxamide (1c)

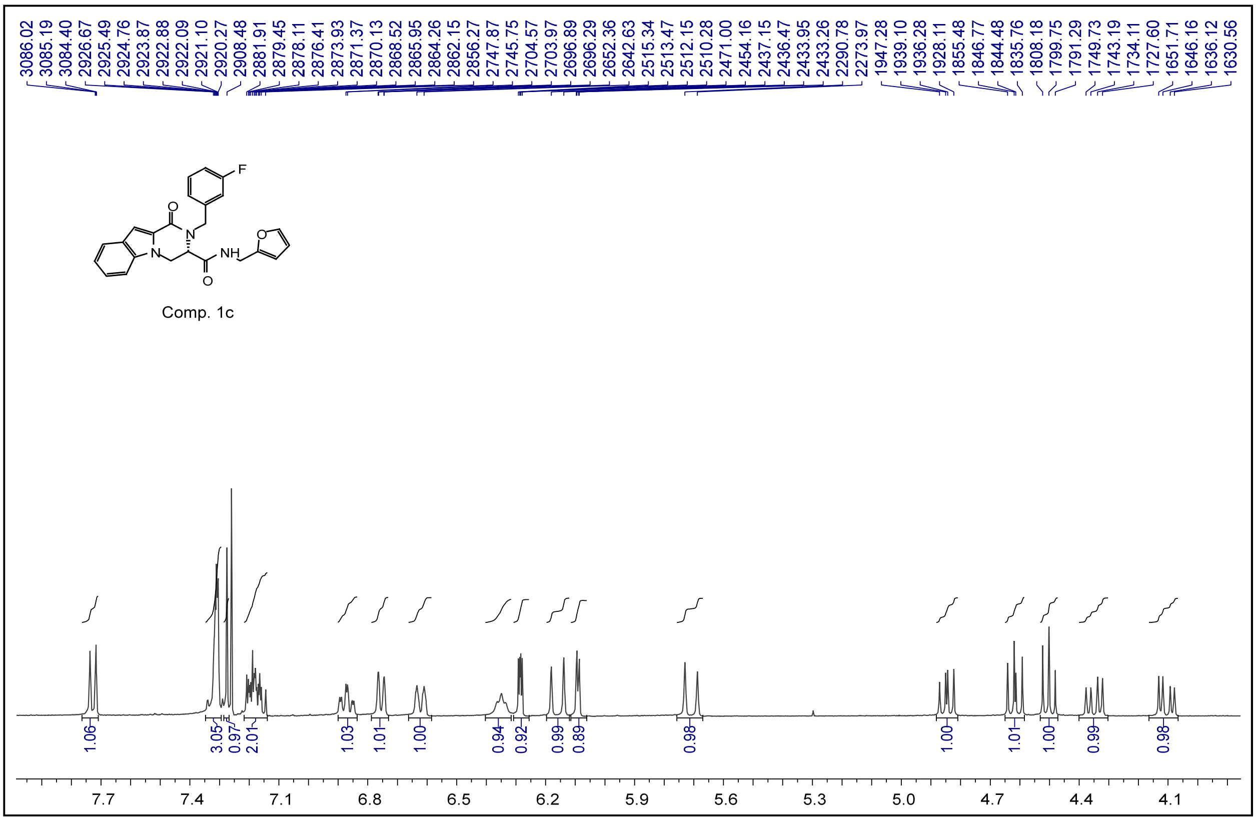

^{13}C NMR: (*S*)-*N*-(Furan-2-ylmethyl)-2-(3-fluorobenzyl)-1-oxo-1,2,3,4-tetrahydropyrazino[1,2-*a*]indole-3-carboxamide (1c)

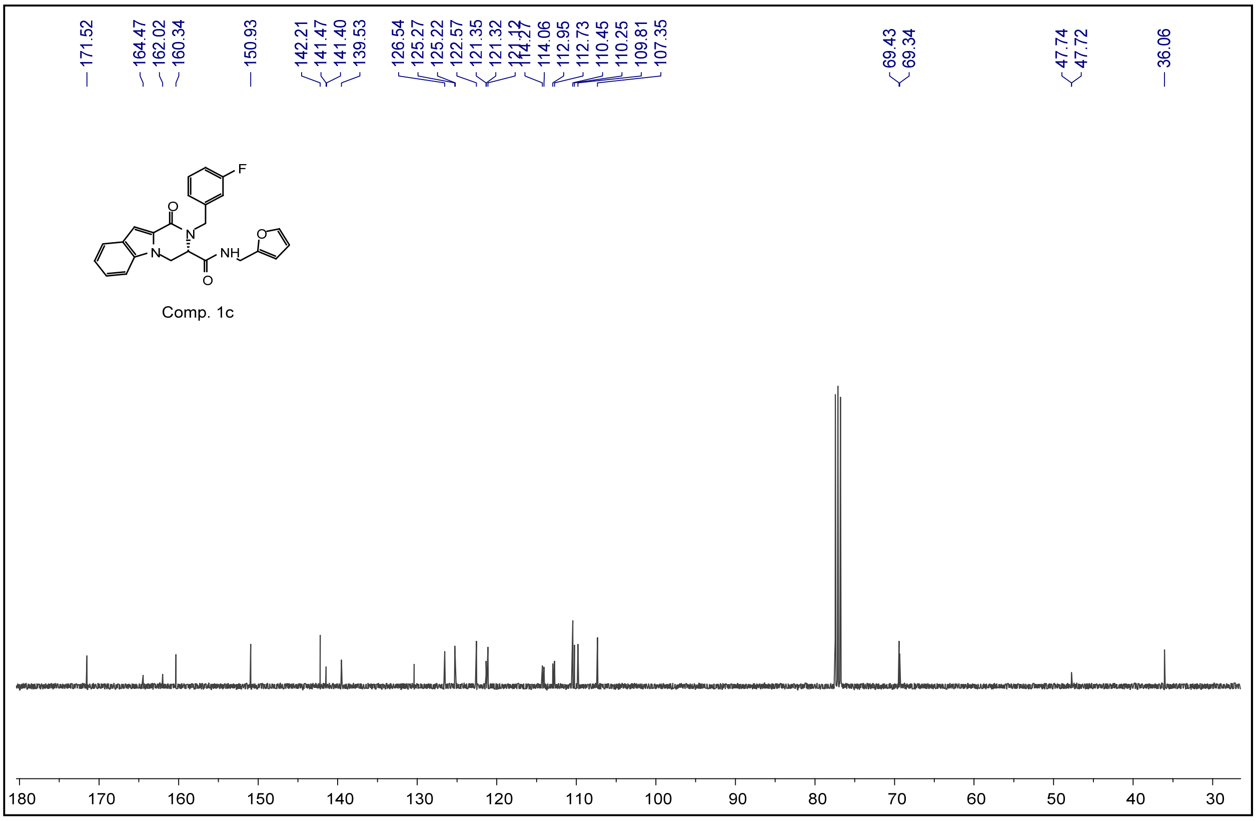

^{1}H NMR: (*S*)-*N*-(Furan-2-ylmethyl)-2-(4-fluorobenzyl)-1-oxo-1,2,3,4-tetrahydropyrazino[1,2-*a*]indole-3-carboxamide (1d)

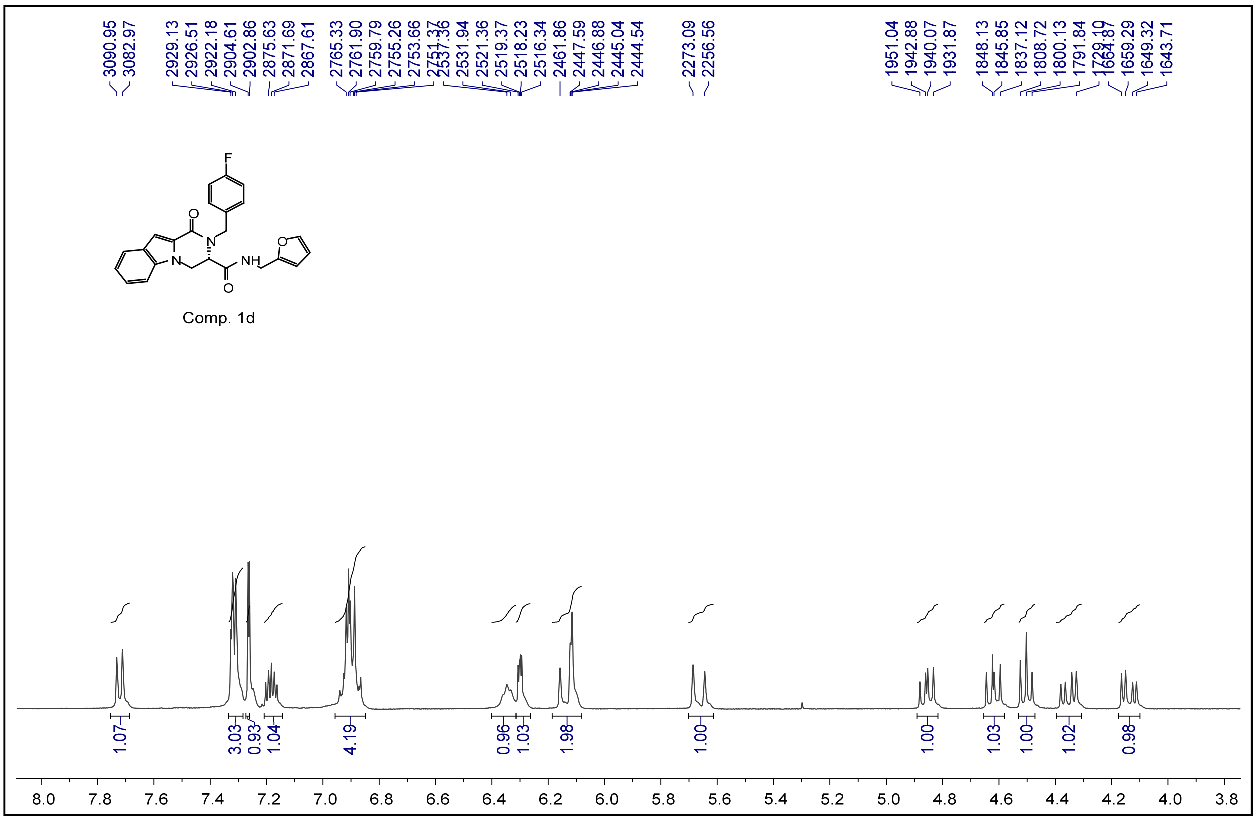

^{13}C NMR: (*S*)-*N*-(Furan-2-ylmethyl)-2-(4-fluorobenzyl)-1-oxo-1,2,3,4-tetrahydropyrazino[1,2-*a*]indole-3-carboxamide (1d)

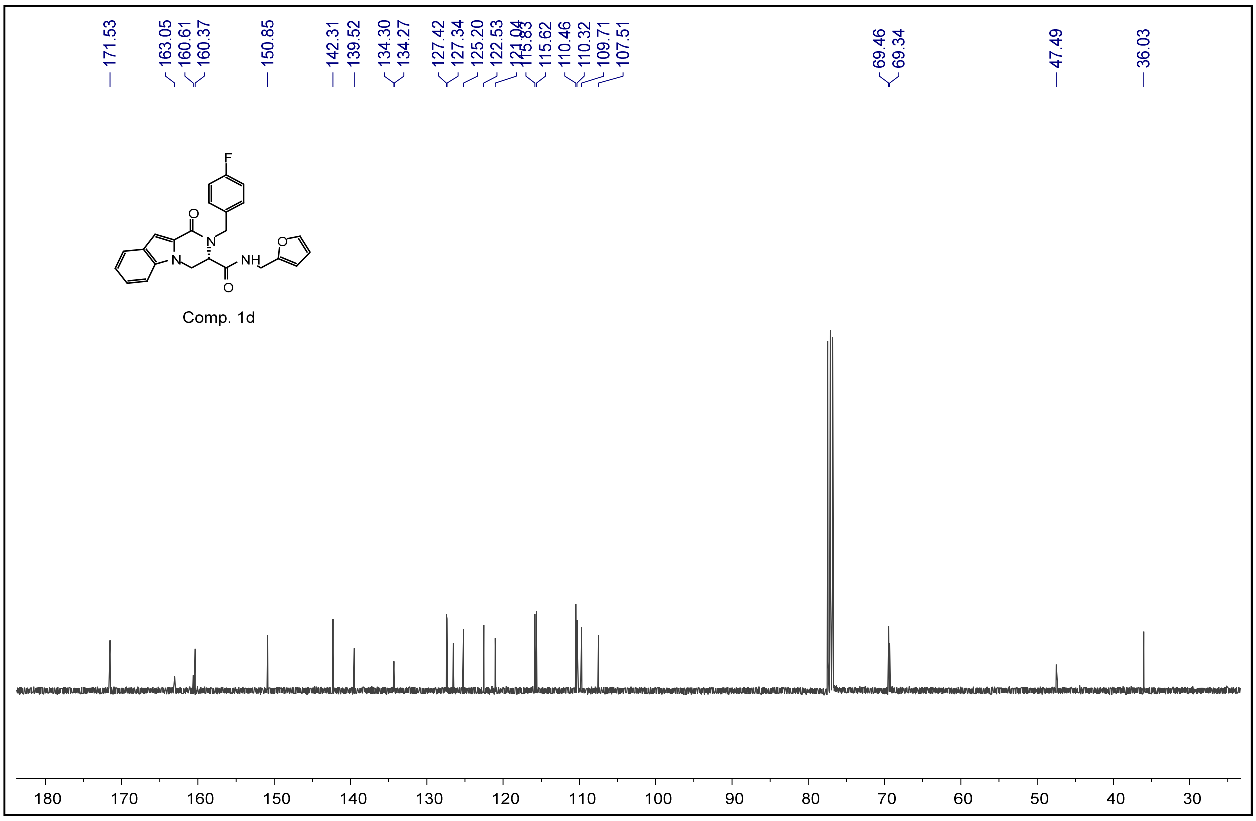

^{1}H NMR: (*S*)-*N*-(Furan-2-ylmethyl)-2-(3-cyanobenzyl)-1-oxo-1,2,3,4-tetrahydropyrazino[1,2-*a*]indole-3-carboxamide (1e)

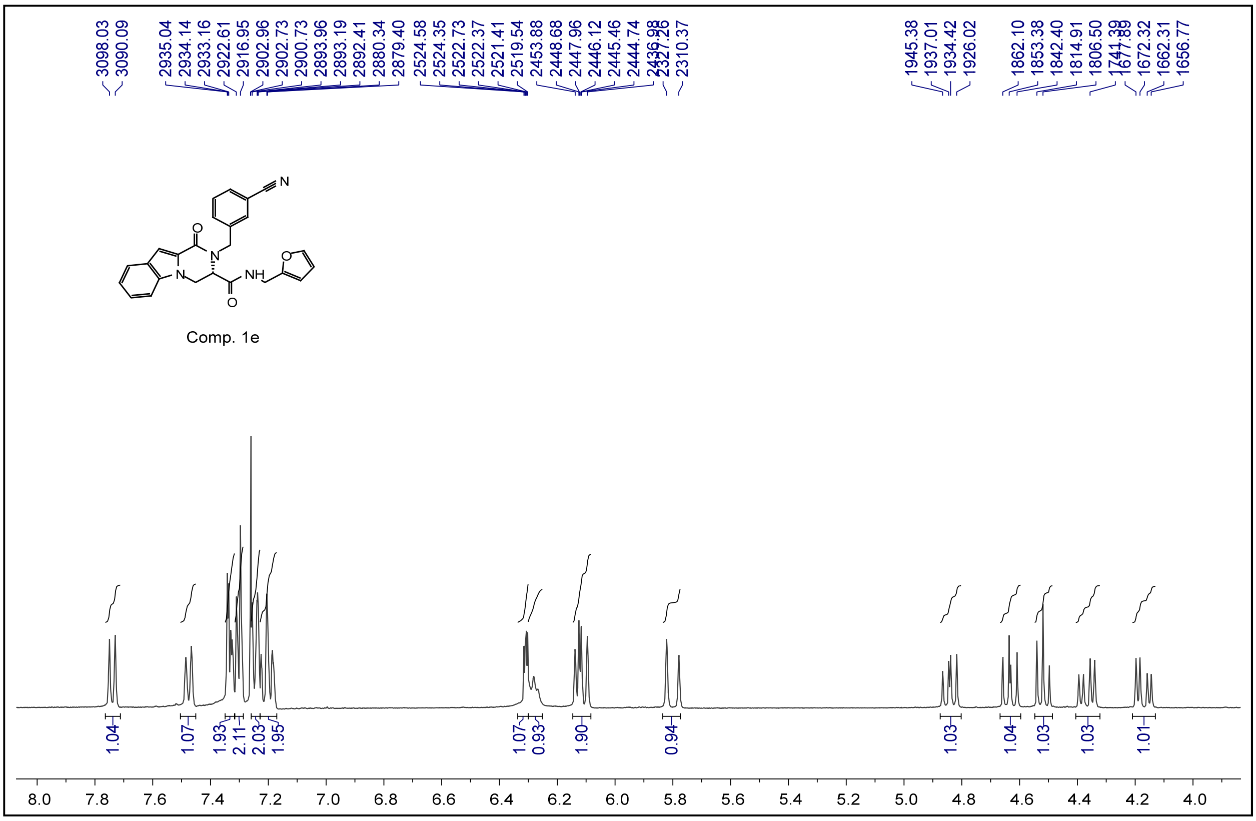

^{13}C NMR: (*S*)-*N*-(Furan-2-ylmethyl)-2-(3-cyanobenzyl)-1-oxo-1,2,3,4-tetrahydropyrazino[1,2-*a*]indole-3-carboxamide (1e)

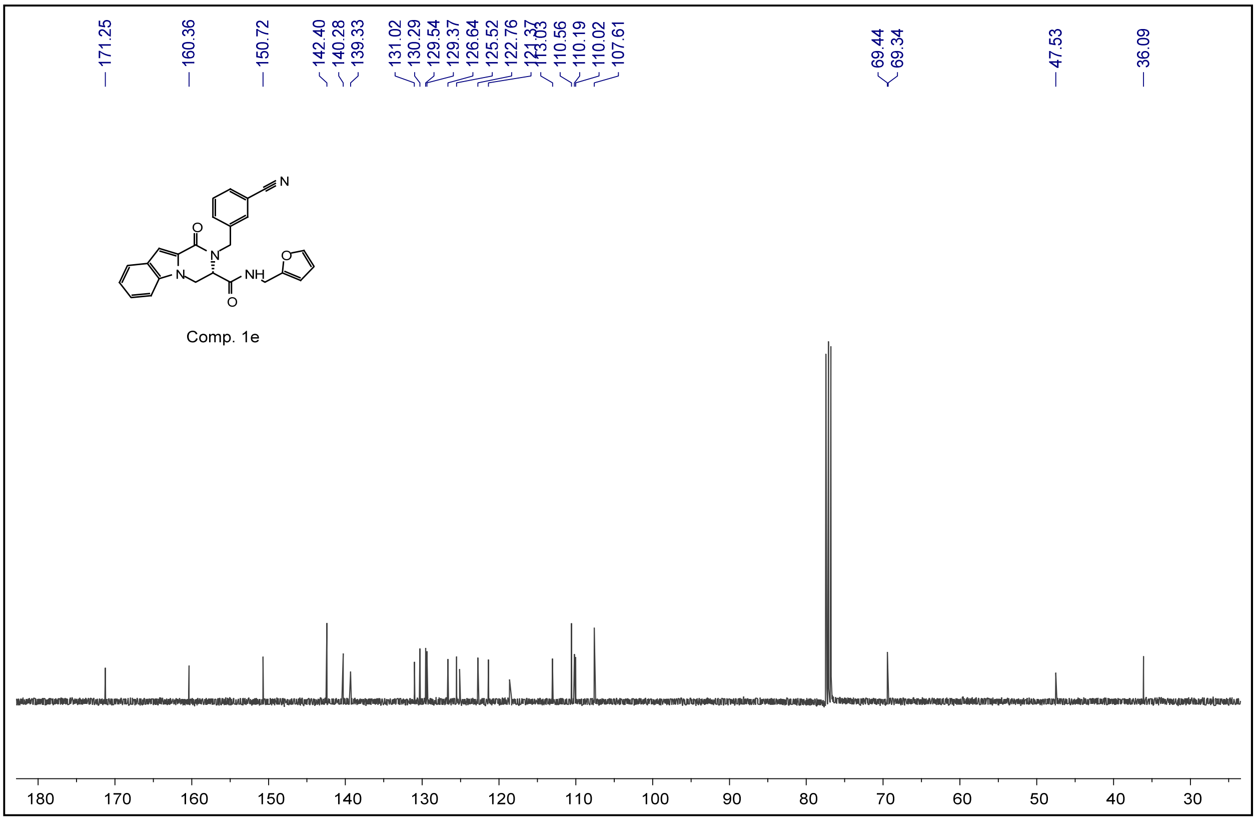

^{1}H NMR: (*S*)-*N*-(Furan-2-ylmethyl)-2-(4-cyanobenzyl)-1-oxo-1,2,3,4-tetrahydropyrazino[1,2-*a*]indole-3-carboxamide (1f)

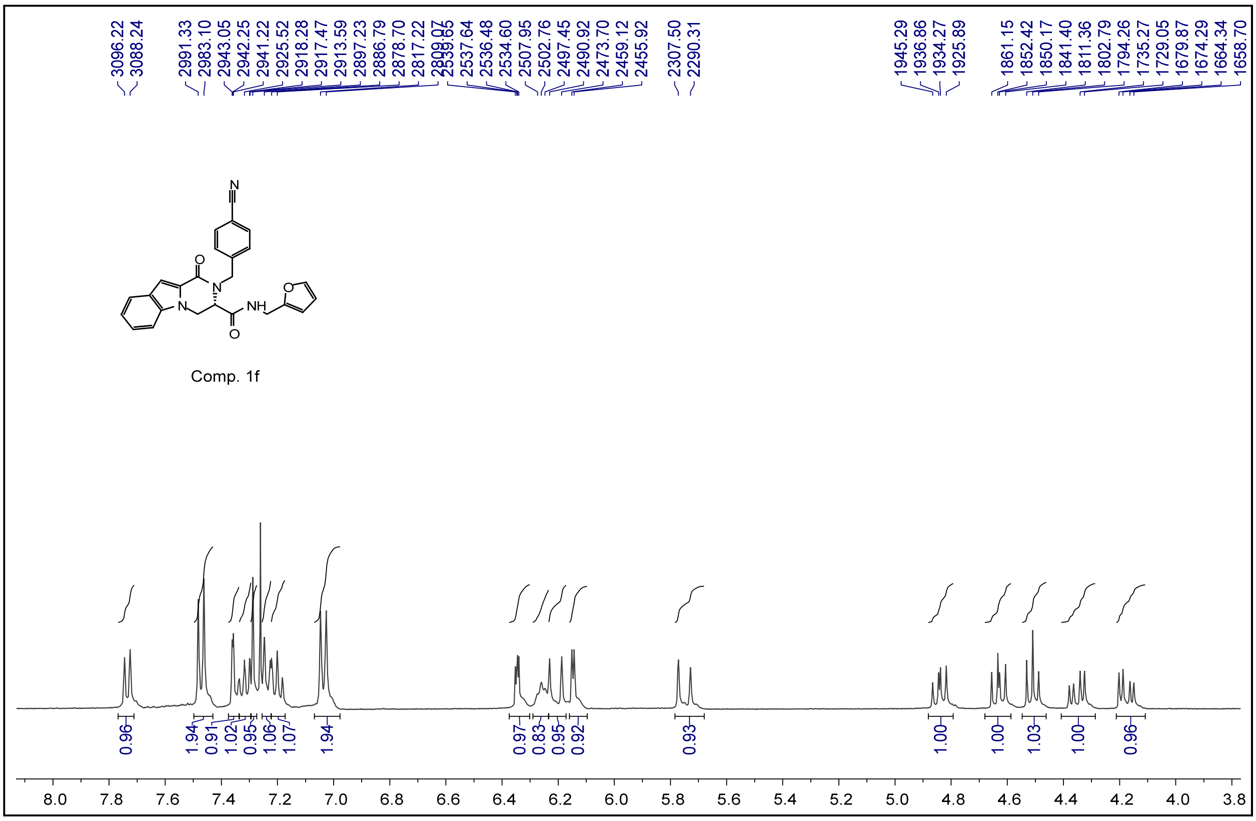

^{13}C NMR: (*S*)-*N*-(Furan-2-ylmethyl)-2-(4-cyanobenzyl)-1-oxo-1,2,3,4-tetrahydropyrazino[1,2-*a*]indole-3-carboxamide (1f)

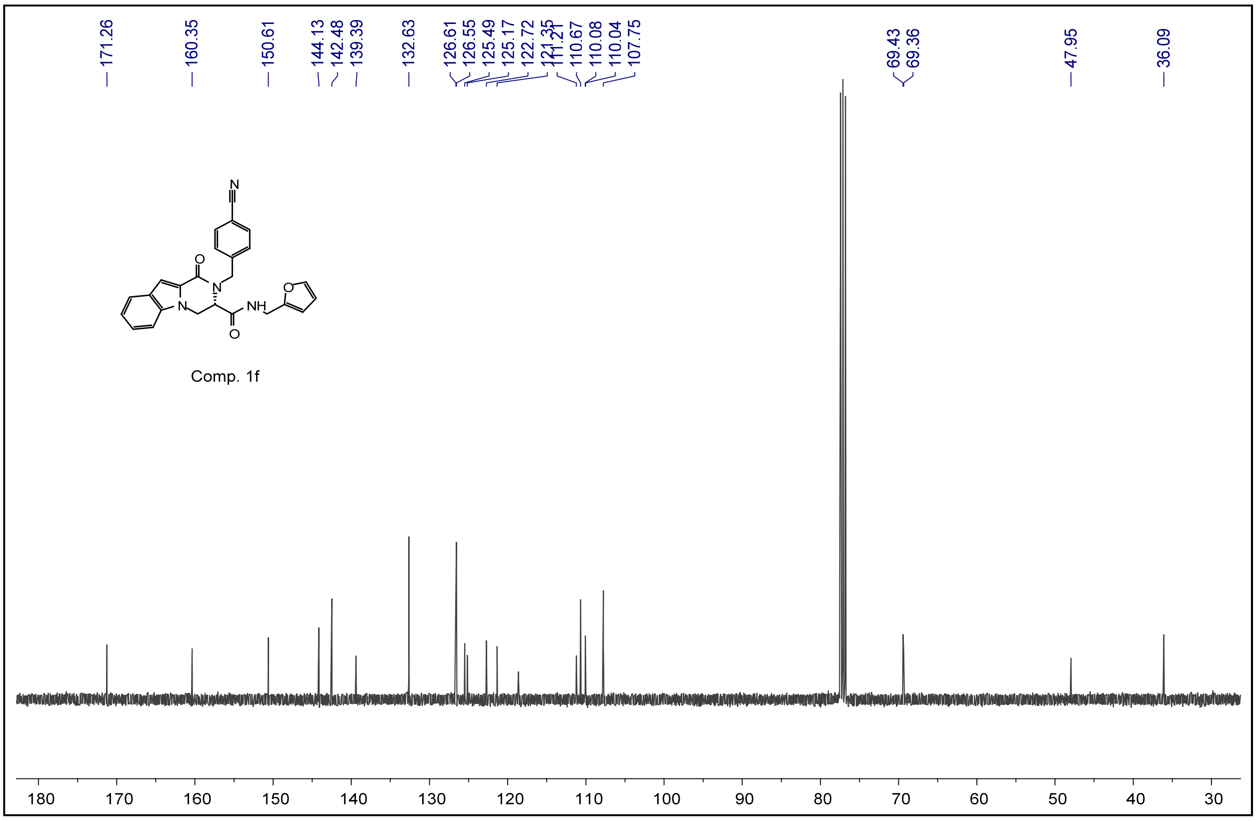

^{1}H NMR: (*S*)-*N*-(Furan-2-ylmethyl)-2-(4-trifluoromethylbenzyl)-1-oxo-1,2,3,4-tetrahydropyrazino[1,2-*a*]indole-3-carboxamide (1g)

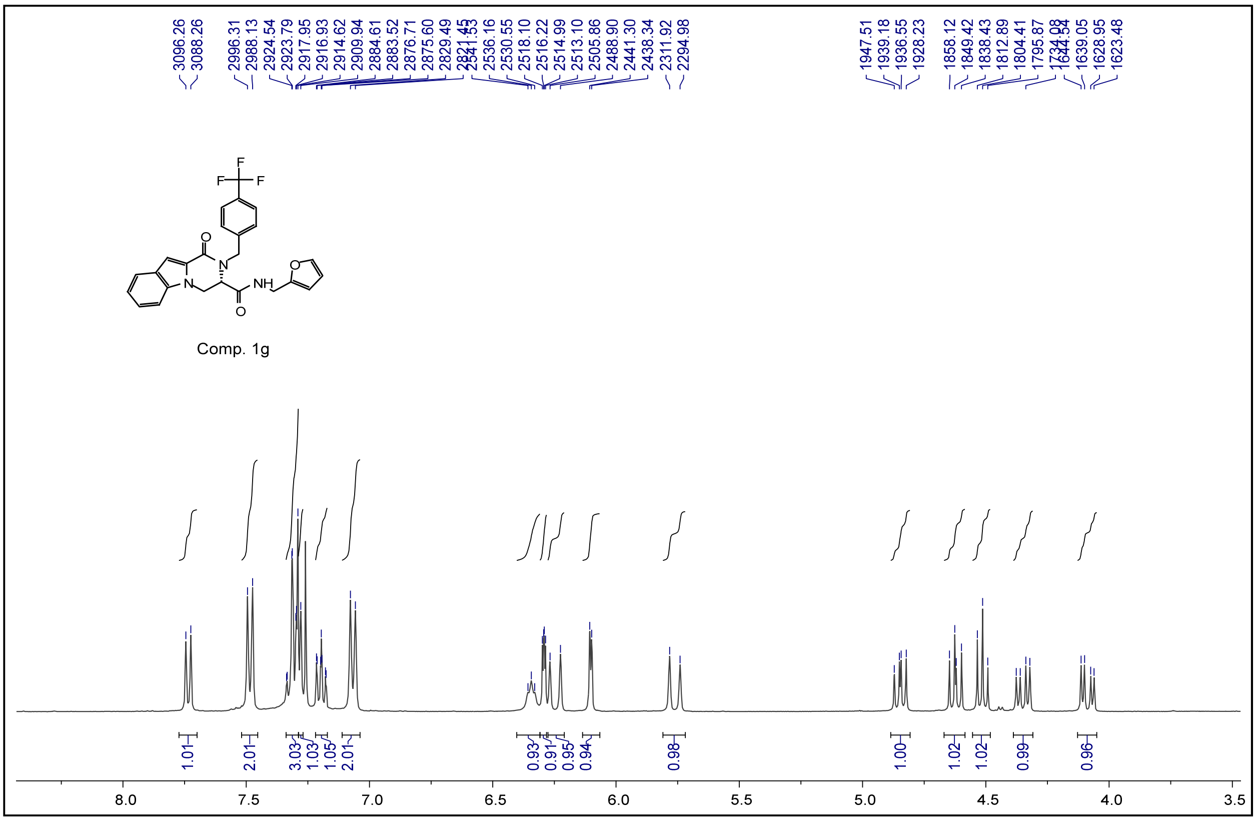

^{13}C NMR: (*S*)-*N*-(Furan-2-ylmethyl)-2-(4-trifluoromethylbenzyl)-1-oxo-1,2,3,4-tetrahydropyrazino[1,2-*a*]indole-3-carboxamide (1g)

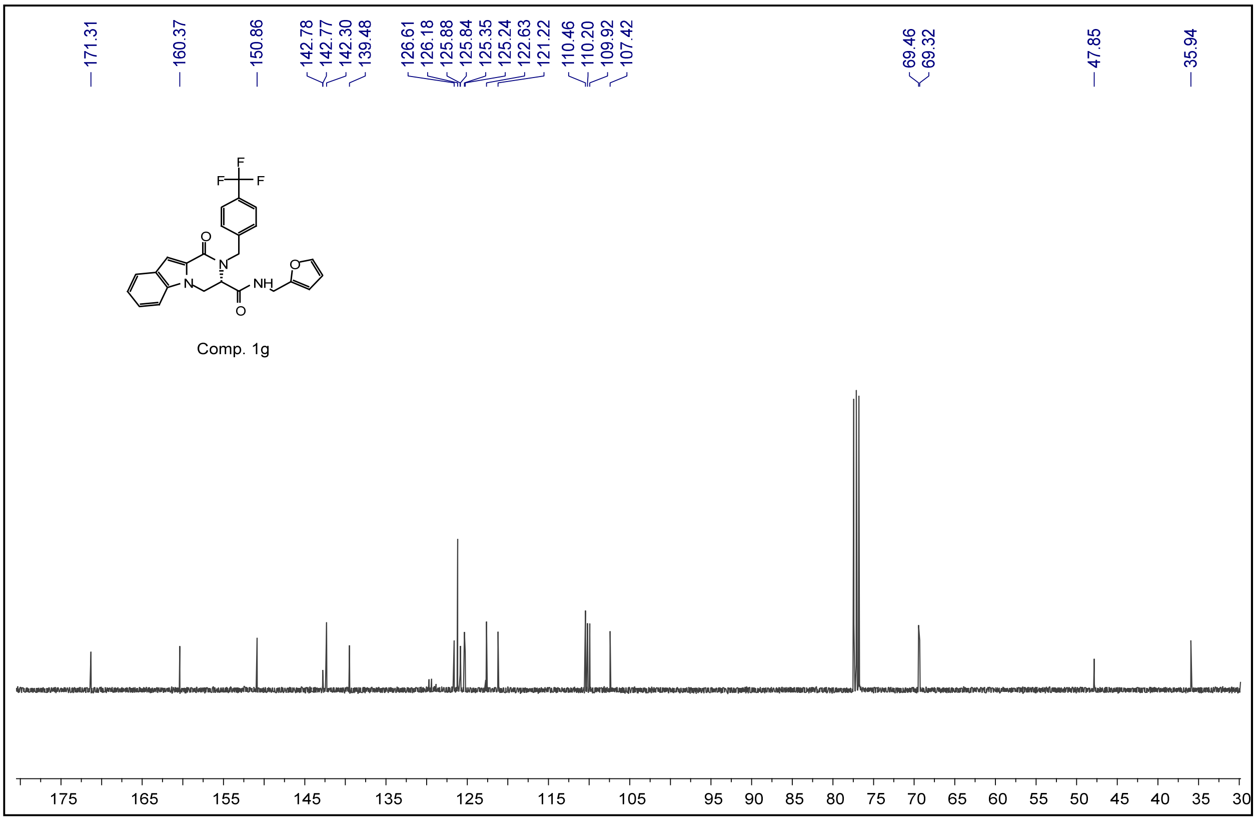

^{1}H NMR: (***S***)-*N*-(Furan-2-ylmethyl)-2-(3-trifluoromethoxybenzyl)-1-oxo-1,2,3,4-tetrahydropyrazino[1,2-*a*]indole-3-carboxamide (1h)

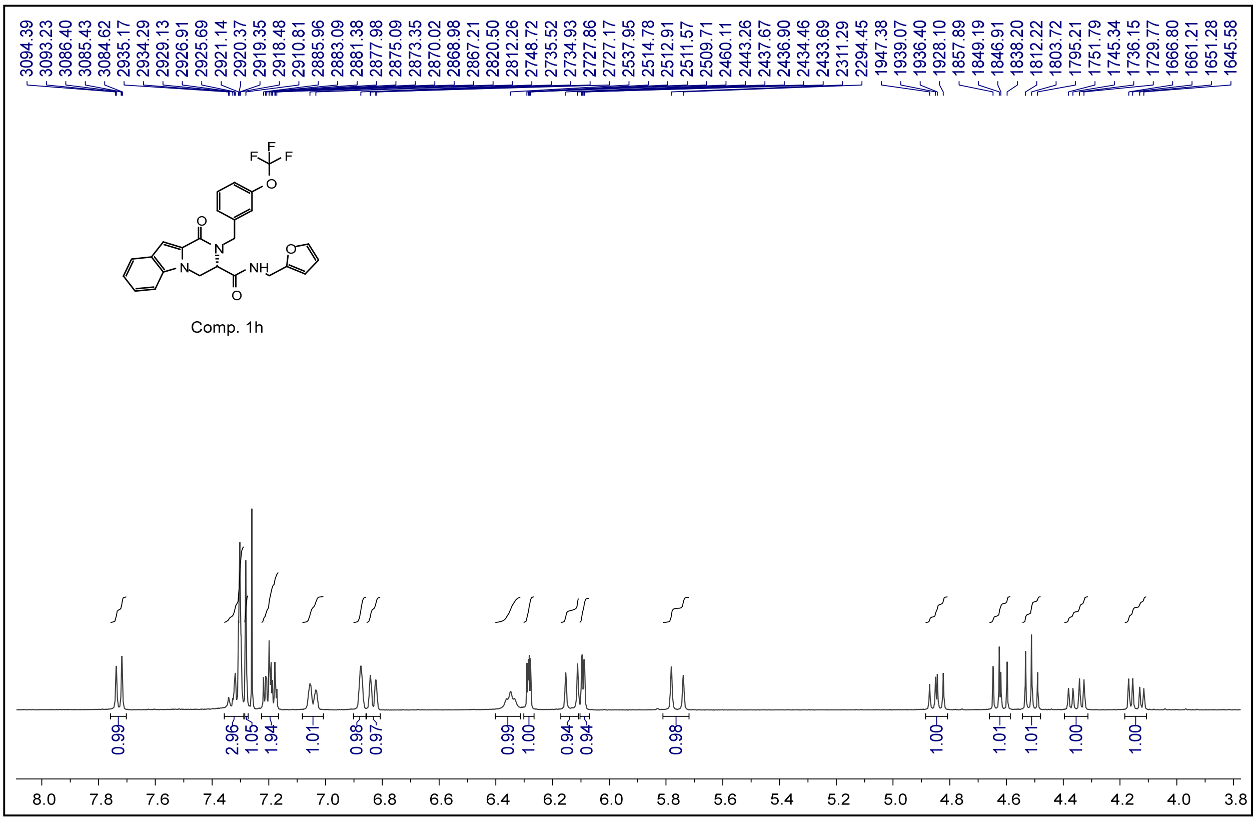

^{13}C NMR: (***S***)-*N*-(Furan-2-ylmethyl)-2-(3-trifluoromethoxybenzyl)-1-oxo-1,2,3,4-tetrahydropyrazino[1,2-*a*]indole-3-carboxamide (1h)

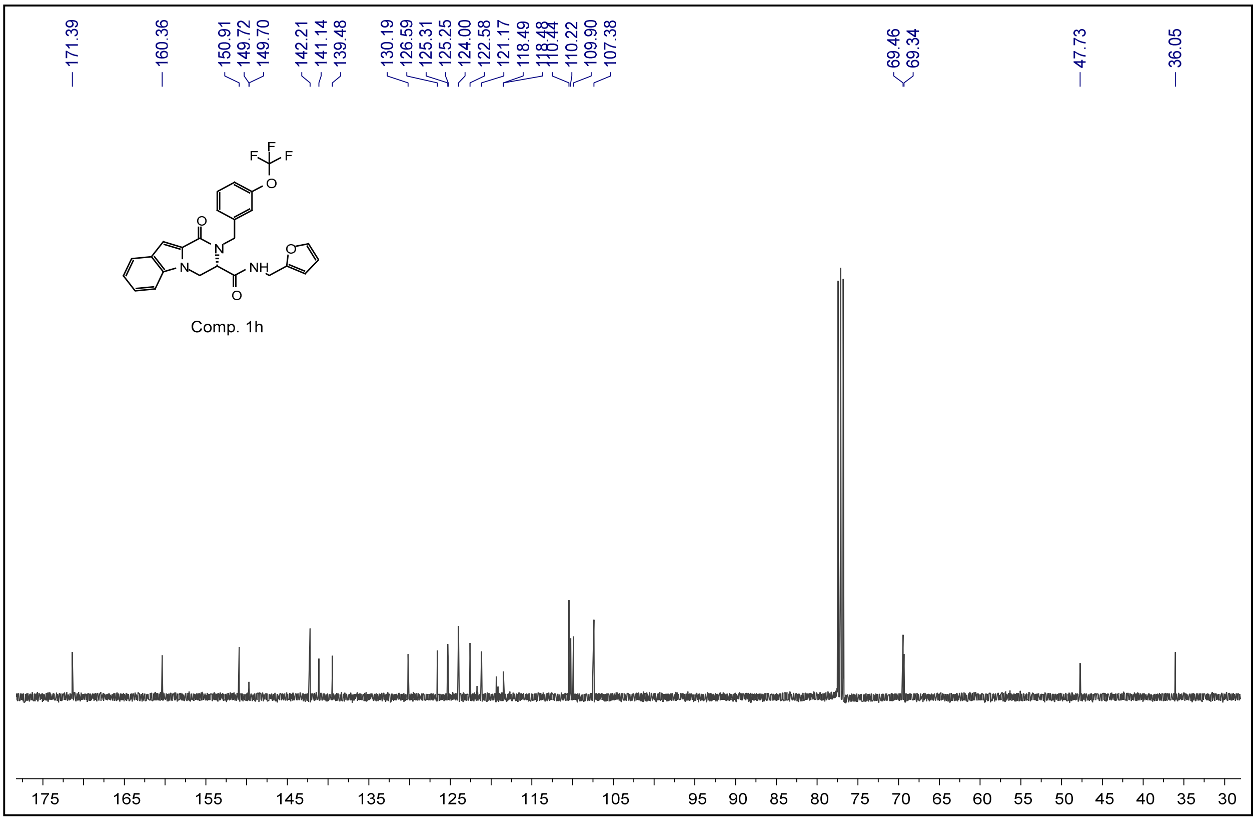

^{1}H NMR: (*S*)-*N*-(Furan-2-ylmethyl)-2-(4-trifluoromethoxybenzyl)-1-oxo-1,2,3,4-tetrahydropyrazino[1,2-*a*]indole-3-carboxamide (1i)

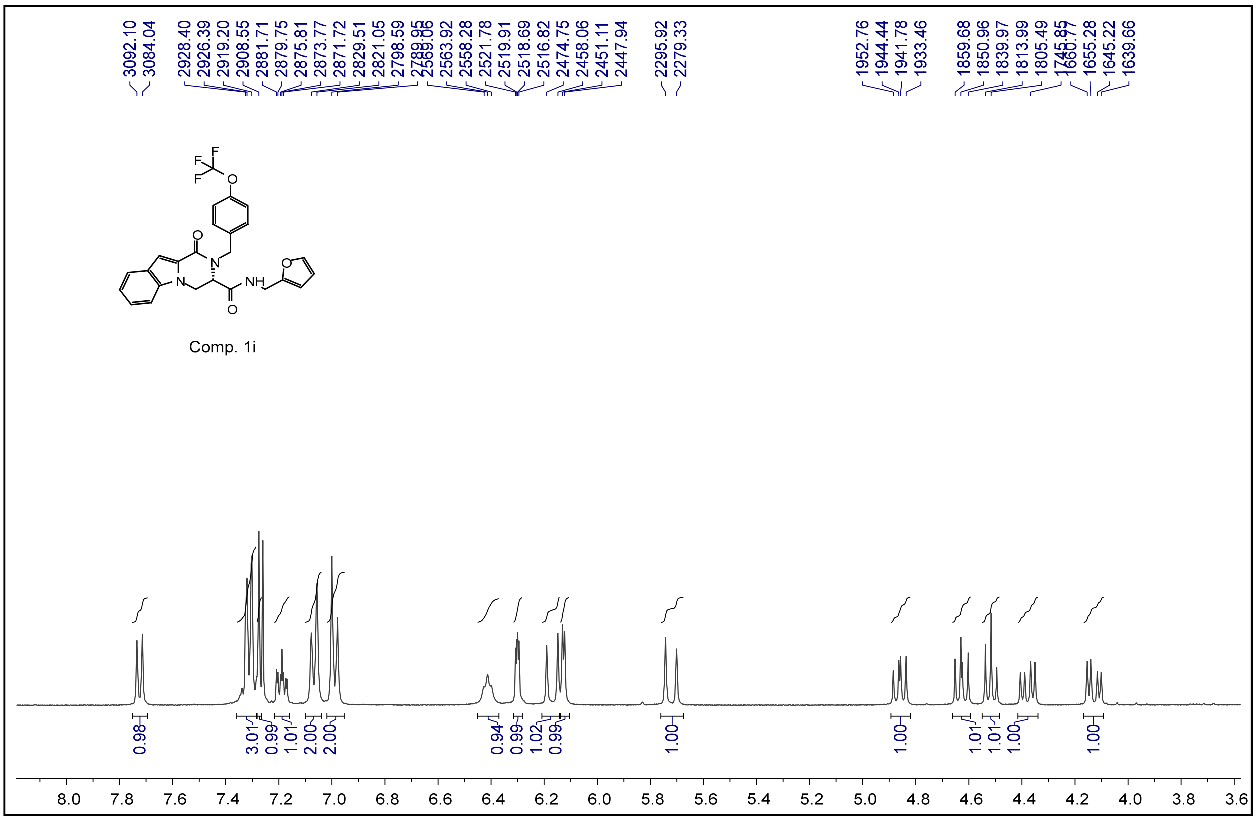

^{13}C NMR: (*S*)-*N*-(Furan-2-ylmethyl)-2-(4-trifluoromethoxybenzyl)-1-oxo-1,2,3,4-tetrahydropyrazino[1,2-*a*]indole-3-carboxamide (1i)

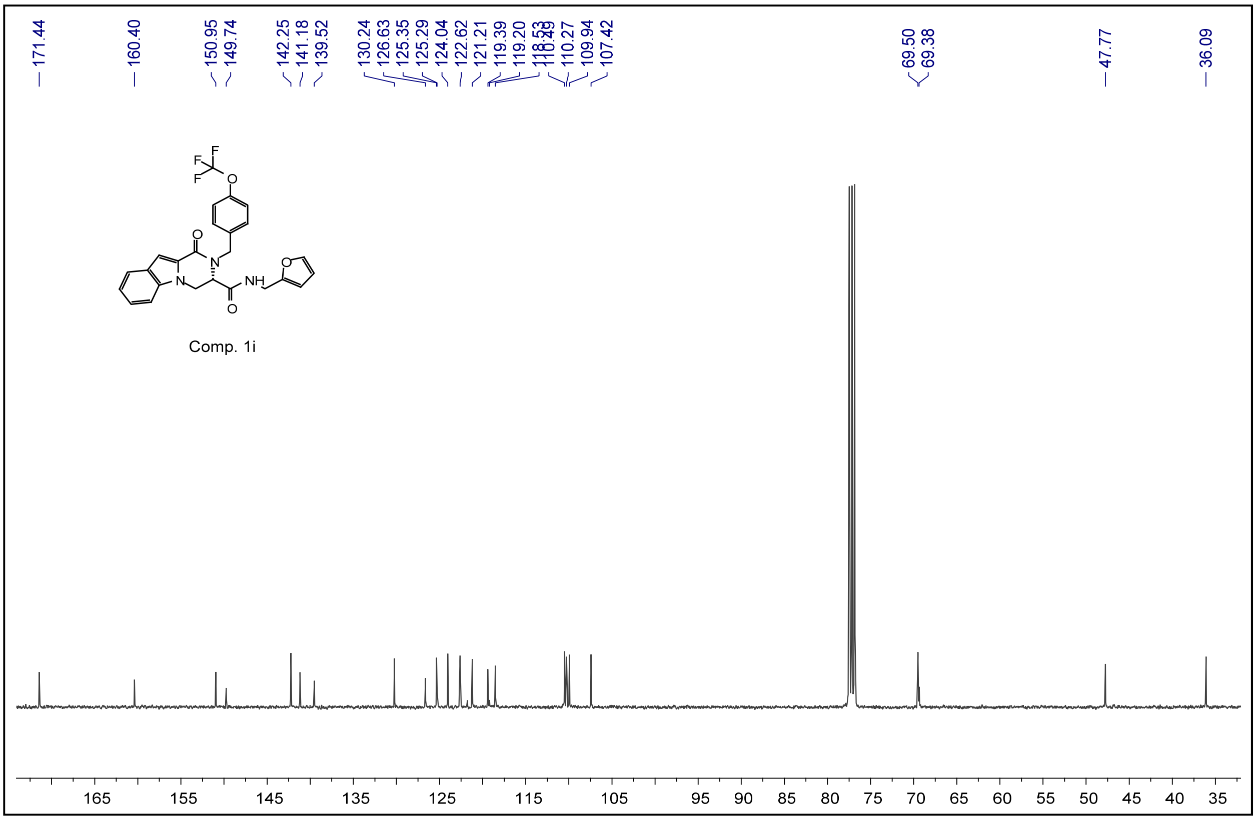

^{1}H NMR: (*S*)-*N*-(Furan-2-ylmethyl)-2-(3-methylbenzyl)-1-oxo-1,2,3,4-tetrahydropyrazino[1,2-*a*]indole-3-carboxamide (1j)

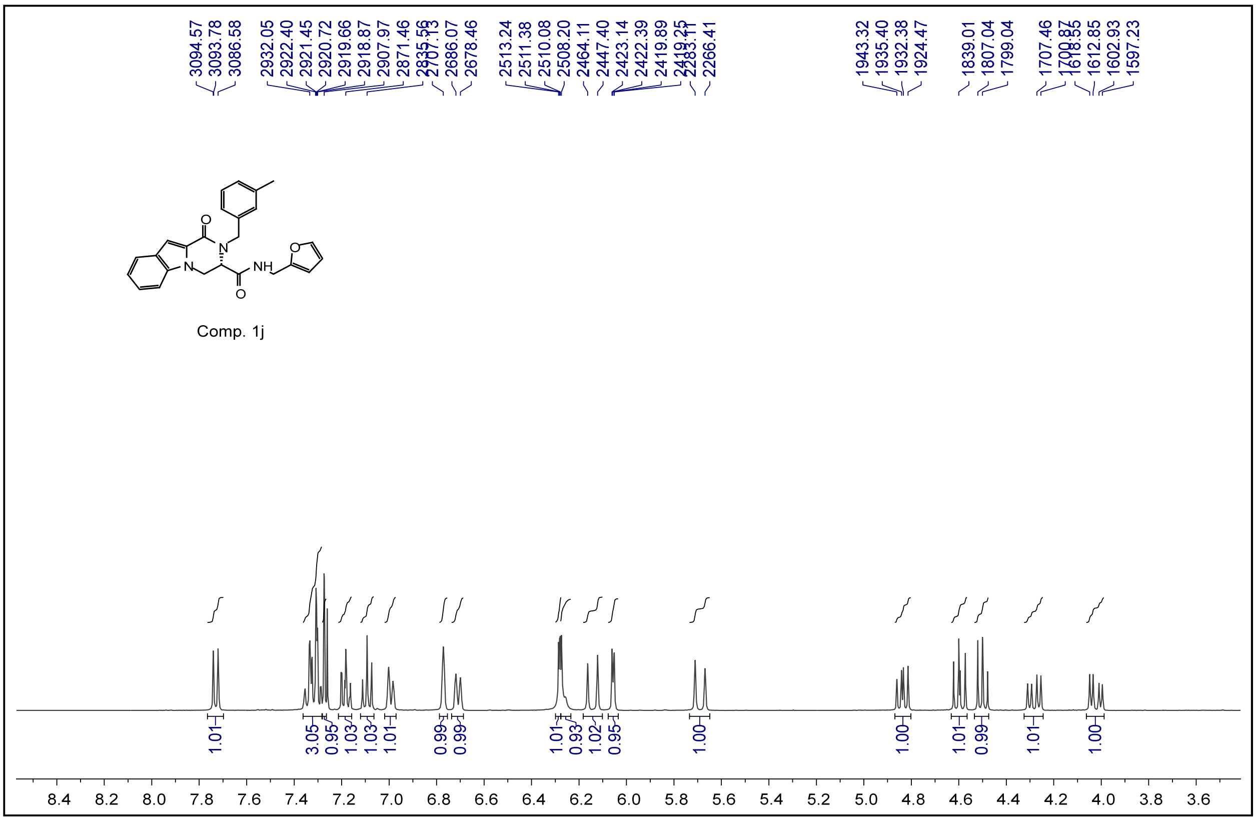

^{13}C NMR: (*S*)-*N*-(Furan-2-ylmethyl)-2-(3-methylbenzyl)-1-oxo-1,2,3,4-tetrahydropyrazino[1,2-*a*]indole-3-carboxamide (1j)

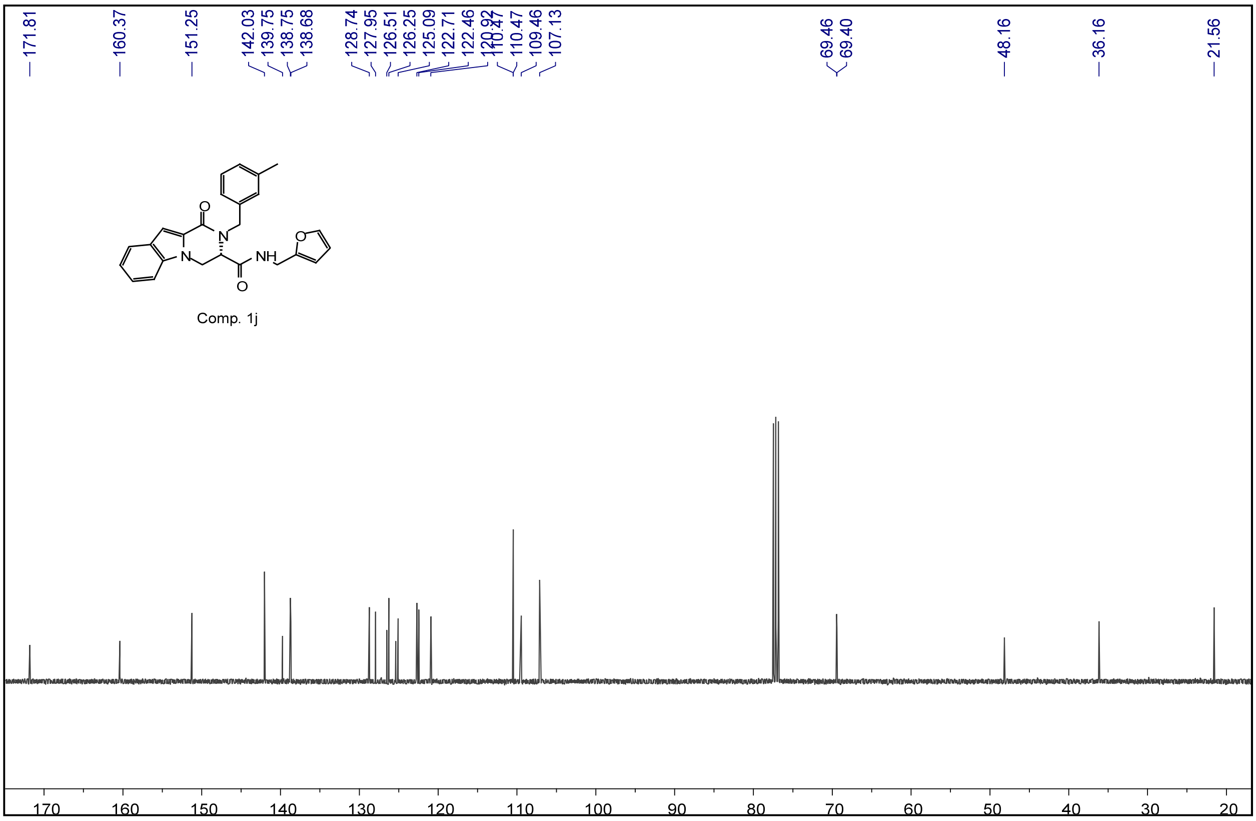

^{1}H NMR: (*S*)-*N*-(Furan-2-ylmethyl)-2-(4-methylbenzyl)-1-oxo-1,2,3,4-tetrahydropyrazino[1,2-*a*]indole-3-carboxamide (1k)

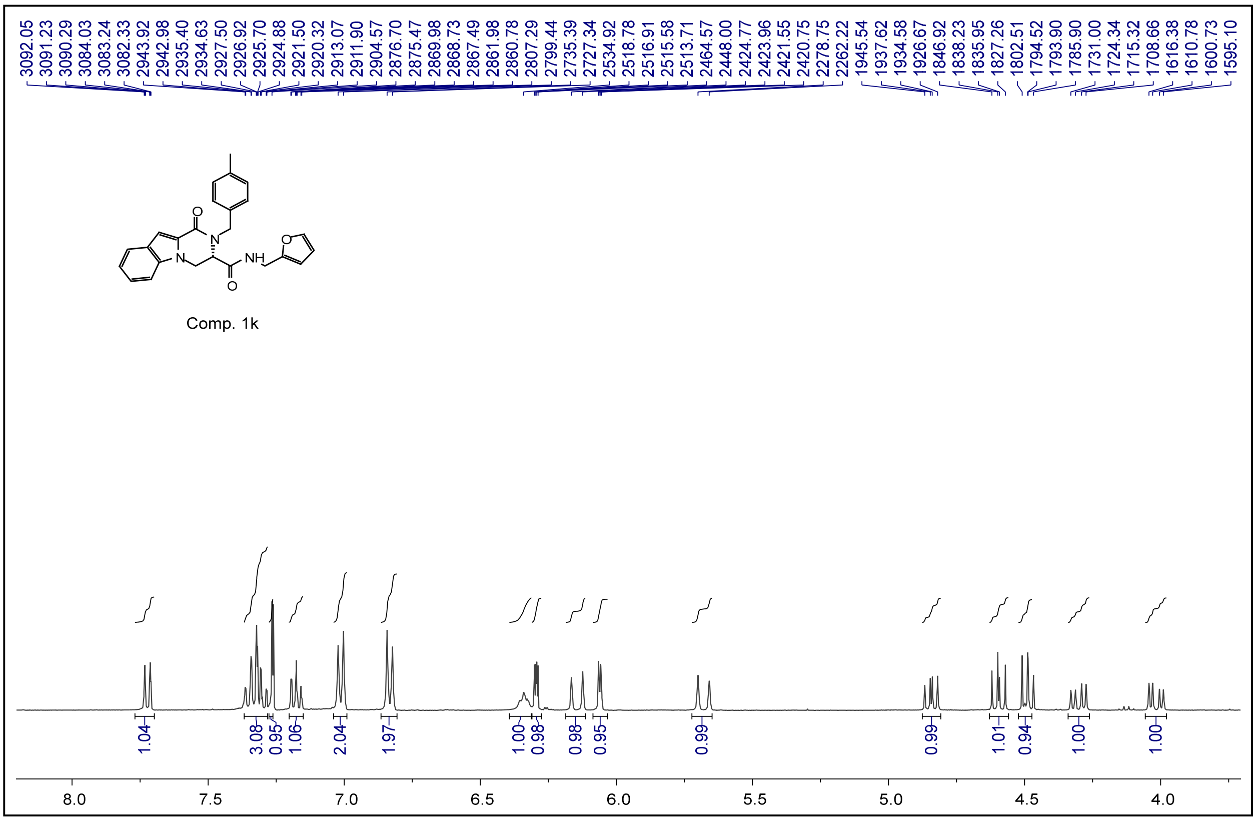

^{13}C NMR: (*S*)-*N*-(Furan-2-ylmethyl)-2-(4-methylbenzyl)-1-oxo-1,2,3,4-tetrahydropyrazino[1,2-*a*]indole-3-carboxamide (1k)

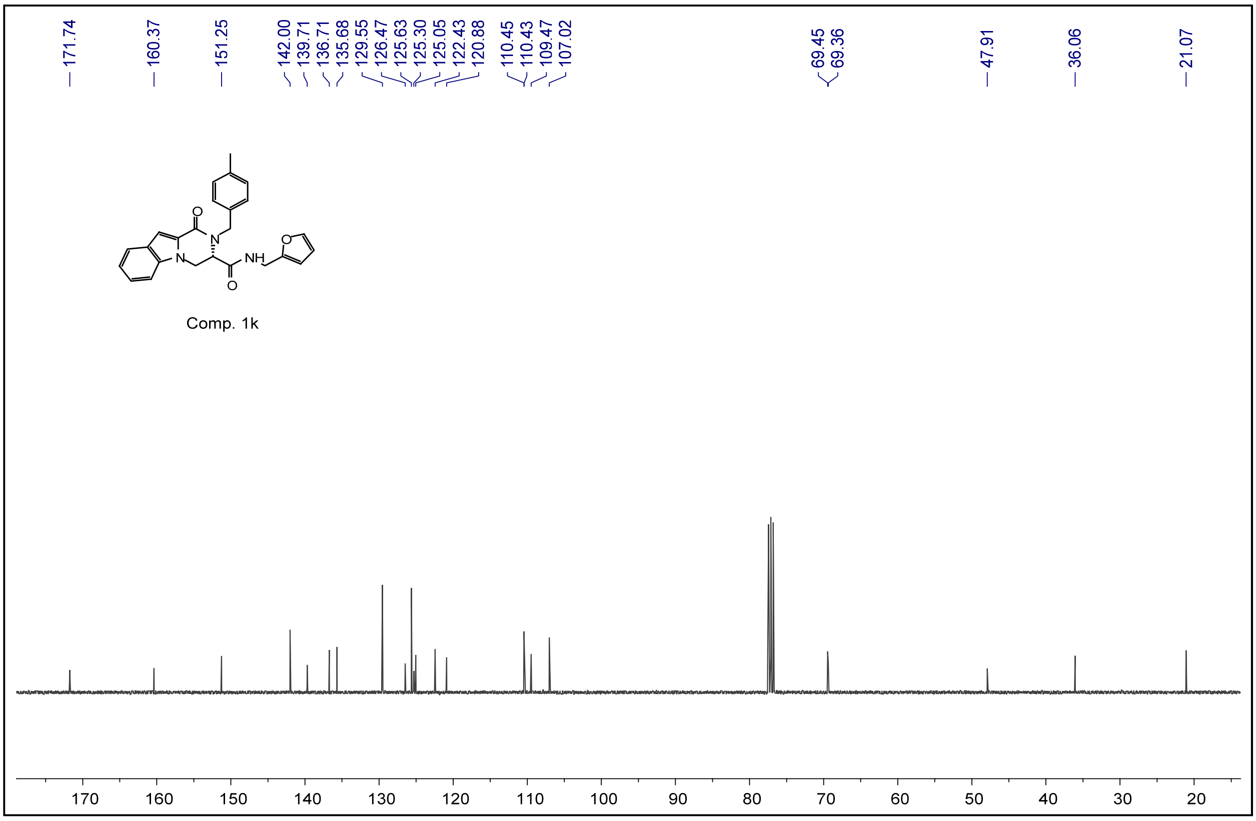

^{1}H NMR: (*S*)-*N*-(Furan-2-ylmethyl)-2-(naphthalen-1-ylmethyl)-1-oxo-1,2,3,4-tetrahydropyrazino[1,2-*a*]indole-3-carboxamide (1l)

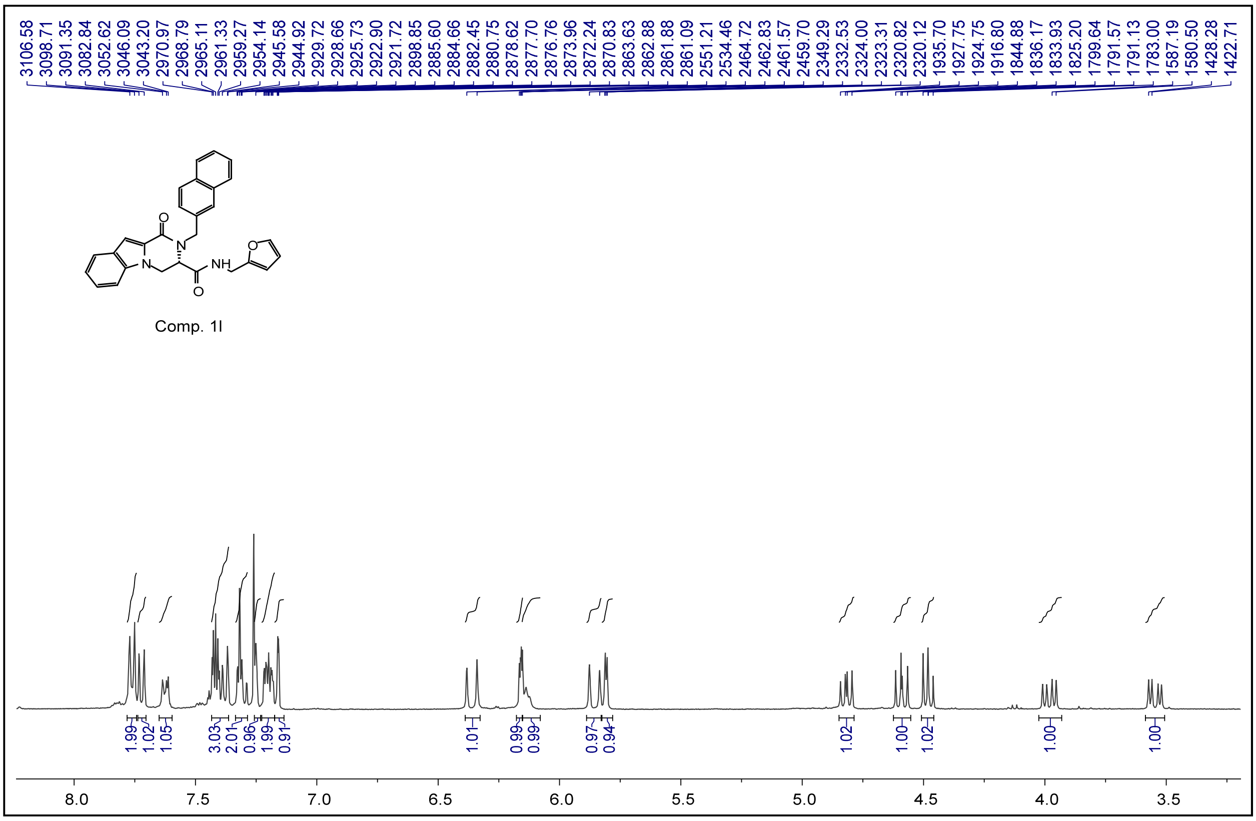

^{13}C NMR: (*S*)-*N*-(Furan-2-ylmethyl)-2-(naphthalen-1-ylmethyl)-1-oxo-1,2,3,4-tetrahydropyrazino[1,2-*a*]indole-3-carboxamide (1l)

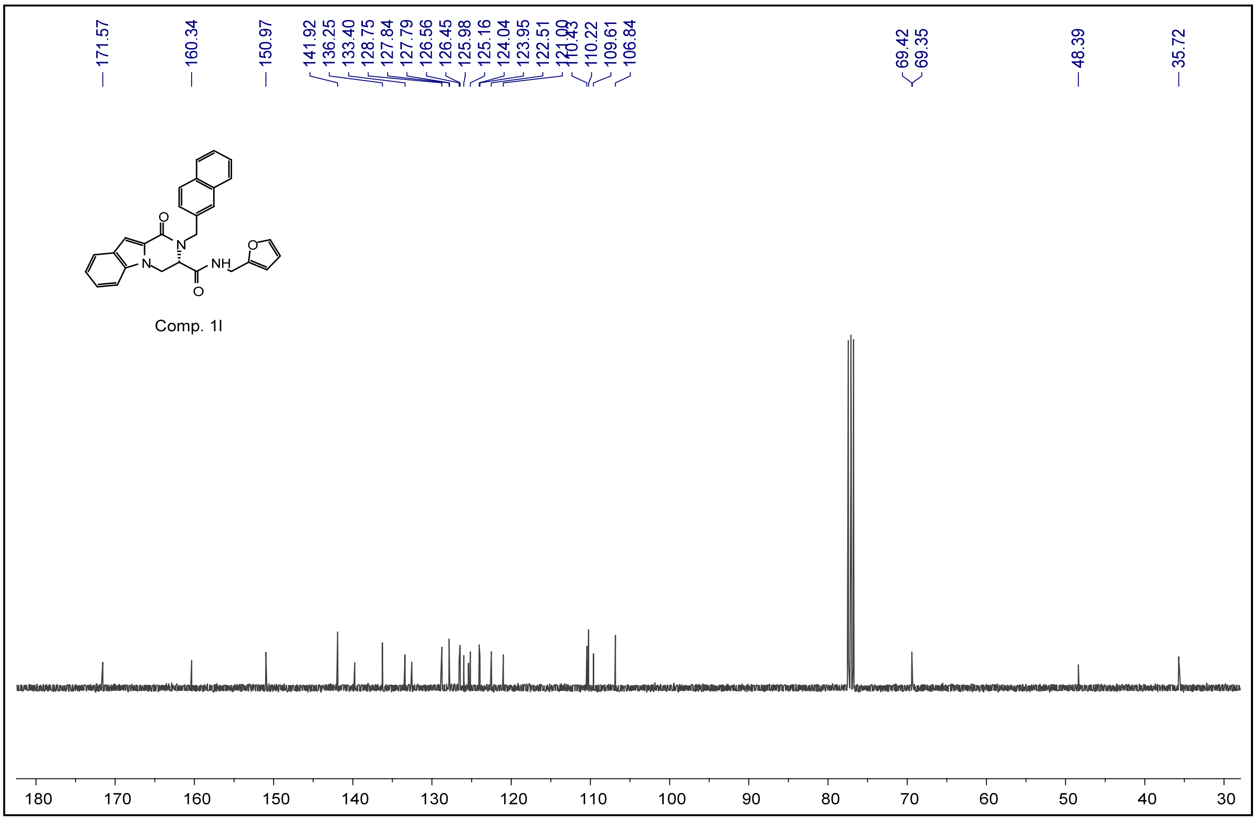

^{1}H NMR: (*S*)-*N*-(Furan-2-ylmethyl)-2-([1,1'-biphenyl]-4-ylmethyl)-1-oxo-1,2,3,4-tetrahydropyrazino[1,2-*a*]indole-3-carboxamide (1m)

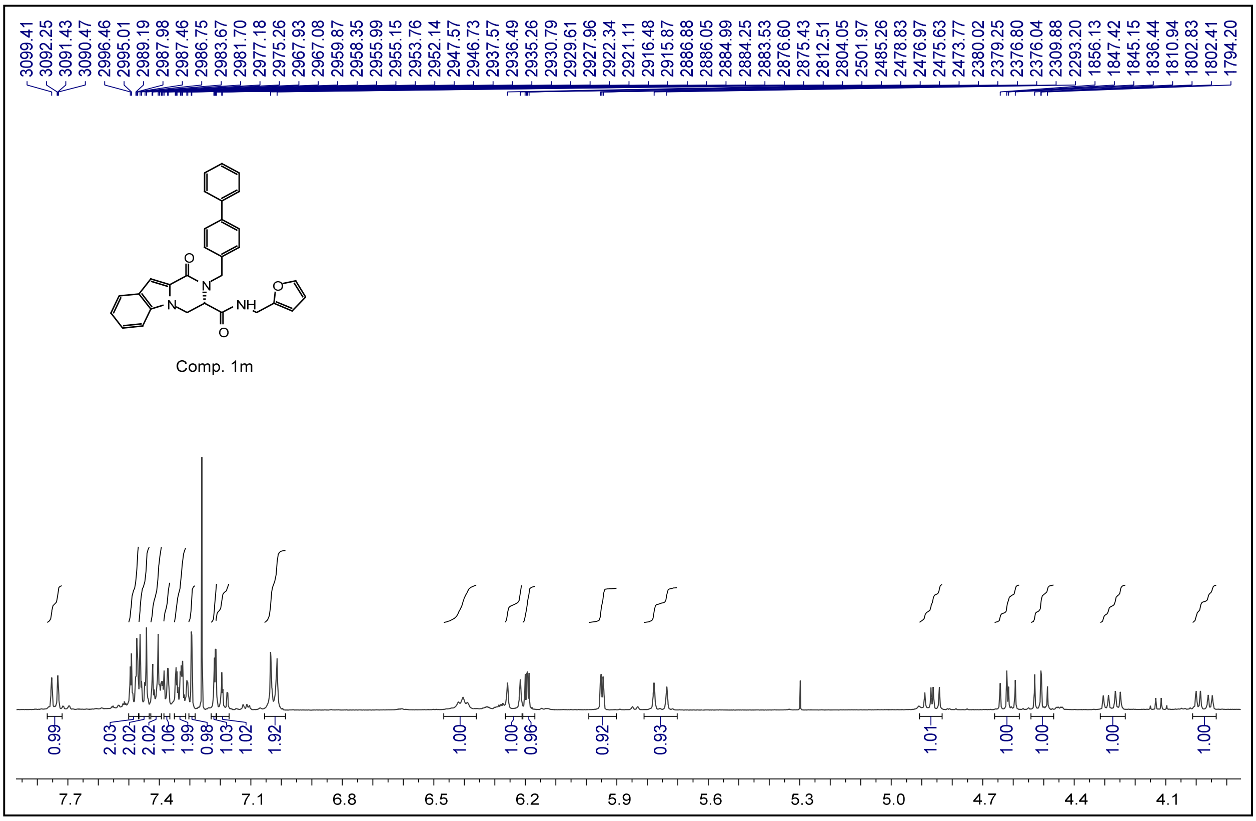

^{13}C NMR: (*S*)-*N*-(Furan-2-ylmethyl)-2-([1,1'-biphenyl]-4-ylmethyl)-1-oxo-1,2,3,4-tetrahydropyrazino[1,2-*a*]indole-3-carboxamide (1m)

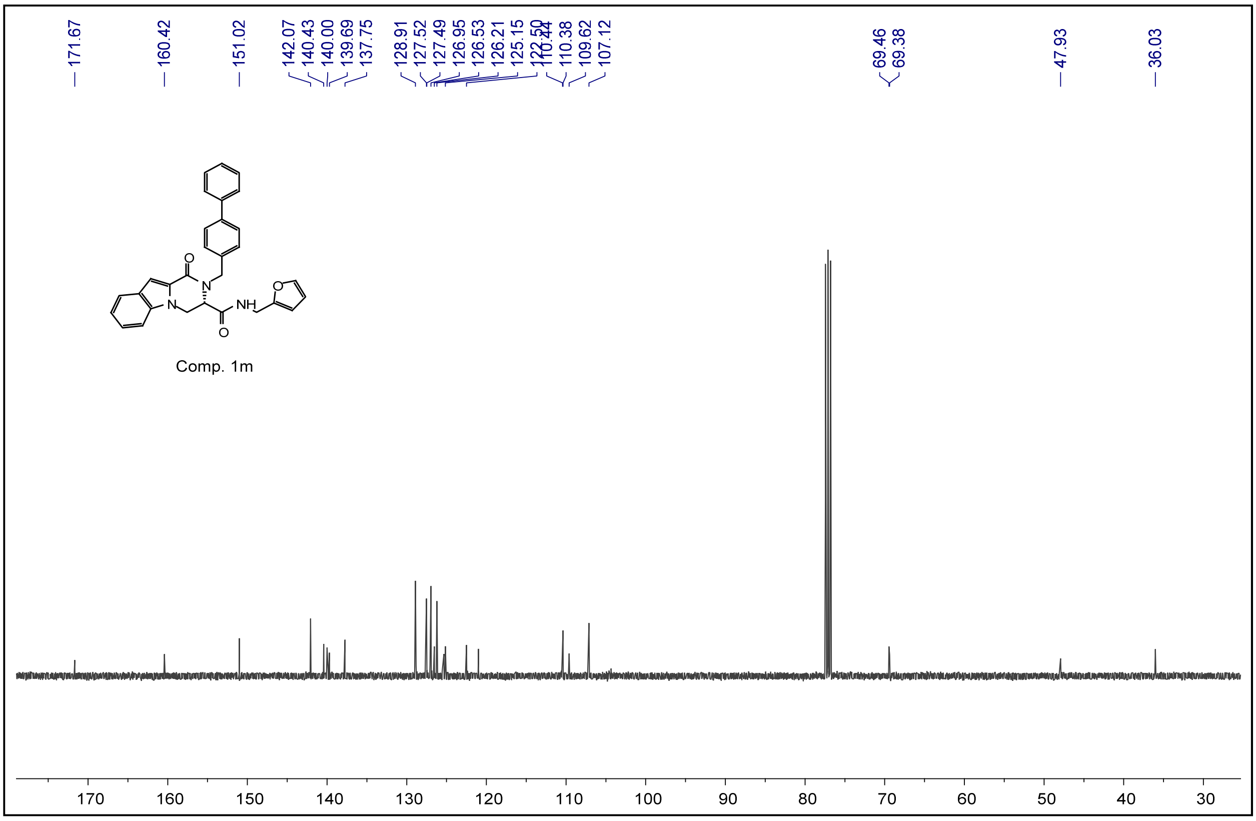

^{1}H NMR: (*S*)-*N*-Benzyl-2-(3-nitrobenzyl)-1-oxo-1,2,3,4-tetrahydropyrazino[1,2-*a*]indole-3-carboxamide (2a)

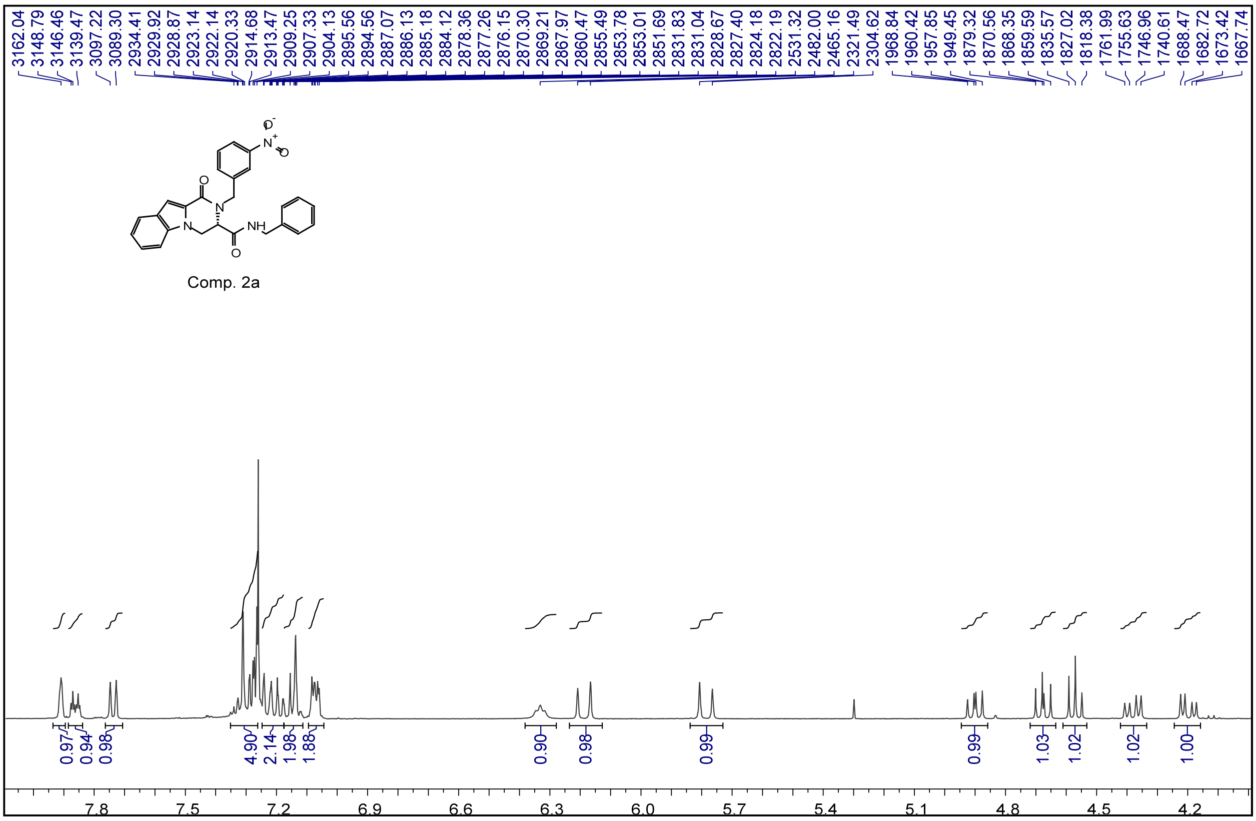

^{13}C NMR: (*S*)-*N*-Benzyl-2-(3-nitrobenzyl)-1-oxo-1,2,3,4-tetrahydropyrazino[1,2-*a*]indole-3-carboxamide (2a)

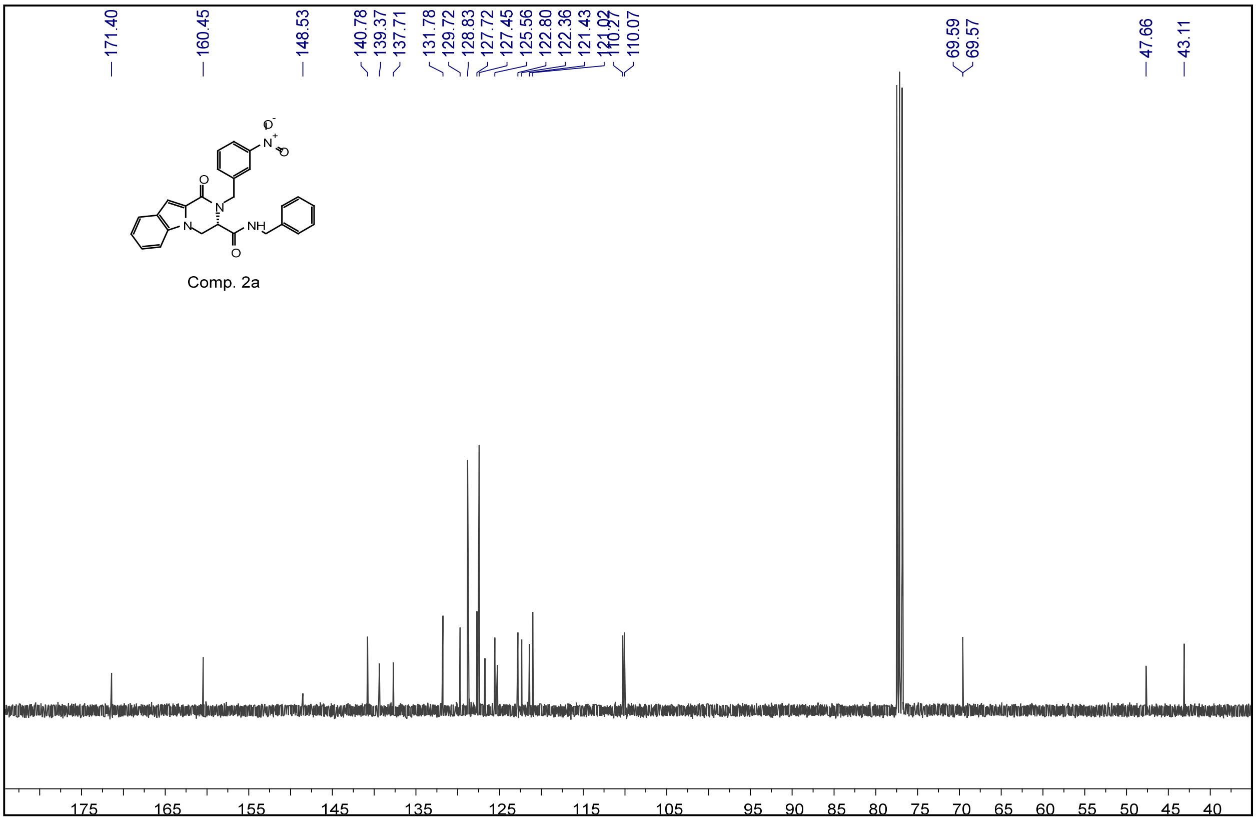

^{1}H NMR: (*S*)-*N*-Benzyl-2-(4-nitrobenzyl)-1-oxo-1,2,3,4-tetrahydropyrazino[1,2-*a*]indole-3-carboxamide (2b)

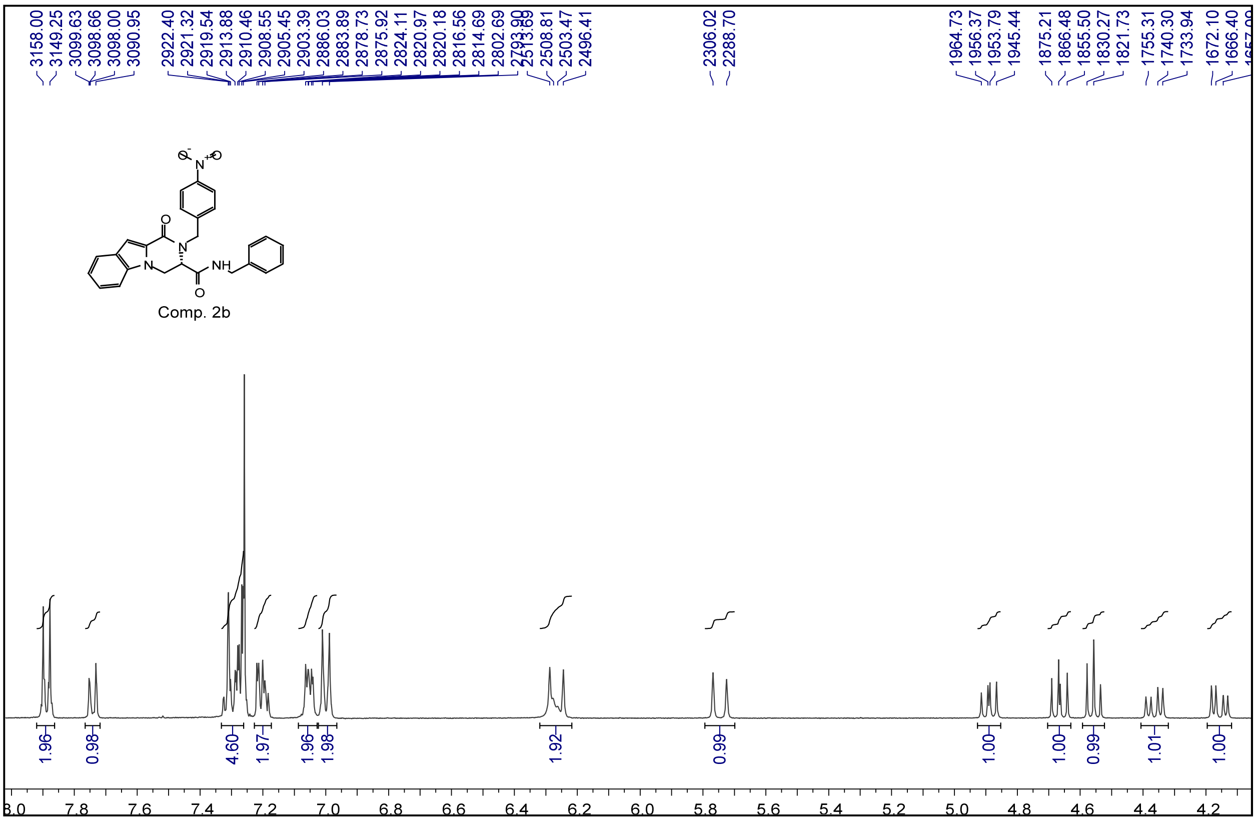

^{13}C NMR: (*S*)-*N*-Benzyl-2-(4-nitrobenzyl)-1-oxo-1,2,3,4-tetrahydropyrazino[1,2-*a*]indole-3-carboxamide (2b)

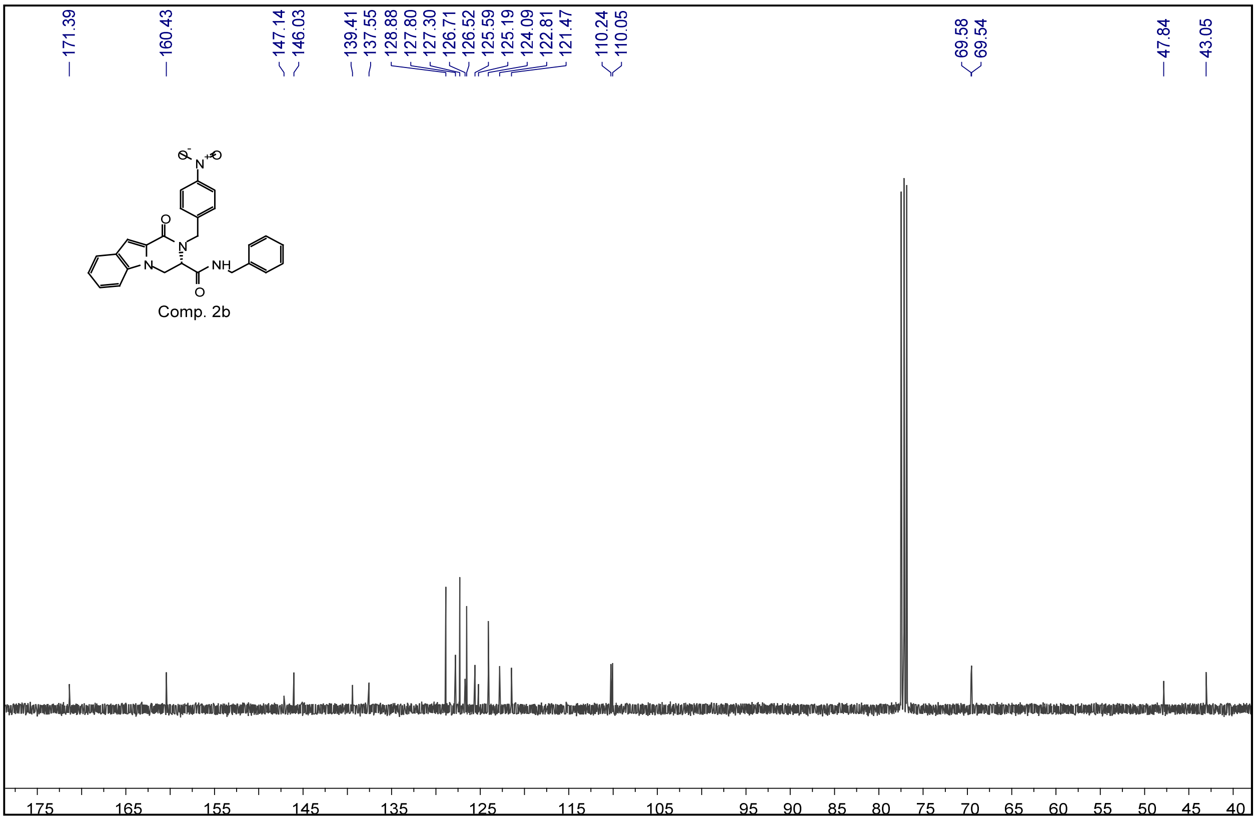

^{1}H NMR: (*S*)-*N*-Benzyl-2-(3-fluorobenzyl)-1-oxo-1,2,3,4-tetrahydropyrazino[1,2-*a*]indole-3-carboxamide (2c)

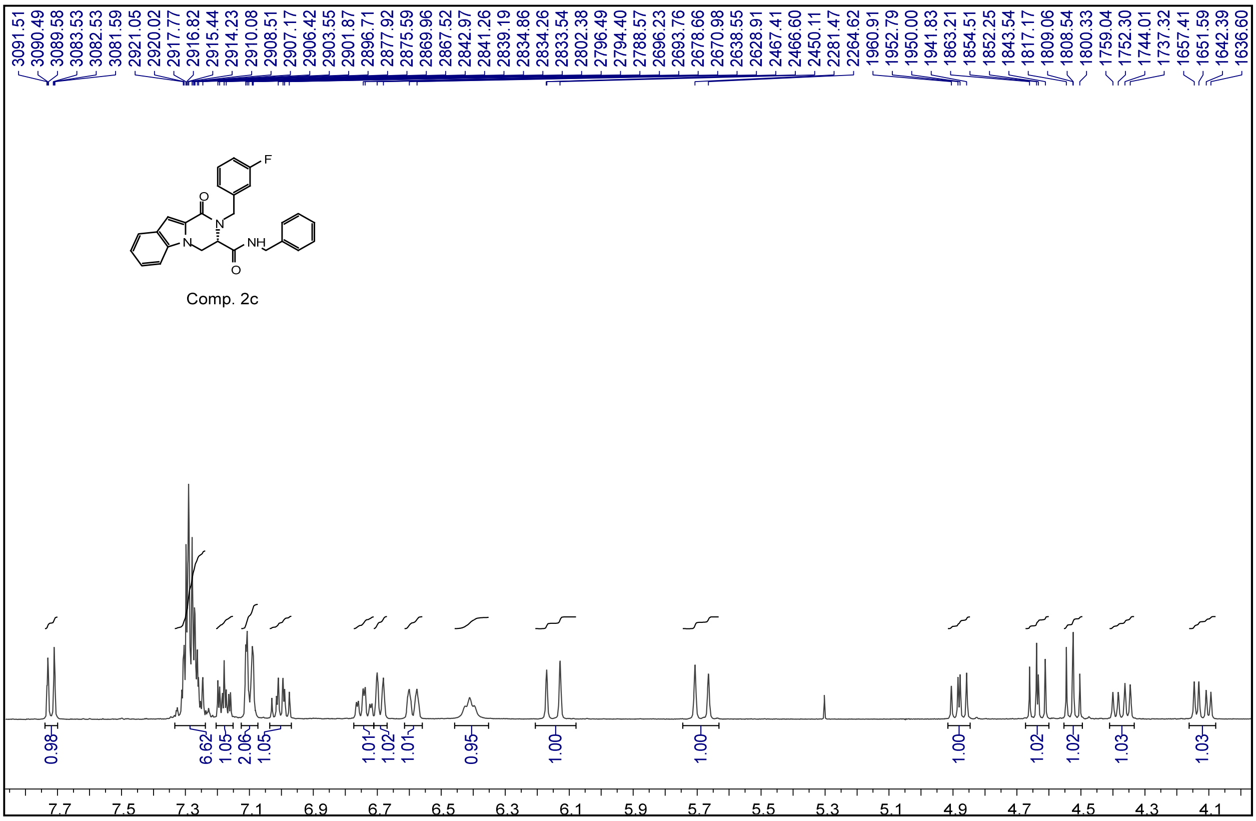

^{13}C NMR: (*S*)-*N*-Benzyl-2-(3-fluorobenzyl)-1-oxo-1,2,3,4-tetrahydropyrazino[1,2-*a*]indole-3-carboxamide (2c)

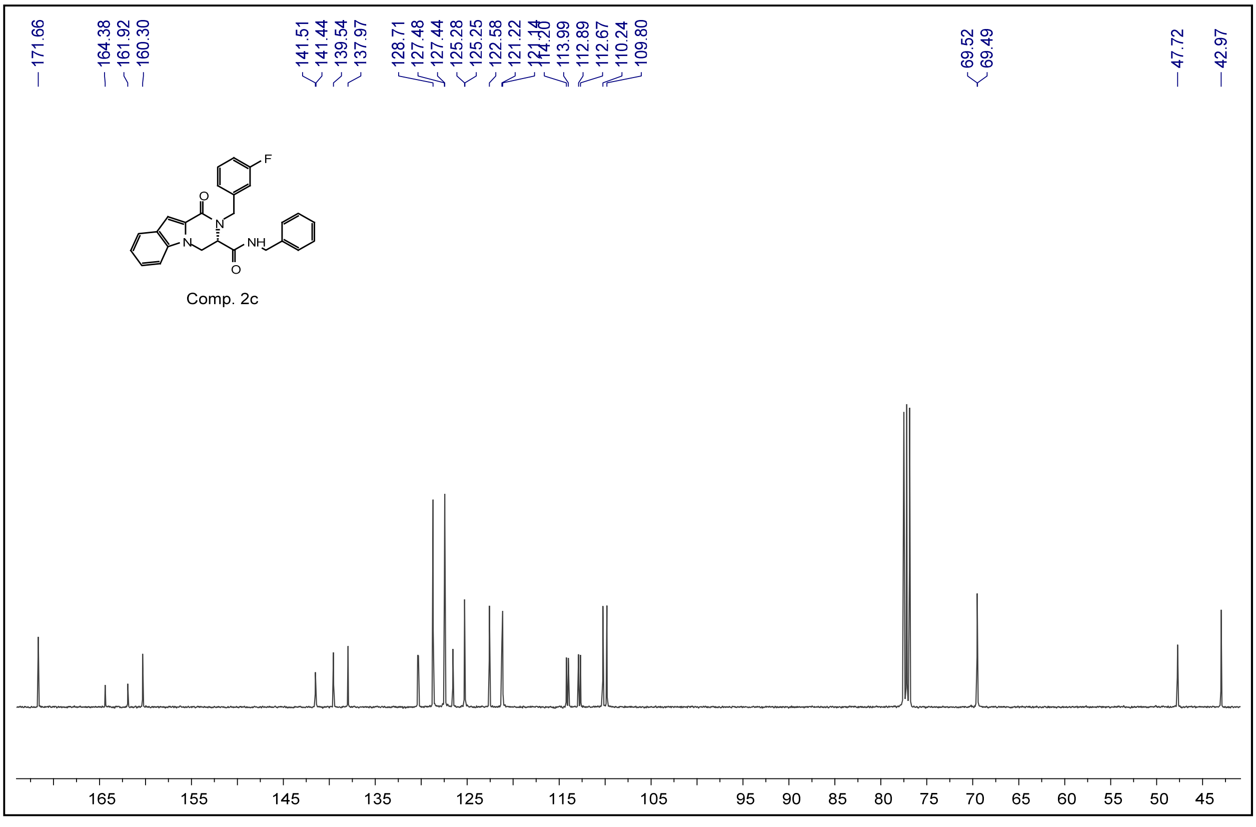

^{1}H NMR: (*S*)-*N*-Benzyl-2-(4-fluorobenzyl)-1-oxo-1,2,3,4-tetrahydropyrazino[1,2-*a*]indole-3-carboxamide (2d)

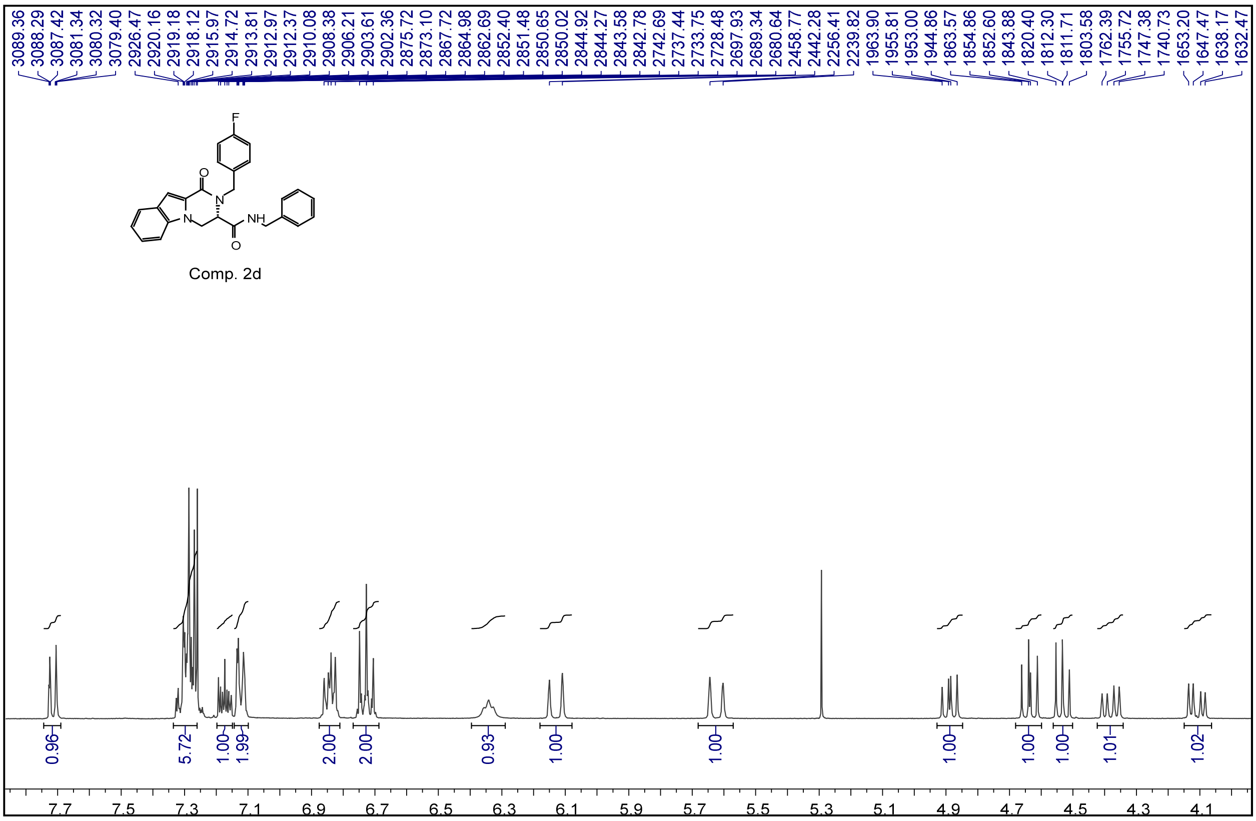

^{13}C NMR: (*S*)-*N*-Benzyl-2-(4-fluorobenzyl)-1-oxo-1,2,3,4-tetrahydropyrazino[1,2-*a*]indole-3-carboxamide (2d)

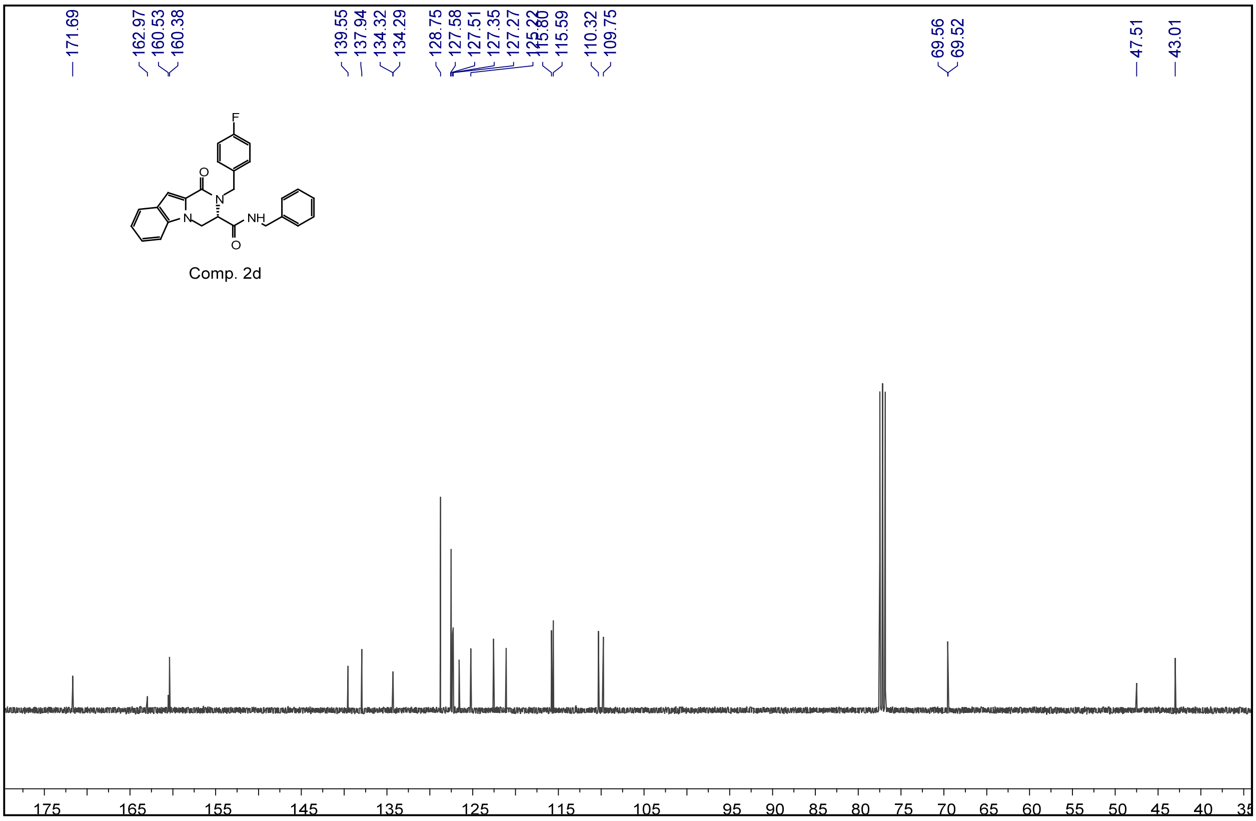

^{1}H NMR: (*S*)-*N*-Benzyl-2-(3-cyanobenzyl)-1-oxo-1,2,3,4-tetrahydropyrazino[1,2-*a*]indole-3-carboxamide (2e)

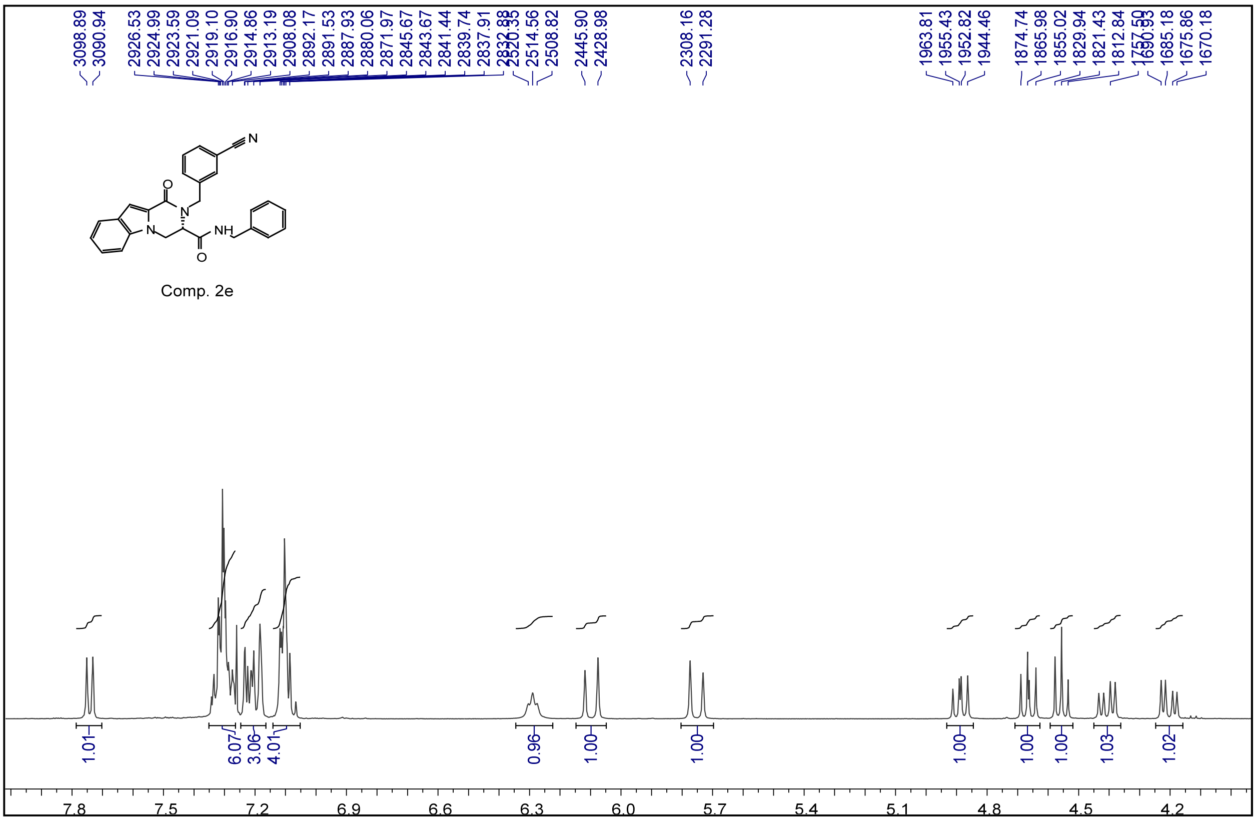

^{13}C NMR: (*S*)-*N*-Benzyl-2-(3-cyanobenzyl)-1-oxo-1,2,3,4-tetrahydropyrazino[1,2-*a*]indole-3-carboxamide (2e)

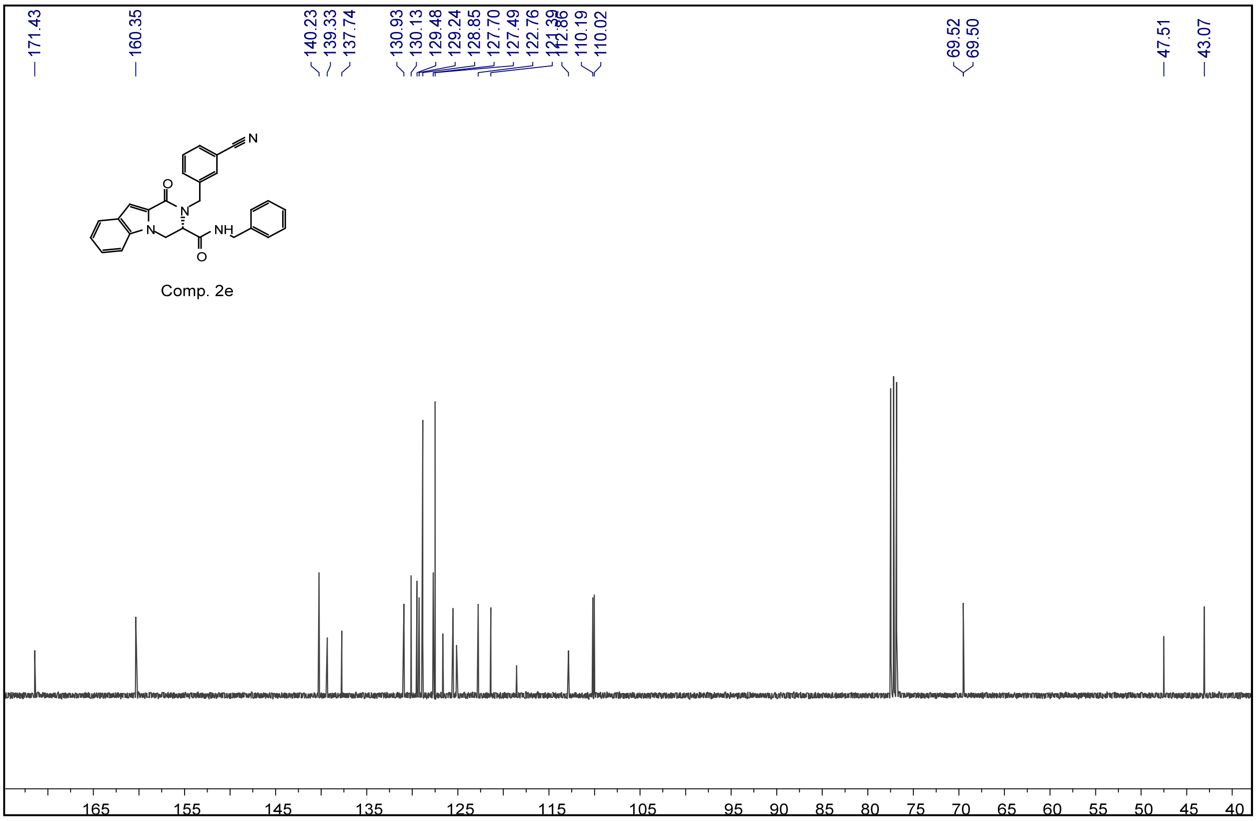

^{1}H NMR: (*S*)-*N*-Benzyl-2-(4-cyanobenzyl)-1-oxo-1,2,3,4-tetrahydropyrazino[1,2-*a*]indole-3-carboxamide (2f)

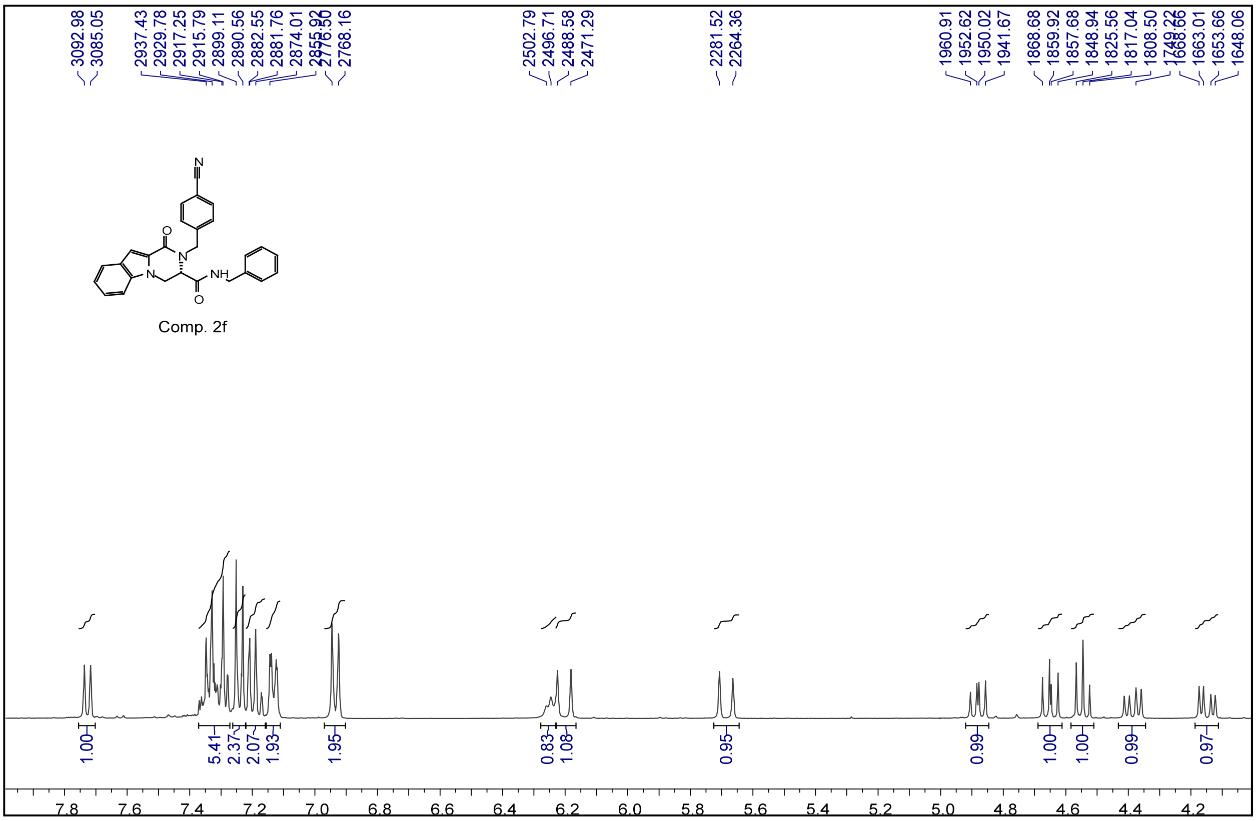

^{13}C NMR: (*S*)-*N*-Benzyl-2-(4-cyanobenzyl)-1-oxo-1,2,3,4-tetrahydropyrazino[1,2-*a*]indole-3-carboxamide (2f)

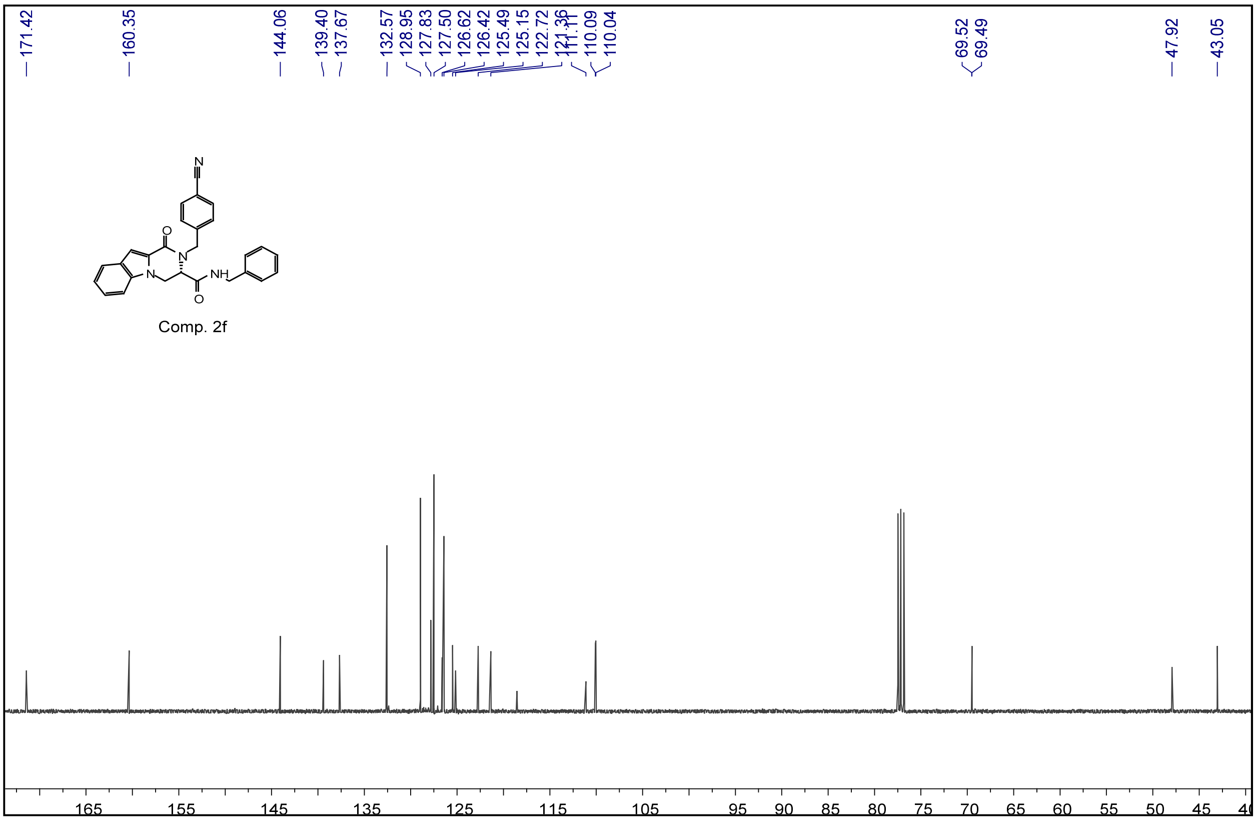

^{1}H NMR: (*S*)-*N*-Benzyl-2-(4-trifluoromethylbenzyl)-1-oxo-1,2,3,4-tetrahydropyrazino[1,2-*a*]indole-3-carboxamide (2g)

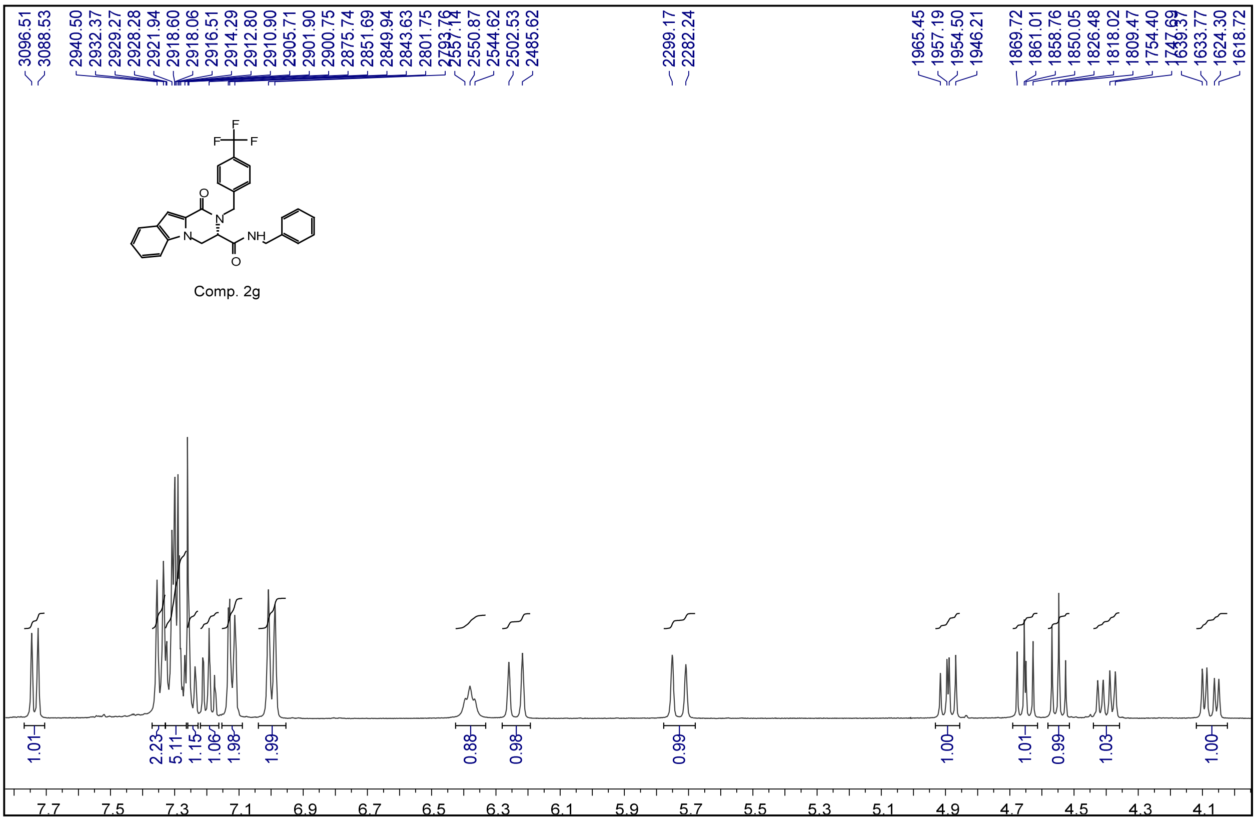

^{13}C NMR: (*S*)-*N*-Benzyl-2-(4-trifluoromethylbenzyl)-1-oxo-1,2,3,4-tetrahydropyrazino[1,2-*a*]indole-3-carboxamide (2g)

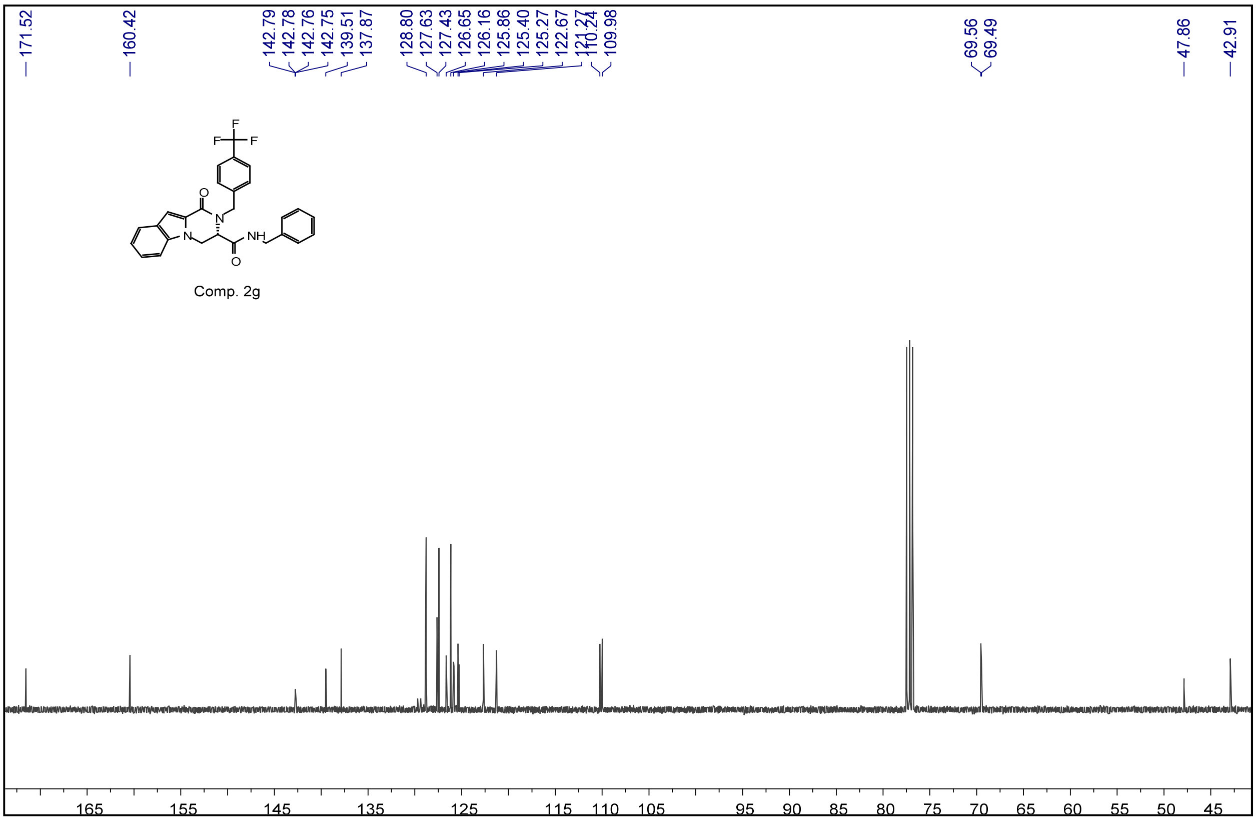

^{1}H NMR: (*S*)-*N*-Benzyl-2-(3-trifluoromethoxybenzyl)-1-oxo-1,2,3,4-tetrahydropyrazino[1,2-*a*]indole-3-carboxamide (2h)

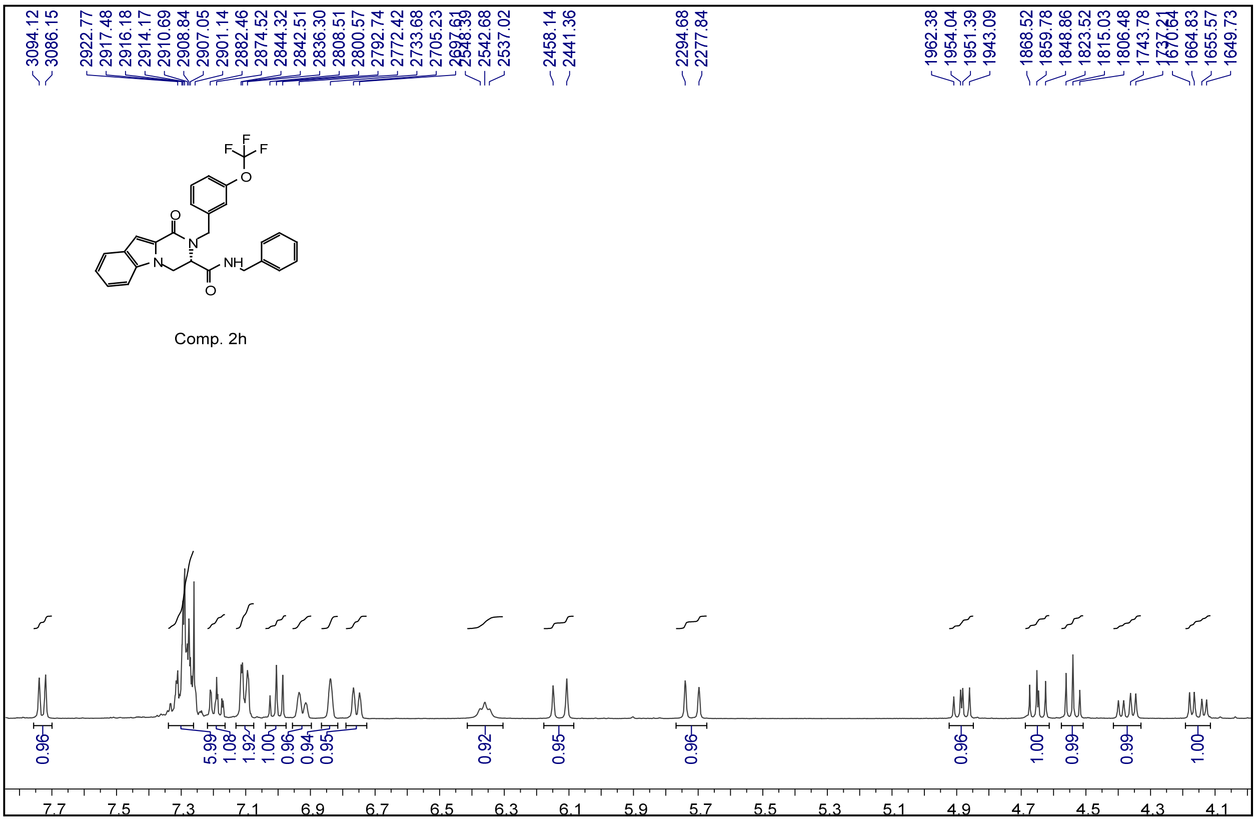

^{13}C NMR: (*S*)-*N*-Benzyl-2-(3-trifluoromethoxybenzyl)-1-oxo-1,2,3,4-tetrahydropyrazino[1,2-*a*]indole-3-carboxamide (2h)

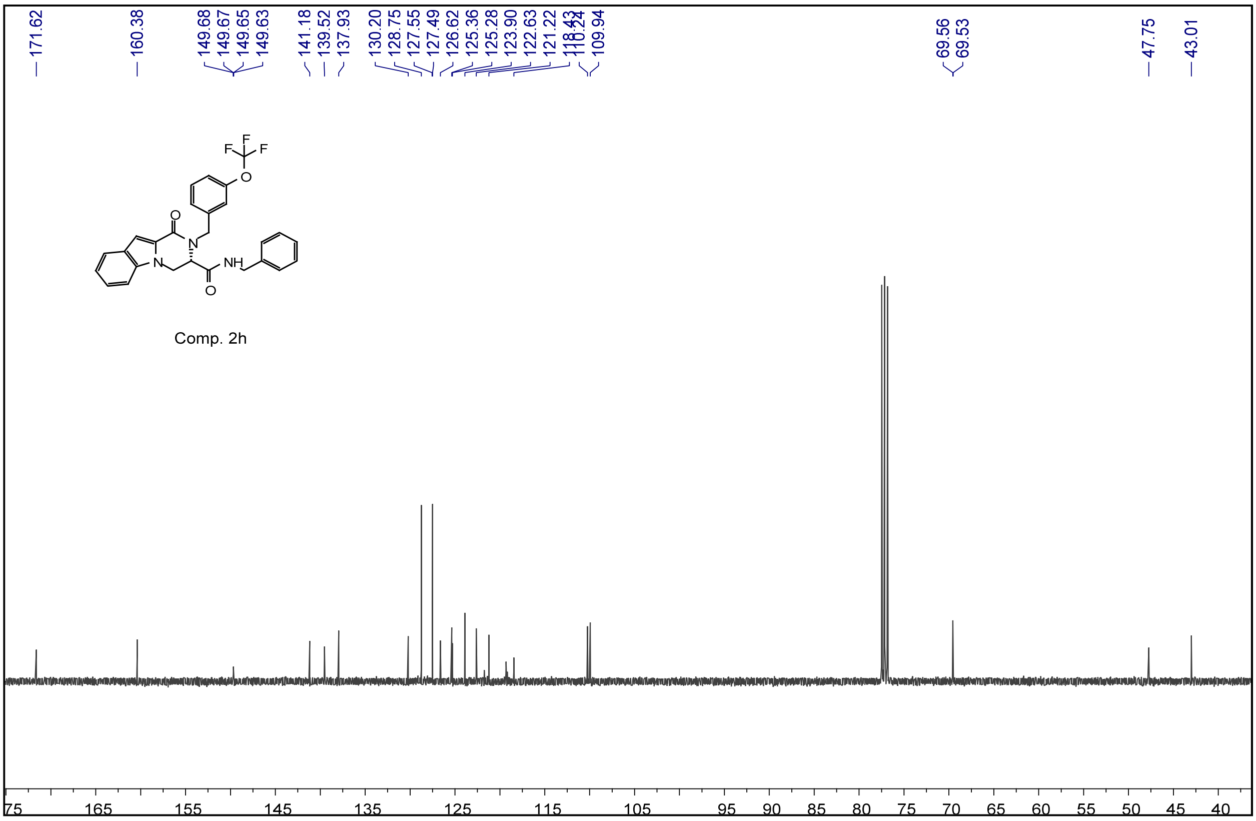

^{1}H NMR: (*S*)-*N*-Benzyl-2-(4-trifluoromethoxybenzyl)-1-oxo-1,2,3,4-tetrahydropyrazino[1,2-*a*]indole-3-carboxamide (2i)

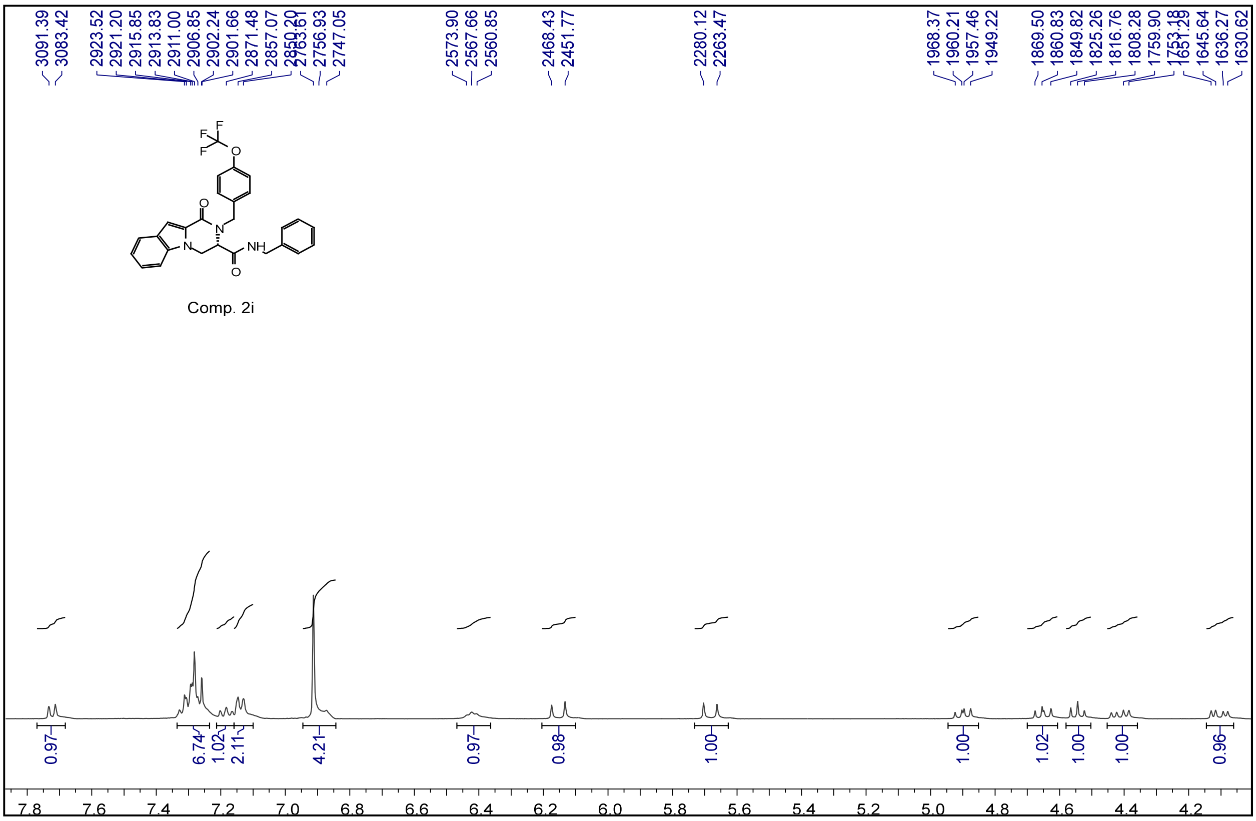

^{13}C NMR: (*S*)-*N*-Benzyl-2-(4-trifluoromethoxybenzyl)-1-oxo-1,2,3,4-tetrahydropyrazino[1,2-*a*]indole-3-carboxamide (2i)

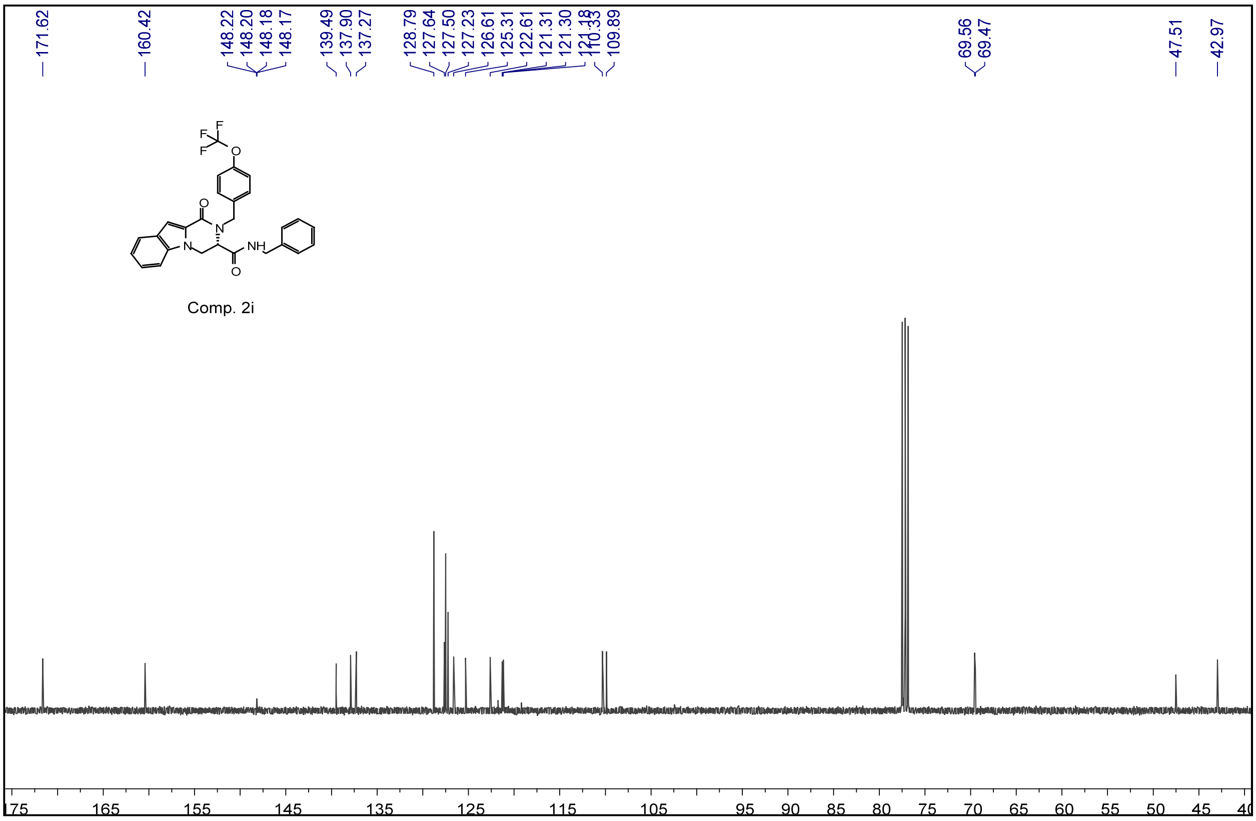

^{1}H NMR: (*S*)-*N*-Benzyl-2-(3-methylbenzyl)-1-oxo-1,2,3,4-tetrahydropyrazino[1,2-*a*]indole-3-carboxamide (2j)

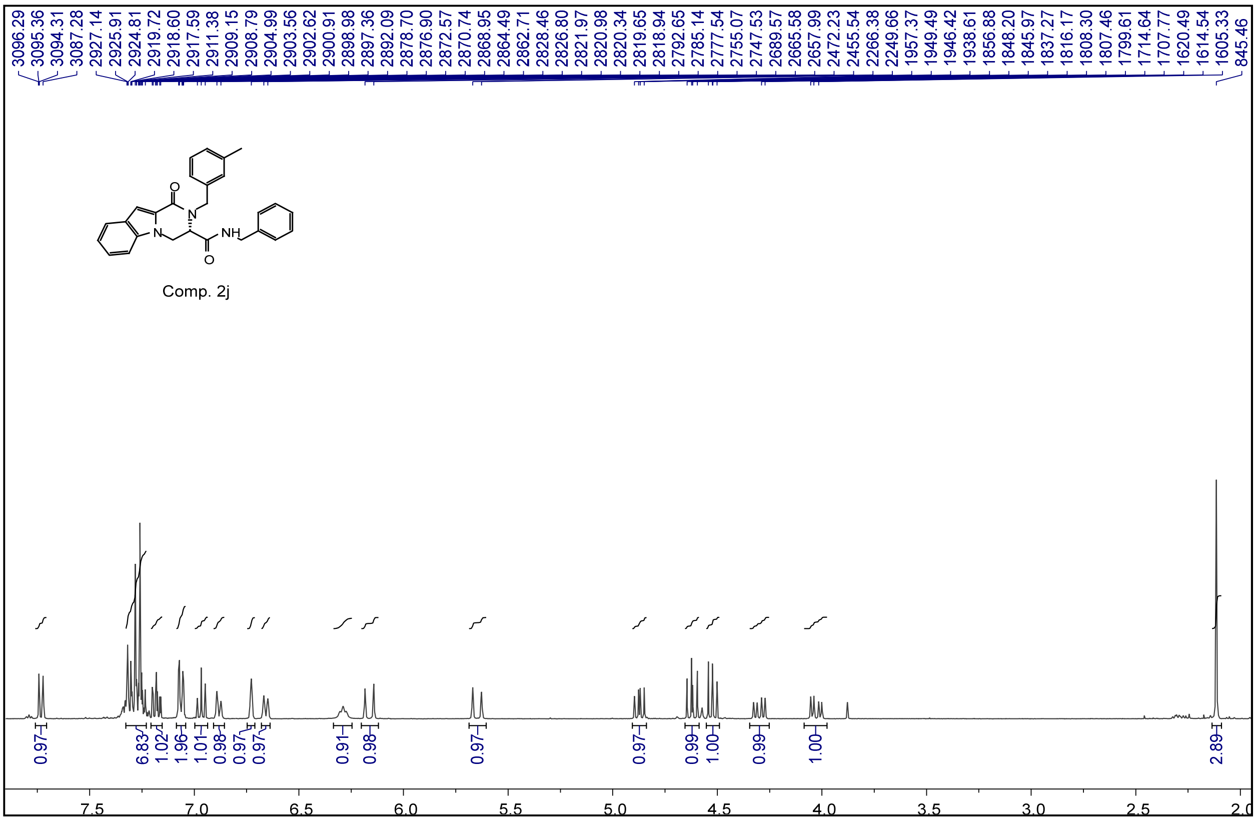

^{13}C NMR: (*S*)-*N*-Benzyl-2-(3-methylbenzyl)-1-oxo-1,2,3,4-tetrahydropyrazino[1,2-*a*]indole-3-carboxamide (2j)

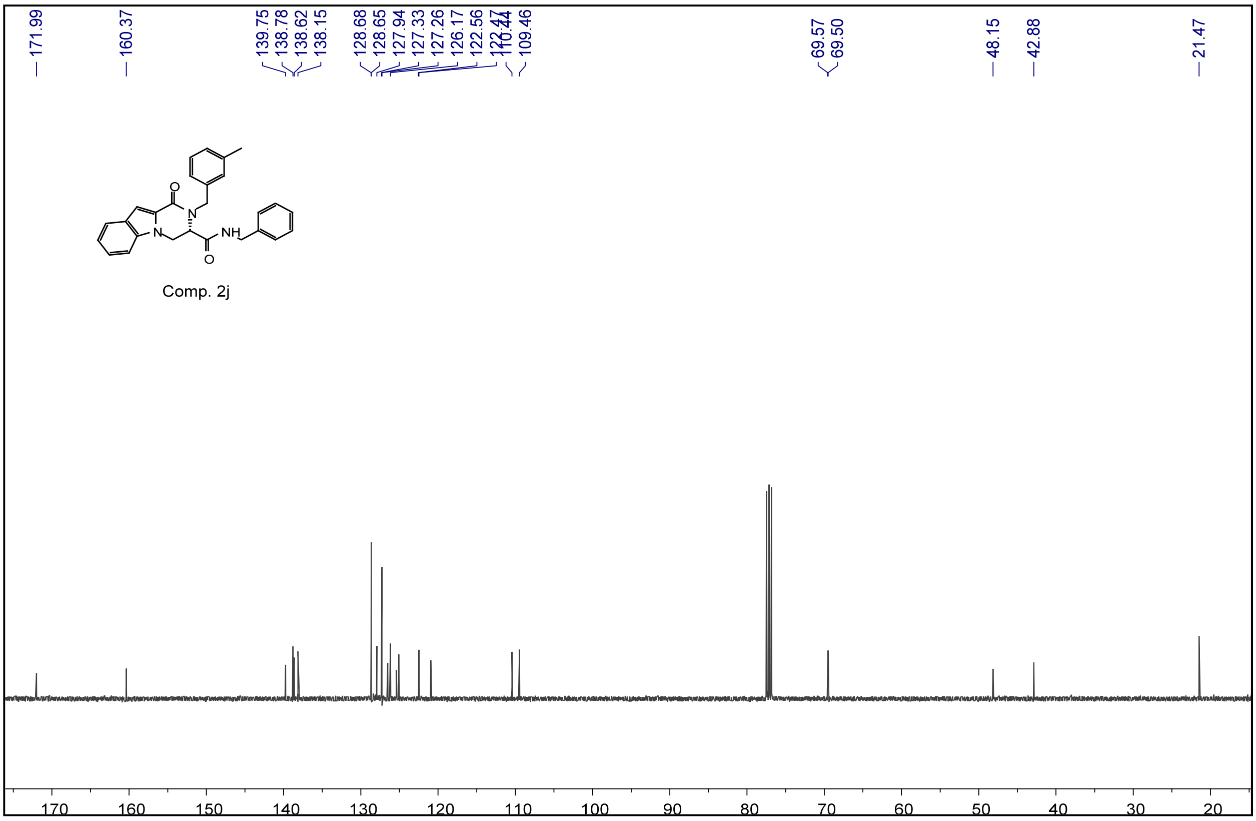

^{1}H NMR: (*S*)-*N*-Benzyl-2-(4-methylbenzyl)-1-oxo-1,2,3,4-tetrahydropyrazino[1,2-*a*]indole-3-carboxamide (2k)

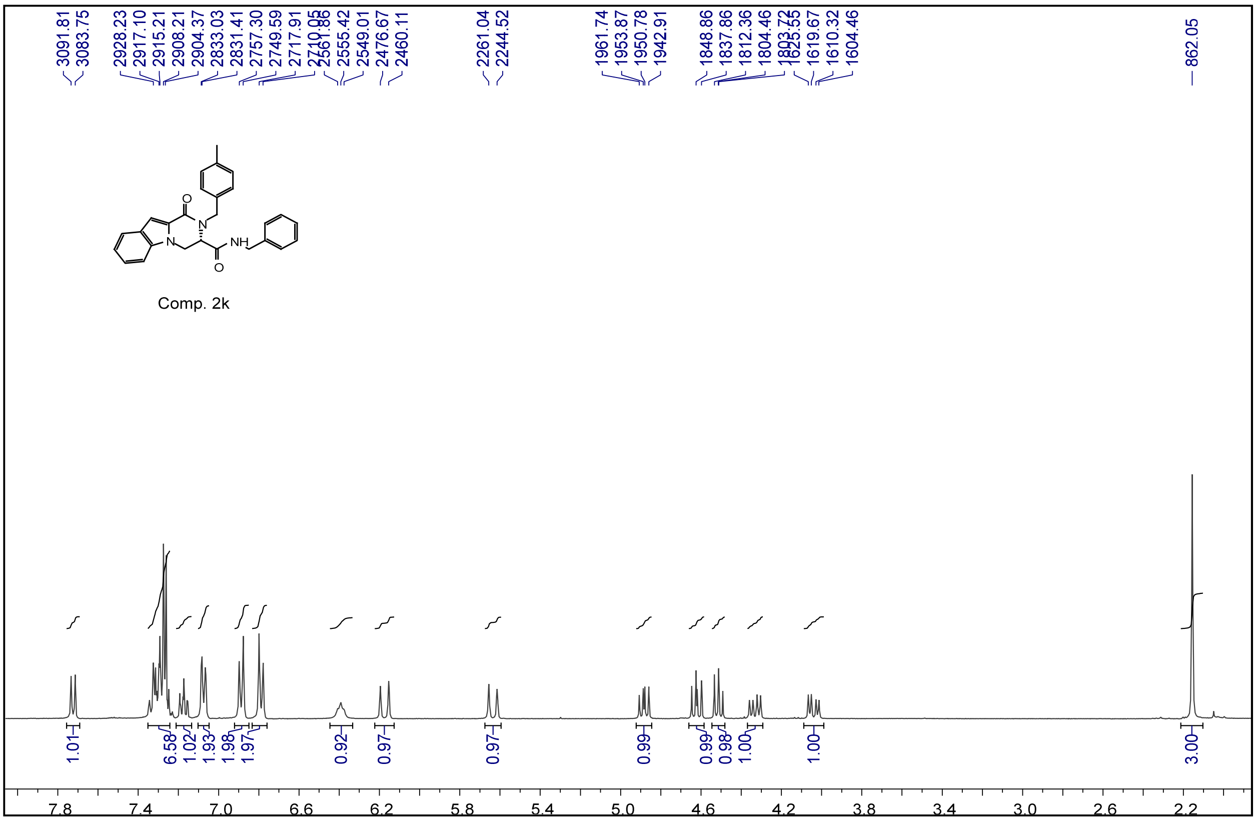

^{13}C NMR: (*S*)-*N*-Benzyl-2-(4-methylbenzyl)-1-oxo-1,2,3,4-tetrahydropyrazino[1,2-*a*]indole-3-carboxamide (2k)

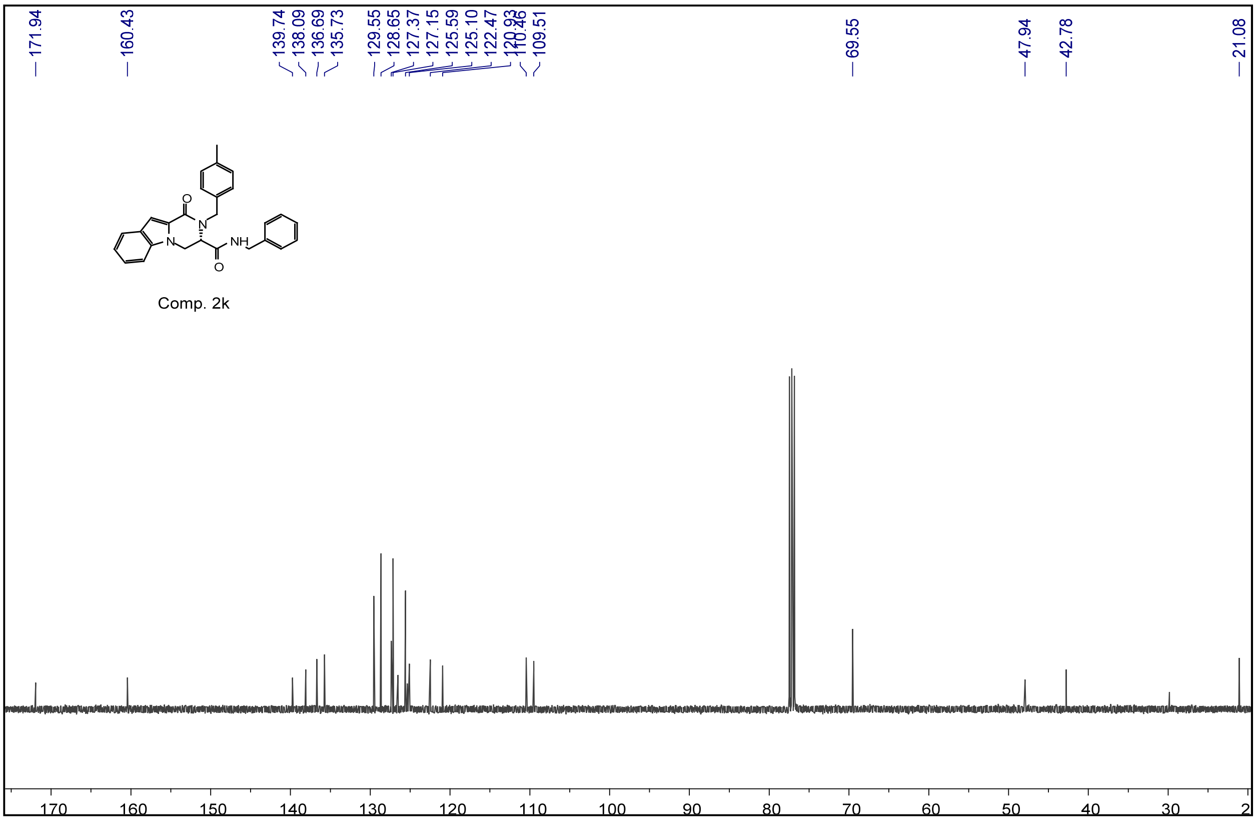

^{1}H NMR: (*S*)-*N*-Benzyl-2-(naphthalen-1-ylmethyl)-1-oxo-1,2,3,4-tetrahydropyrazino[1,2-*a*]indole-3-carboxamide (2l)

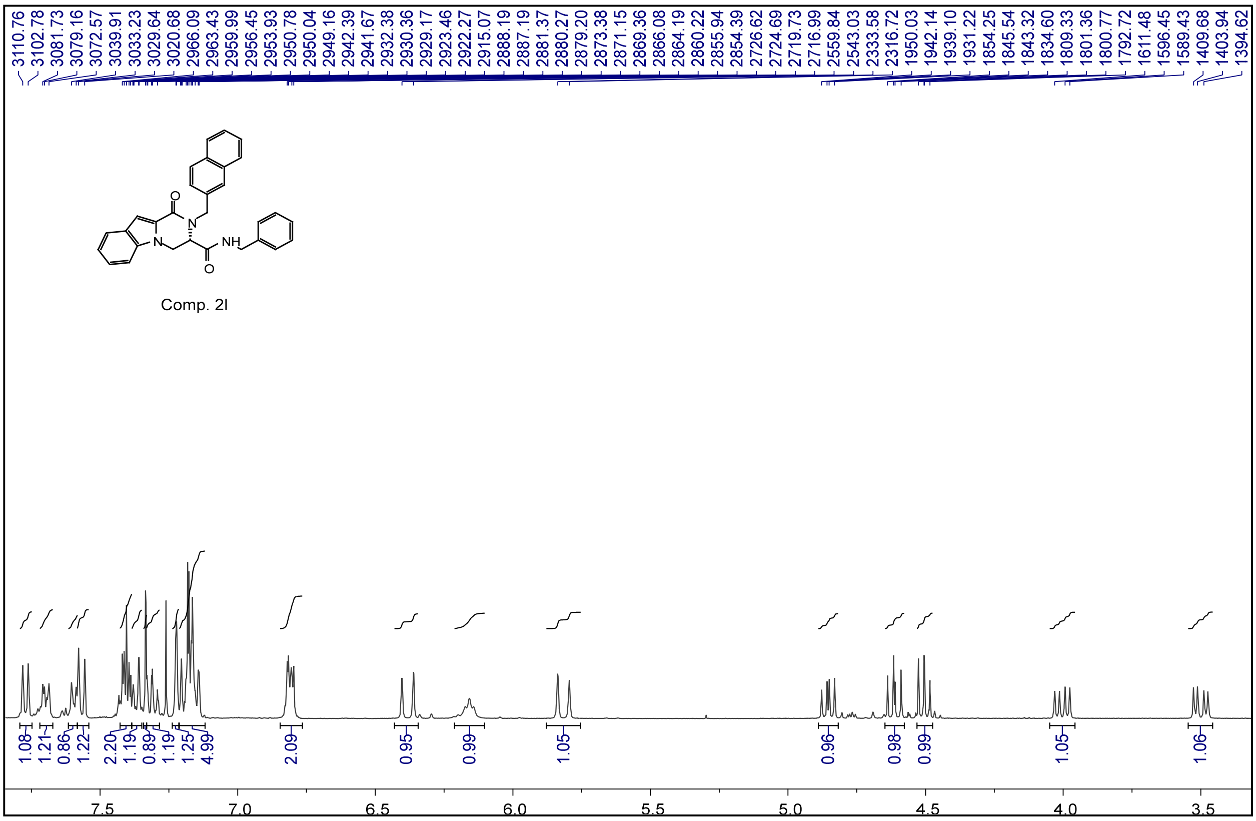

^{13}C NMR: (*S*)-*N*-Benzyl-2-(naphthalen-1-ylmethyl)-1-oxo-1,2,3,4-tetrahydropyrazino[1,2-*a*]indole-3-carboxamide (2l)

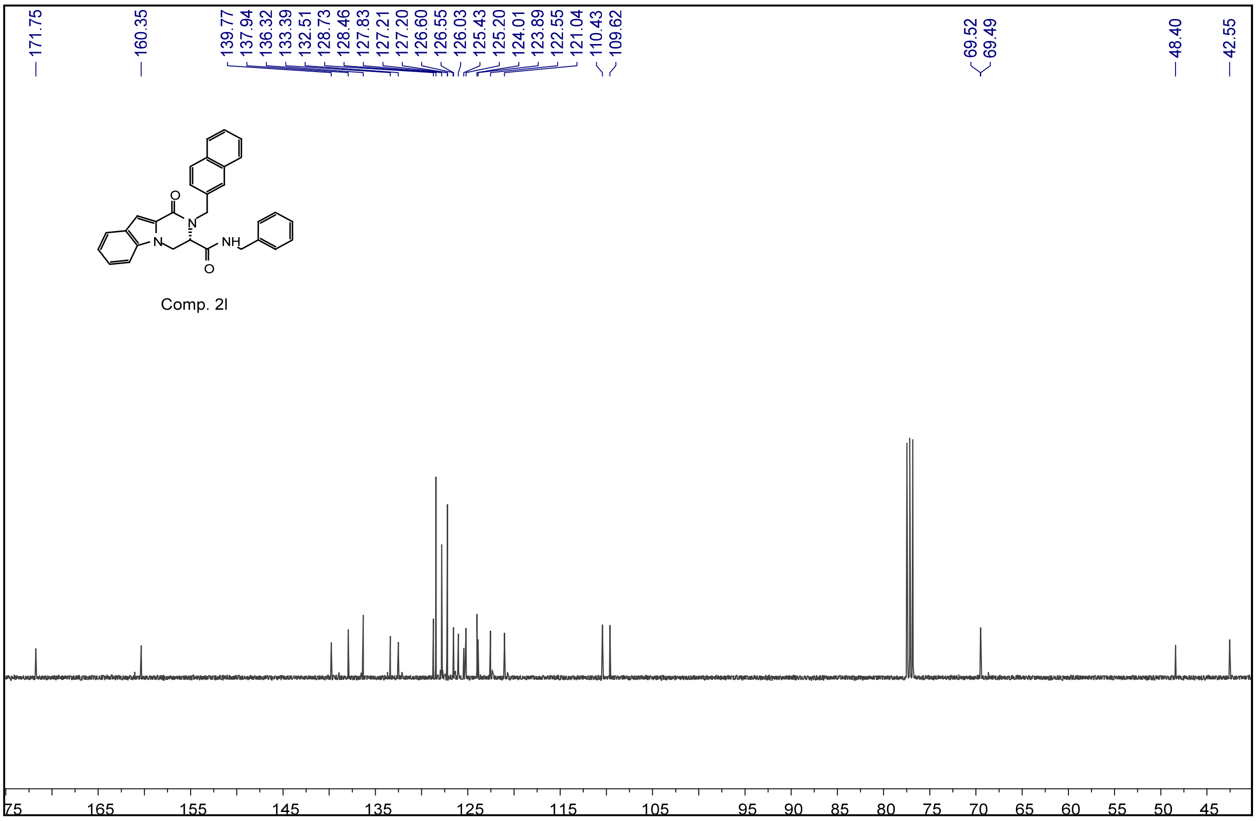

^{1}H NMR: (*S*)-*N*-Benzyl-2-([1,1'-biphenyl]-4-ylmethyl)-1-oxo-1,2,3,4-tetrahydropyrazino[1,2-*a*]indole-3-carboxamide (2m)

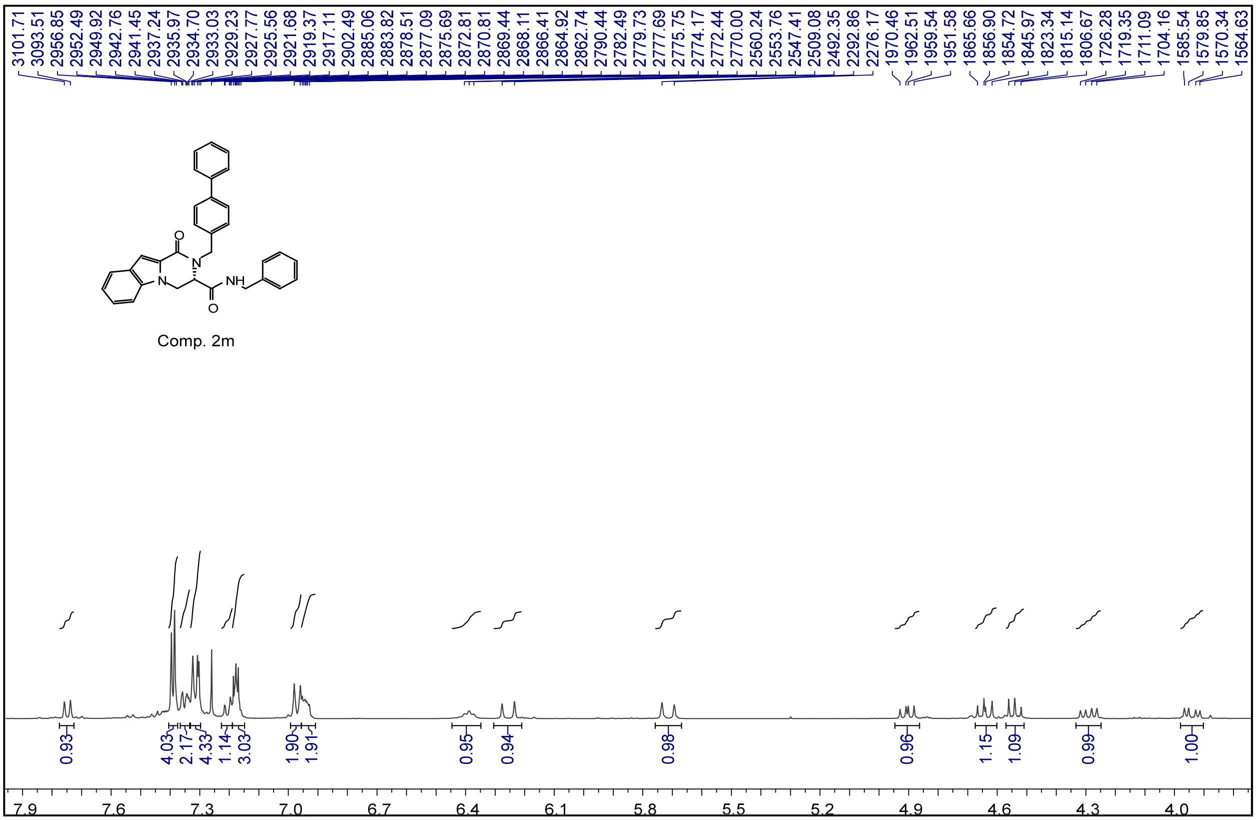

^{13}C NMR: (*S*)-*N*-Benzyl-2-([1,1'-biphenyl]-4-ylmethyl)-1-oxo-1,2,3,4-tetrahydropyrazino[1,2-*a*]indole-3-carboxamide (2m)

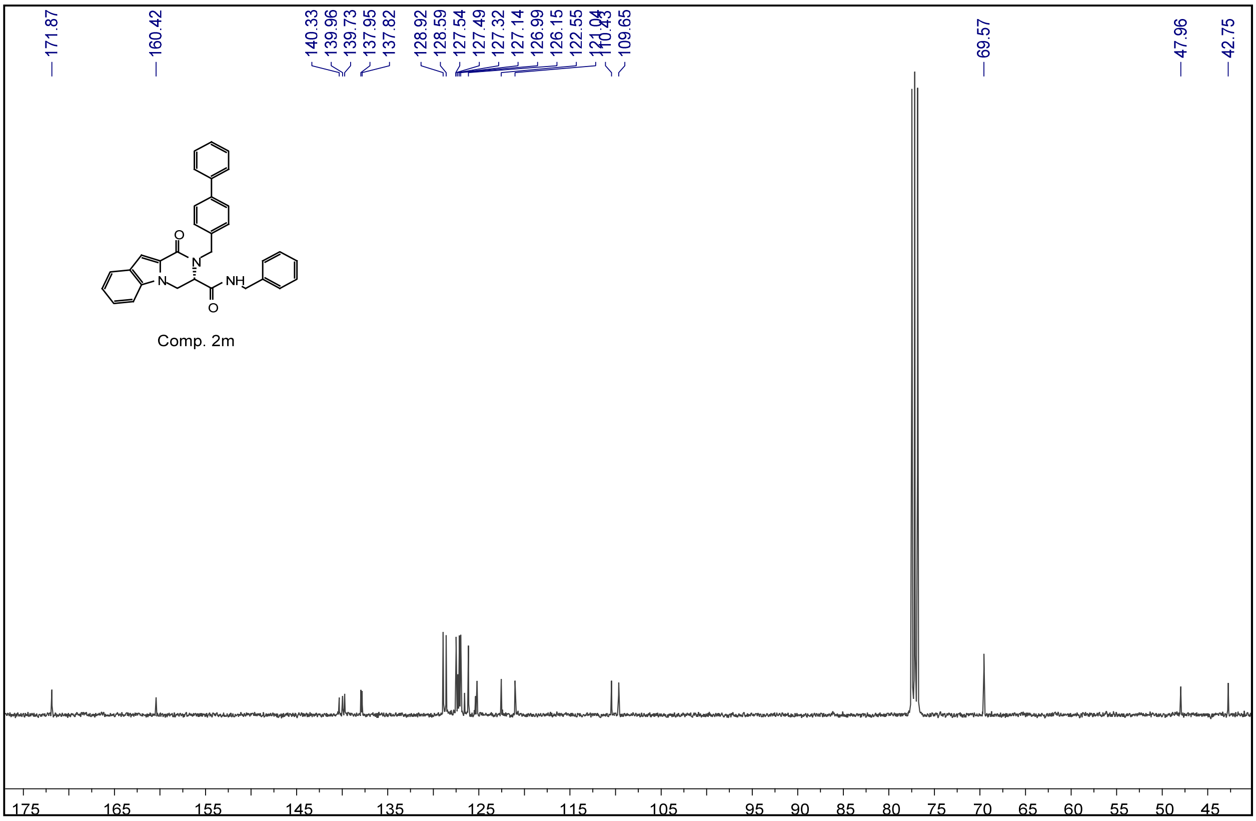

Supplement: Supplementary file 1 [file pharmaceuticals-14-00974-s001.zip › pharmaceuticals-1384707-supplementary.pdf]
